# GPCR kinases shape ACKR4 functions via differential C-terminal phosphorylation

**DOI:** 10.1038/s41467-026-73074-4

**Published:** 2026-05-16

**Authors:** Oliver J. Gerken, Rebecca Warmers, Clara Hild, Niklas Kielkopf, Nicola Catone, Roland Bruderer, Daniel F. Legler

**Affiliations:** 1 Institute of Cell Biology and Immunology Thurgau (BITG) at the University of Konstanz, University of Konstanz, Kreuzlingen, Switzerland; 2 Graduate School for Cellular and Biomedical Sciences, University of Bern, Bern, Switzerland; 3 Biognosys AG, Schlieren, Switzerland; 4 Theodor Kocher Institute, University of Bern, Bern, Switzerland; 5 Department of Biology, University of Konstanz, Konstanz, Germany

**Keywords:** Chemokines, Signal transduction, Biochemistry, G protein-coupled receptors

## Abstract

Atypical chemokine receptor 4 (ACKR4) is a scavenger receptor that regulates the availability of chemokines, including CCL19, and consequently the responsiveness of their classical G protein coupled receptors (GPCRs). In contrast to classical chemokine receptors, ACKR4 is completely biased towards βarrestins and does not couple to G proteins. Here, we show that ACKR4 in its apo state constitutively pre-associates with βarrestins and cycles between the plasma membrane and endosomal compartments. We identify distinct serine and threonine residues in the tail region of ACKR4 involved in regulating steady-state receptor trafficking and chemokine uptake, and that a C-terminal serine/threonine cluster is key for both ligand-mediated βarrestin recruitment and efficient chemokine uptake. Moreover, different serine/threonine clusters in the tail region of ACKR4 account for steady-state and chemokine-driven association of the four non-visual GPCR kinases (GRKs), which differentially phosphorylate two serine and one threonine residues. We show that GRK5/6 primarily phosphorylate ACKR4 in the absence of chemokines, and that CCL19 stimulation recruits GRK2/3 to enhance ACKR4 phosphorylation. Notably, we found that apo ACKR4 forms a ternary complex with GRK2/3 and the G protein without activating it. Finally, we show that the C-terminal serine/threonine cluster of ACKR4 and GRK2 play leading roles in βarrestin recruitment and CCL19 internalisation.

## Abstract (web summary)

ACKR4 is scavenger for chemokines. Here, authors identify a ternary signalling complex comprising receptor, βarrestin and the inactive G protein that facilitates GRK2/3 recruitment and chemokine internalization.

## Introduction

G protein-coupled receptors (GPCRs) form a large family of ~800 members that share an archetypical topology of seven transmembrane domains, are activated by extracellular stimuli, and mainly couple to heterotrimeric G proteins to transduce signals intracellularly. GPCRs recognise a vast diversity of signals ranging from hormones, neurotransmitters, odours, light, peptides, lipids, and chemokines, and hence they form one of the largest drug target families^1^. Typically, ligand binding to its cognate GPCR results in conformational changes across the transmembrane α-helices, enabling the activation of the intracellular associated G protein, i.e. the dissociation of the Gα subunit from the Gβγ subunits, both of which can interact with various effector proteins to propagate intracellular signalling^2,3^.

A long-held tenet is that specialised GPCR kinases (GRKs) are recruited to ligand-activated receptors and phosphorylate serine and threonine residues situated predominantly in the receptor’s C-terminus, or occasionally in its intracellular loop 3 (ICL3), which enables the recruitment of βarrestins to the GPCR^4,5^. Binding of βarrestins to the GPCR can desensitise the receptor by preventing G protein-coupling, promote its endocytosis, and/or contribute to G protein-independent signalling^6,7^. Importantly, the number and arrangement of phosphates vary for a given GPCR and different phosphorylation patterns, referred to as ‘barcodes’, trigger different βarrestin-mediated effects^8^. Notably, the regulation and phosphorylation of non-visual GPCRs is thought to be orchestrated by just four broadly expressed GRKs (GRK2/3/5/6), which share regulatory and catalytic domains, but differ in their C-termini^5^. While GRK2/3 are cytosolic and contain a C-terminal pleckstrin homology (PH) domain enabling PIP_2_ binding, GRK5/6 are attached to the plasma membrane through palmitoylation of cysteine residues and/or via an amphipathic helix interacting with phospholipids^5^. So far, no consensus sequence for the phosphorylation by GRKs has been identified, and it is poorly understood how these four GRKs specifically control hundreds of GPCRs on an individual level.

G protein activation and βarrestin recruitment are considered as two independent mechanisms, and in addition, GPCR signalling can intrinsically be biased towards the G protein or βarrestin pathways^9^. However, the recruitment of GRK2 and GRK3 to activated receptors has been linked to G protein activation, as liberated Gβγ was shown to facilitate GRK2/3 translocation to the membrane through interaction with the C-terminal PH domain of the kinase^6,10^. Recent studies using GRK knock-out cells revealed insights into the role of individual kinases on βarrestin recruitment and GPCR regulation^11,12^. These cellular tools will enable the systematic assessment of GRK involvement in individual GPCRs and may shed light on how the limited number of GRKs controls the various functions of a single receptor within the entire GPCR superfamily.

The GPCR superfamily includes a group of chemokine receptors that orchestrate cell migration during embryonic development, tissue formation, wound repair, host defence against invaders, but also in cancer malignancy and other diseases^13,14^. Chemokine receptors couple to G_i_ proteins to direct cell migration^15,16^. For instance, expression of the chemokine receptor CCR7 is upregulated in dendritic cells upon pathogen encounter, which facilitates their egress from peripheral barrier tissues and subsequent homing via lymphatics to draining lymph nodes along local gradients of its cognate ligands, the chemokines CCL19 and CCL21. CCR7 also controls T cell trafficking through the blood and lymph nodes, in which T cells are searching for pathogen-derived antigens presented by homed dendritic cells. Thus, CCR7-driven leucocyte migration guided by its ligands CCL19 and CCL21 is key for launching adaptive immune responses against invading pathogens^17,18^. Moreover, CCR7 expressed by cancer cells contributes to metastasis formation in lymphoid organs^19^. Remarkably, in different leucocytes, different GRKs are involved in regulating CCR7 signalling and in fine-tuning coordinated cell migration^18,20,21^. Furthermore, GRK3 and GRK6 were proposed to promote ligand-induced βarrestin recruitment and subsequent CCR7 endocytosis via clathrin-coated pits^22,23^.

Notably, a subfamily of atypical chemokine receptors (ACKRs) has been delineated from GPCRs based on their inability to couple to G proteins and hence to mediate cell migration in response to chemokines. However, ACKRs play important roles within the chemokine system by sequestering, transporting or internalising chemokines, thereby regulating their availability and shaping their gradients^24–26^. ACKR4 is best known for shaping functional CCL21 and CCL19 gradients^27–30^. It is expressed by endothelial cells lining lymphatic vessels and stromal cells in various tissues to form local chemokine gradients, thereby facilitating CCR7-driven dendritic cell egress from peripheral tissue, entrance to lymphatics and migration to the T cell zone in lymph nodes, as well as CCR7-guided T cell homing. In addition to CCL19 and CCL21, ACKR4 also binds and takes up the chemokines CCL20, CCL22 and CCL25^31–34^. Mechanistically, ACKR4 was shown to constitutively interact with βarrestins and to cycle between the plasma membrane and endosomal compartments^33–35^, a common characteristic observed for ACKR2-5^24–26^. Chemokine binding to ACKR4 was described to primarily recruit GRK3, resulting in enhanced βarrestin interaction and chemokine scavenging activity^35^. As ACKR4 does not activate G proteins^35^, it remains unclear how GRK3 can boost chemokine uptake. Interestingly, ACKR3, the atypical scavenger receptor for the chemokine CXCL12, was shown to become phosphorylated by GRK5 and GRK2^36,37^, whereas GRK2 activity was proposed to happen either G protein-independent^36^ or indirectly and in cooperation with the canonical CXCL12 receptor CXCR4^37,38^. Notably, these pioneer studies on ACKRs rely on C-terminally tagged receptors. Recently, we uncovered that C-terminal tagging of ACKR4 provoked an elevated pre-association of βarrestins with the plasma membrane, yet a reduced chemokine-mediated βarrestin recruitment, thereby affecting the chemokine scavenging activity of ACKR4^39^. It is unclear whether native ACKR4 recruits GRK3 alike tagged receptor, and how this would occur, given that GRK2/3 are believed to depend on G protein activation. In addition, it is currently unknown whether other GRKs are recruited to native ACKR4, which residues are phosphorylated, and how different phosphorylation sites potentially regulate the scavenging activity of ACKR4.

In this study, we identify distinct serine and threonine residues in the tail region of ACKR4 involved in regulating steady-state receptor trafficking and chemokine uptake, and that a C-terminal cluster of serine/threonine residues controls ligand-mediated βarrestin recruitment and chemokine internalisation. We demonstrate that different serine/threonine clusters account for steady-state and chemokine-driven association of the four GRKs. We show that GRK5/6 primarily phosphorylate ACKR4 in the absence of chemokines, and that CCL19 stimulation recruits GRK2/3 to enhance ACKR4 phosphorylation. We uncover that apo ACKR4 forms a ternary complex with GRK2/3 and the G protein without activating it. Finally, we show that terminal serine/threonine residues of ACKR4 and GRK2 play a leading role in βarrestin recruitment and chemokine internalisation.

## Results

### ACKR4 efficiently senses CCL19 and facilitates endosomal localisation of βarrestins

**Fig. 1:**
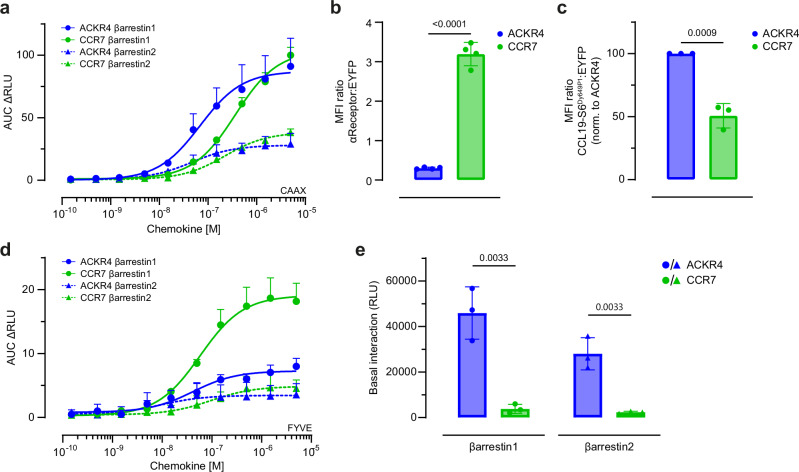
Major differences in CCL19 sensing and βarrestin recruitment by ACKR4 and CCR7. **a** Recruitment of βarrestins to the plasma membrane residing CAAX in response to native, untagged ACKR4 (blue) or CCR7 (green) upon stimulation with graded concentrations of CCL19. HEK293 cells transiently co-expressing smBiT-βarrestin1 (or smBiT-βarrestin2), lgBiT-CAAX together with either untagged ACKR4 or CCR7, were stimulated with CCL19. Chemokine concentration-dependent βarrestin recruitment to the plasma membrane based on nanoBiT reconstitution was recorded. *n* = 3, mean ± SD. **b** MFI ratio between EYFP expression and surface staining of co-expressed ACKR4 or CCR7; *n* = 4, mean ± SD. **c** Chemokine internalisation by transiently transfected cells expressing either EYFP and ACKR4, or EYFP and CCR7. Depicted is the MFI ratio between EYFP and fluorescently labelled CCL19 (5 nM) after 30 min of stimulation, normalised to the uptake by ACKR4; *n* = 3, mean ± SD. **d** Indirect βarrestin recruitment to ACKR4 or CCR7 at early endosomes (FYVE domain) upon stimulation with increasing concentrations of CCL19 and **e** baseline interaction of βarrestins with the early endosome marker FYVE in the presence of ACKR4 or CCR7; *n* = 3, mean ± SD. Statistical analysis: **b**, **c**, **e** unpaired two-sided *t* test; exact *P* values are shown in the graphs.

Scavenging of the chemokine CCL19 is controlled by constantly cycling ACKR4, and receptor trafficking correlates with βarrestin association, although ligand uptake persists in cells lacking βarrestins^35^. Commonly, βarrestin recruitment and GPCR trafficking are assessed by nanoluciferase and/or BRET-based assays using tagged receptors. We recently uncovered that C-terminal tagging of ACKR4 led to a pre-association of βarrestins with the plasma membrane, which resulted in reduced chemokine-mediated βarrestin recruitment and altered scavenging activity^39^. This discovery necessitates re-assessing βarrestin recruitment and receptor trafficking of native, untagged ACKR4. To achieve this, we established an indirect, bystander split-luciferase assay, in which we fused the larger fragment of the split-nanoluciferase (referred to as lgBiT) to the plasma membrane anchor CAAX and the small peptide fragment of the split-nanoluciferase (smBiT) to βarrestins. This allowed us to study CCL19-driven recruitment of βarrestins to the plasma membrane upon stimulation of ACKR4, or its canonical receptor CCR7, in cells expressing native, untagged receptors (Supplementary Fig. 1a–c). ACKR4 was more sensitive to CCL19 stimulation than CCR7, as βarrestin1 and βarrestin2 recruitment to the plasma membrane occurred at lower chemokine concentrations (Fig. 1a); EC_50(ACKR4:βarr1)_ = 69 nM, EC_50(CCR7:βarr1)_ = 366 nM, EC_50(ACKR4:βarr2)_ = 46 nM, EC_50(CCR7:βarr2)_ = 224 nM. Moreover, CCL19 was superior in recruiting βarrestin1 than βarrestin2 in response to ACKR4 and CCR7 triggering (Fig. 1a). To control expression levels of native, untagged receptors, we used IRES vectors to express EYFP together with ACKR4 or CCR7 and specific antibodies for surface staining. As expected for a constitutively cycling receptor, only a fraction of ACKR4 was detected at the cell surface under steady-state conditions (i.e. in the absence of a ligand), whereas CCR7 was readily present at the cell surface (Fig. 1b). Nonetheless, ACKR4 was significantly more efficient in taking up fluorescently labelled CCL19-S6^Dy649P1^ than CCR7 over a period of 30 min (Fig. 1c). Interestingly, CCL19 stimulation of CCR7 led to a profound trafficking of smBiT-βarrestin to early endosomes marked with lgBiT-FYVE (Fig. 1d). In contrast, CCL19-mediated recruitment of β-arrestins to early endosomes was substantially lower for ACKR4 than for CCR7 (Fig. 1d). However, under steady-state conditions, we noted a marked pre-association of βarrestin1 and βarrestin2 with early endosomes in cells expressing ACKR4, but not in those expressing CCR7 (Fig. 1e), suggesting that ACKR4 constitutively traffics conjointly with βarrestins from the plasma membrane to early endosomes. These data indicate that ACKR4, in its apo state (i.e. without a bound ligand), interacts constitutively with βarrestins and constantly traffics between the plasma membrane and endosomal compartments. While reaching the cell surface, apo-ACKR4 efficiently senses cognate chemokines and internalises them on the way to endosomes.

### Putative phosphorylation sites at ACKR4’s tail are critical for efficient chemokine uptake

**Fig. 2:**
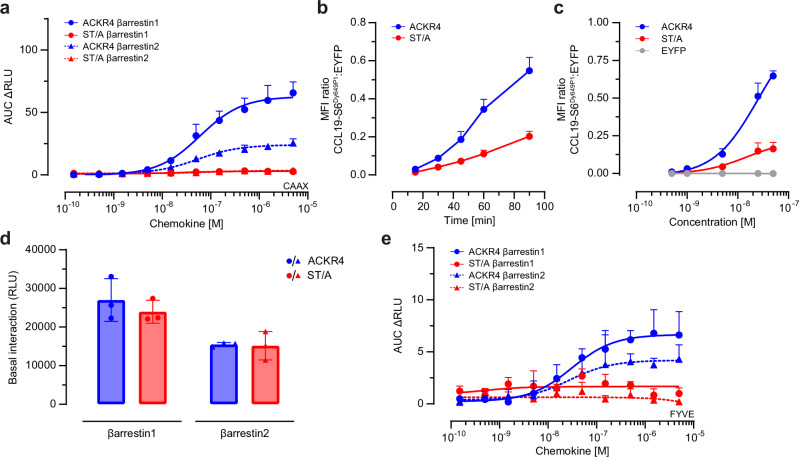
Serine/threonine residues in the tail of ACKR4 distinctly regulate βarrestin recruitment and CCL19 internalisation. **a** Dose-dependent smBiT-βarrestin recruitment to lgBiT-CAAX at the plasma membrane in the presence of ACKR4 (blue) or ACKR4 ST/A (red); *n* = 3, mean ± SD. **b** Chemokine uptake in HEK293 cells expressing ACKR4 or ACKR4 ST/A incubated with 5 nM CCL19-S6^Dy649P1^ over the course of 90 min, depicting the MFI ratio between the internalised chemokine and the co-expressed EYFP; *n* = 3, mean ± SD. **c** MFI ratio of transiently transfected HEK293 cells expressing either EYFP and ACKR4, EYFP and ACKR4 ST/A or EYFP, stimulated with increasing amounts of fluorescently labelled CCL19 for 30 min; *n* = 3, mean ± SD. **d** Basal interaction of smBiT-βarrestins with lgBiT-FYVE at early endosomes in the presence of ACKR4 or ACKR4 ST/A; *n* = 3, mean ± SD. **e** Bystander smBiT-βarrestin recruitment to lgBiT-FYVE at early endosomes upon stimulation of ACKR4 or ACKR4 ST/A with increasing concentrations of CCL19; *n* = 3, mean ± SD.

We set out to identify the residues responsible for the constitutive association of ACKR4 with βarrestins. It is known that βarrestins interact with phosphorylated serine and threonine residues in the tail of GPCRs. Hence, we mutated all potential phosphorylation sites, i.e., serine and threonine residues, located within the C-terminus of ACKR4 to alanine residues, referred to as ACKR4 ST/A. As expected, the ACKR4 ST/A mutant failed to recruit βarrestin1 and βarrestin2 to the plasma membrane in response to CCL19 stimulation, even at the highest tested chemokine concentration of 5 μM (Fig. 2a). Surprisingly, ACKR4 ST/A was still able to take up fluorescent CCL19-S6^Dy649P1^ in a time dependent manner, although less efficient than wild-type ACKR4 (Fig. 2b). Incubating ACKR4 ST/A-expressing cells with graded concentrations of CCL19-S6^Dy649P1^ indicated that chemokine uptake reached a plateau at 25 nM CCL19, whereas native ACKR4 steadily continued to internalise CCL19 also at higher concentrations (Fig. 2c). Interestingly, βarrestins comparably pre-associated with FYVE-positive endosomes in ACKR4 and ACKR4 ST/A-expressing cells in the absence of ligand (Fig. 2d), but only wild-type receptor and not its mutant form was able to recruit additional βarrestins upon CCL19 stimulation (Fig. 2e). In addition, we noted higher surface expression of ACKR4 than ACKR4 ST/A (Supplementary Fig. 2a), whereas the basal association of βarrestins with CAAX was similar in cells expressing ACKR4 and ACKR4 ST/A (Supplementary Fig. 2b). Collectively, these data suggest that ACKR4 in its apo state constantly traffics together with βarrestins, or at least with βarrestin-bearing endosomes, and while doing so is able to co-internalise CCL19. Moreover, our data provide evidence that serine/threonine residues in the tail of ACKR4 are responsible for CCL19-mediated βarrestin recruitment and efficient chemokine uptake.

### Distinct C-terminal serine and threonine residues regulate steady-state trafficking and chemokine-induced internalisation of ACKR4

**Fig. 3:**
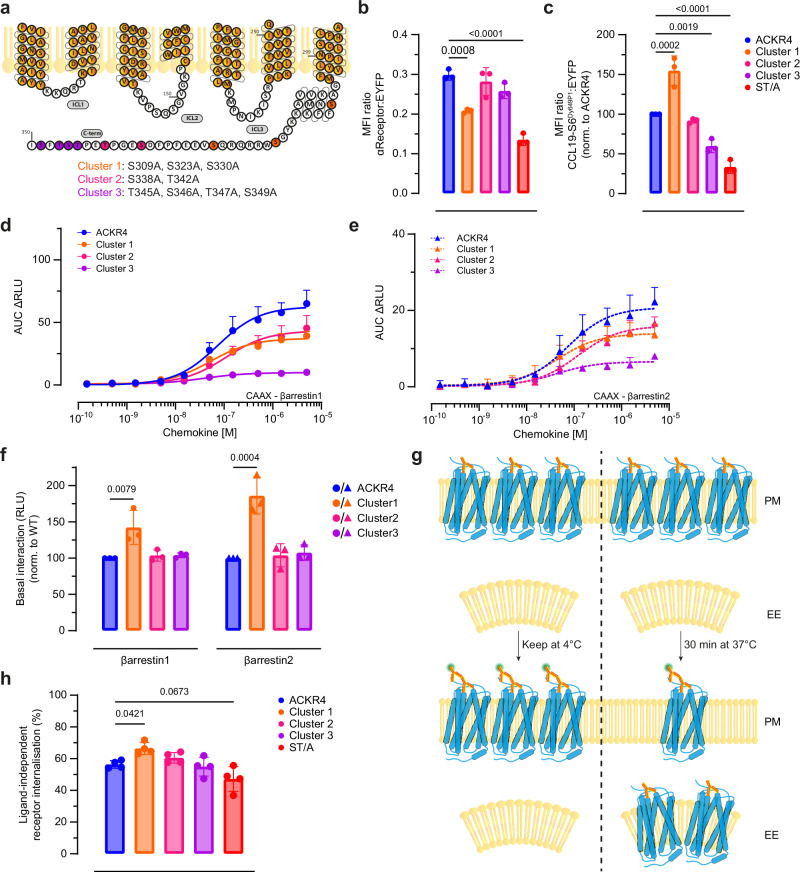
Distinct Ser/Thr clusters in the C-terminus of ACKR4 contribute to steady-state trafficking and chemokine-induced receptor internalisation. **a** Illustration of the secondary structure of ACKR4 visualising the residues exchanged in the different cluster mutants. **b** Surface receptor staining and **c** chemokine uptake (5 nM for 30 min) of transiently transfected HEK293 cells expressing wild-type ACKR4 or a receptor mutant, normalised to the co-expressed transfection control EYFP (MFI ratio); *n* = 3, mean ± SD. **d** Ligand-induced bystander smBiT-βarrestin1 and **e** smBiT-βarrestin2 recruitment to lgBiT-CAAX at the plasma membrane in HEK293 cells expressing ACKR4 or different receptor variants upon addition of increasing concentrations of CCL19. **f** Basal interaction of smBiT-βarrestins with lgBiT-CAAX under steady-state conditions and in the presence of the different receptor variants normalised to wild-type ACKR4; *n* = 3, mean ± SD. **g** Schematic overview of the conducted experiment to study the ligand-independent receptor internalisation (PM plasma membrane, EE early endosomes). **h** Ligand-independent internalisation of wild-type ACKR4 and the different receptor mutants, *n* = 4, mean ± SD. Statistical analysis: **b**, **c**,** f**, **h** ordinary one-way ANOVA; exact *P* values are shown in the graphs.

Next, we aimed at dissecting the role of distinct putative phosphorylation sites for receptor trafficking and chemokine uptake. The C-terminus of ACKR4 includes three clusters of serine/threonine residues, one proximal to transmembrane domain 7/helix 8 (which we refer to Cluster 1), a distal one (Cluster 2) and a terminal one (Cluster 3). We generated variants where the serine/threonine residues within a cluster were mutated to alanines as illustrated in Fig. 3a. We used again IRES vectors to control expression levels of the ACKR4 mutants. Whereas wild-type ACKR4, Cluster 2 and Cluster 3 mutants were similarly expressed at the cell surface, Cluster 1 mutant and ACKR4 ST/A showed reduced surface appearance (Fig. 3b). Interestingly, Cluster 1 mutant internalised 1.5 times more CCL19-S6^Dy649P1^ than wild-type ACKR4 or Cluster 2 mutant, whereas Cluster 3 mutant and ACKR4 ST/A showed significantly decreased chemokine uptake (Fig. 3c). Cluster 1 and Cluster 2 mutants lacking putative phosphorylation sites were still able to recruit βarrestin1 (Fig. 3d) and βarrestin2 (Fig. 3e) to CAAX in response to CCL19 stimulation, although less efficient than wild-type ACKR4. The terminal Cluster 3 mutant barely recruited βarrestins to the plasma membrane upon CCL19 addition (Fig. 3d, e), which is in line with its hampered ability to internalise its ligand. Steady-state association of βarrestins with CAAX was comparable among the different ACKR4 variants except for Cluster 1 mutant, which showed an increased pre-association (Fig. 3f). This observation hints towards an elevated steady-state trafficking activity of the Cluster 1 mutant. To further assess this, we labelled surface receptors in their apo state with a primary monoclonal anti-ACKR4 antibody at 4 °C where steady-state trafficking is prevented. The sample was split into two groups; one was kept at 4 °C, while the other one was incubated for 30 min at 37 °C to facilitate ligand-independent receptor internalisation. Remaining surface residing, labelled receptor was subsequently stained with a fluorescently labelled secondary antibody and analysed by flow cytometry (Fig. 3g). This enabled us to determine the percentage of receptor that internalised in a ligand-independent manner. In support of our notion, slightly more Cluster 1 mutant was internalised in a ligand-independent manner as compared to wild-type ACKR4 and the other Cluster mutants (Fig. 3h). Thus, our mutagenesis study revealed that distinct serine and threonine residues in the tail of ACKR4 are involved in regulating steady-state receptor trafficking and chemokine uptake. Thereby, the terminal serine/threonine cluster in ACKR4 is key for both ligand-mediated βarrestin recruitment and efficient chemokine uptake.

### GPCR kinases exhibit differential targeting of ACKR4 C-terminal regions

**Fig. 4:**
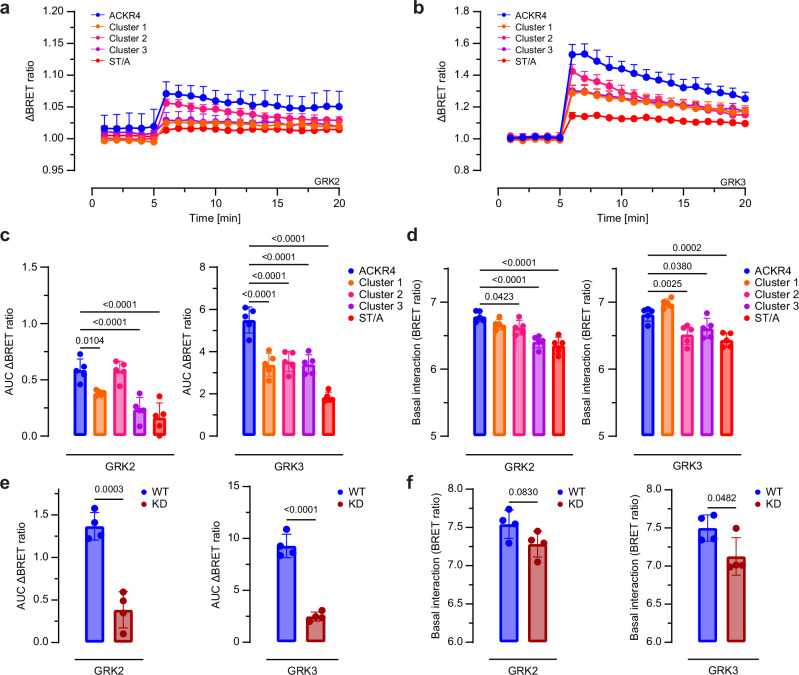
GRK2/3 differentially interact with distinct Ser/Thr clusters of ACKR4 in its apo and chemokine-bound state. **a** Indirect GRK2-rLuc8 and **b** GRK3-rLuc8 recruitment over time to rGFP-CAAX in the presence of wild-type ACKR4 or various receptor mutants before and after addition at *t* = 5 min of 1 μM CCL19; ΔBRET ratios were normalised to PBS. *n* = 5, mean ± SD. **c** Quantification of ligand-induced, integrated ΔBRET ratios over a period of 15 min (AUC) for rGFP-CAAX and GRK2-rLuc8 or GRK3-rLuc8. **d** Basal, steady-state interaction (BRET ratio) of rGFP-CAAX and GRK2-rLuc8 or GRK3-rLuc8 in cells expressing ACKR4 variants. *n* = 5, mean ± SD. **e** Bystander recruitment of wild-type (WT) and kinase-dead (KD) GRK2/3 to rGFP-CAAX upon treatment with 1 μM CCL19 and **d** the corresponding baseline interaction in the absence of ligand, *n* = 4, mean ± SD. Statistical analysis: **c**, **d** ordinary one-way ANOVA, **e**, **f** unpaired two-sided *t* test; exact *P* values are shown in the graphs.

So far, we have identified three clusters with potential phosphorylation sites that contribute to ACKR4 trafficking and chemokine uptake. Next, we aimed to identify which GRK(s) are involved in these processes. Therefore, we exploited a bystander BRET assay with GRKs fused to rLuc8 as BRET donor and rGFP-CAAX as BRET acceptor in cells expressing untagged ACKR4 variants. We observed an increase in ΔBRET between rGFP-CAAX and GRK2-rLuc8 (Fig. 4a) or GRK3-rLuc8 (Fig. 4b) in cells expressing wild-type ACKR4 upon CCL19 stimulation, indicating chemokine-mediated recruitment of GRK2 and GRK3 to the plasma membrane. Changes in the ΔBRET ratio between stimulated and unstimulated cells for GRK2 and GRK3 were profoundly reduced in ACKR4 ST/A-expressing cells (Fig. 4a, b), which aligns with a previous observation regarding ACKR3 ST/A^36^, and the proposed potential interaction of GRKs with the receptor’s tail and core^4^. CCL19-driven GRK2 recruitment was primarily reduced in Cluster 1 and Cluster 3 mutants (Fig. 4c). Chemokine-mediated GRK3 recruitment was reduced in all three Cluster mutants, although the decrease was not as pronounced as of ACKR4 ST/A (Fig. 4c). Investigating basal, steady-state association of GRKs with receptors at the plasma membrane revealed that GRK2 pre-association was mostly reduced in Cluster 3 mutant and that pre-association of GRK3 was impaired in Cluster 2 and Cluster 3 mutants, but not in Cluster 1 mutant (Fig. 4d). We extended our analysis by using known kinase-dead (KD) versions of each GRK^40^, namely GRK2 K220R and GRK3 K220R, and determined their recruitment to CAAX in response to ACKR4 stimulation. Both GRK-KD mutants were significantly less recruited to the plasma membrane in response to CCL19 treatment as compared to their wild-type GRKs (Fig. 4e). Similar results were obtained for steady-state interactions of GRK2/3 and their KD mutants with CAAX (Fig. 4f). We also observed a subtle increase in ΔBRET between rGFP-CAAX and GRK6-rLuc8, but not with GRK5-rLuc8, in ACKR4 expressing cells upon CCL19 stimulation (Supplementary Fig. 3). We further noted changes in BRET under steady-state conditions and differences in ΔBRET between wild-type and ACKR4 variants, as well as wild-type and kinase dead GRK6 (Supplementary Fig. 3). As GRK5/6 predominantly localise at the plasma membrane^5^, these changes in BRET and ΔBRET are presumably due to changes in proximity and/or orientation to each other, which needs alternative methods to the bystander BRET assay for further investigation. Nevertheless, the bystander approach is suitable to conclude that GRK2 and GRK3, but not the KD-mutants, are actively recruited to the plasma membrane upon ACKR4 activation, which is consistent with the finding that GRK2, but not its KD-mutant, is recruited to ACKR3 upon ligand stimulation^36^. Moreover, we show that different serine/threonine residues/clusters at the C-terminus of ACKR4 are involved in steady-state and CCL19-driven association of different GRKs.

### GRK2/3 recruitment occurs independently of G protein activation

**Fig. 5:**
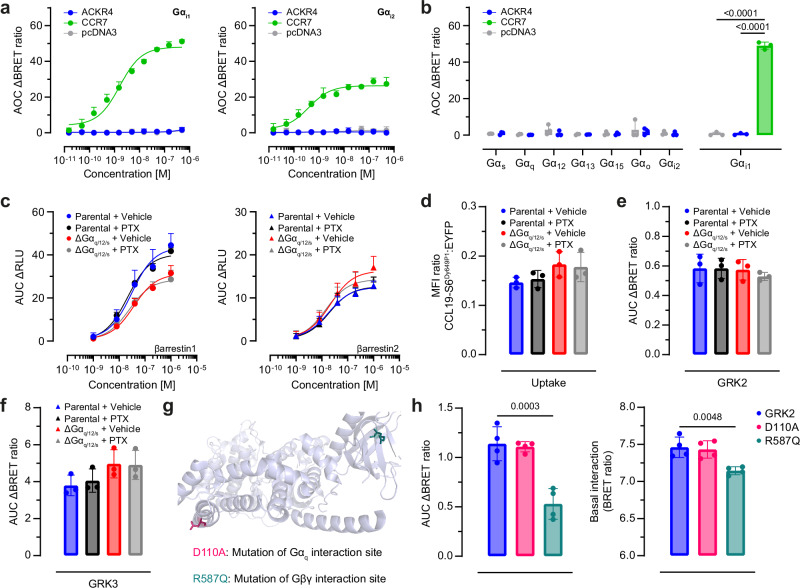
G protein coupling is dispensable for GRK2/3 recruitment to ACKR4 and subsequent chemokine uptake. **a** G protein activation of two heterotrimeric G protein complexes, Gα_i1_ and Gα_i2_, upon addition of graded amounts of CCL19 to HEK293 cells expressing either ACKR4, CCR7 or pcDNA3 empty vector, shown is the area over the curve (AOC) of the associated ΔBRET ratio; *n* = 3, mean ± SD. **b** Determination of further G protein activation of Gα_o_, Gα_s_, Gα_q_, Gα_12_, Gα_13_ and Gα_15_ upon stimulation with 150 nM CCL19 in cells expressing pcDNA3 or ACKR4. For comparison, Gα_i2_, as well as Gα_i1_ in the presence of CCR7 from (**a**) are shown; *n* = 3, mean ± SD. **c** Dose-dependent indirect smBiT-βarrestin recruitment to lgBiT-CAAX in response to CCL19 stimulation of ACKR4 in HEK293A parental and HEK293A ΔGα_q/12/s_ cells in the absence or presence of pertussis toxin (PTX); *n* = 3, mean ± SD. **d** MFI ratio of chemokine uptake (5 nM for 30 min) and co-expressed EYFP; *n* = 3, mean ± SD. **e** AUC ΔBRET ratio of indirect GRK2-rLuc8 or **f** GRK3-rLuc8 recruitment to rGFP-CAAX in HEK293A parental and HEK293A ΔGα_q/12/s_ cells transiently expressing ACKR4 and stimulated with CCL19; *n* = 3, mean ± SD. **g** Partial structure of GRK2 (adapted from: PDB file 6u7c) and localisation of the critical residues responsible for the interaction with Gα_q_ and Gβγ. **h** Bystander recruitment of wild-type GRK2, GRK2 D110A and GRK2 R587Q to ACKR4 stimulated with 1 μM CCL19 and basal interaction of GRK variants in the absence of ligand; *n* = 4, mean ± SD. Statistical analysis: **b**, **h** ordinary one-way ANOVA; exact *P* values are shown in the graphs.

The recruitment of GRK2 and GRK3 to a GPCR is assumed to involve G protein activation, as the liberated Gβγ subunit was originally shown to facilitate GRK2/3 translocation to the membrane for the β_2_-adrenergic receptor^10,41^. Like other ACKRs, ACKR4 was shown not to interact with G proteins and not to activate G_i_ proteins in response to chemokine stimulation^35^. In light of GRK2 and GRK3 being recruited in response to ACKR4 stimulation, we assessed the G protein-coupling abilities of the receptor by monitoring Gβγ dissociation from their Gα subunit. However, CCL19 stimulation did not liberate Gβγ from Gα_i1_ or Gα_i2_ upon binding to ACKR4 but readily did upon engagement with CCR7 (Fig. 5a). In fact, no Gβγ liberation from eight different Gα subunits (Gα_s_, Gα_q_, Gα_12_, Gα_13_, Gα_15_, Gα_o_, Gα_i1_, Gα_i2_) was detected upon ACKR4 engagement by CCL19 (Fig. 5b). To corroborate these findings, we next used HEK293 ‘zero functional G’ cells, where the Gs/q/12 families of Gα proteins were depleted by CRISPR/Cas9 (ΔGα_q/12/s_), along with pertussis toxin (PTX)-mediated inactivation of Gi/o^42^. First, we assessed βarrestin recruitment to the plasma membrane upon ACKR4 stimulation. CCL19-driven recruitment of βarrestin1 was only slightly reduced in HEK293 ΔGα_q/12/s_ cells treated with PTX or its vehicle, whereas the recruitment of βarrestin2 was marginally increased as compared to parental cells (Fig. 5c), indicating that chemokine-mediated G protein coupling is dispensable for βarrestin recruitment to ACKR4. Similarly, uptake of fluorescent CCL19-S6^Dy649P1^ was readily observed in PTX-treated ΔGα_q/12/s_ cells (Fig. 5d). Moreover, CCL19-mediated GRK2 and GRK3 recruitment to ACKR4 proximal CAAX was normal in HEK293 cells lacking functional G proteins (Fig. 5e, f). Finally, we assessed two GRK2 mutants known to interfere either with Gα or Gβγ interaction **(**Fig. 5g). GRK2 D110A was shown to inhibit binding to free Gα_q_, and consequently was not recruited to the plasma membrane if co-expressed with constitutive active free Gα_q_^43^, whereas GRK2 R587Q was reported to impair the interaction with Gβγ^41^. Neither chemokine-induced recruitment nor steady-state association with CAAX was altered by introducing the GRK2 D110A mutation (Fig. 5h). Notably, steady-state interaction of GRK2 R587Q with CAAX and its recruitment were significantly reduced (Fig. 5h), suggesting that the βγ-subunit of the G protein is somehow involved in membrane association of GRK2. Collectively, these data indicate that the recruitment of GRK2 and GRK3 to ACKR4 is independent of G protein activation and that GRK2 can interact with the heterotrimeric G protein without the need of its activation.

### ACKR4, GRK2 and the G protein form a ternary complex

**Fig. 6:**
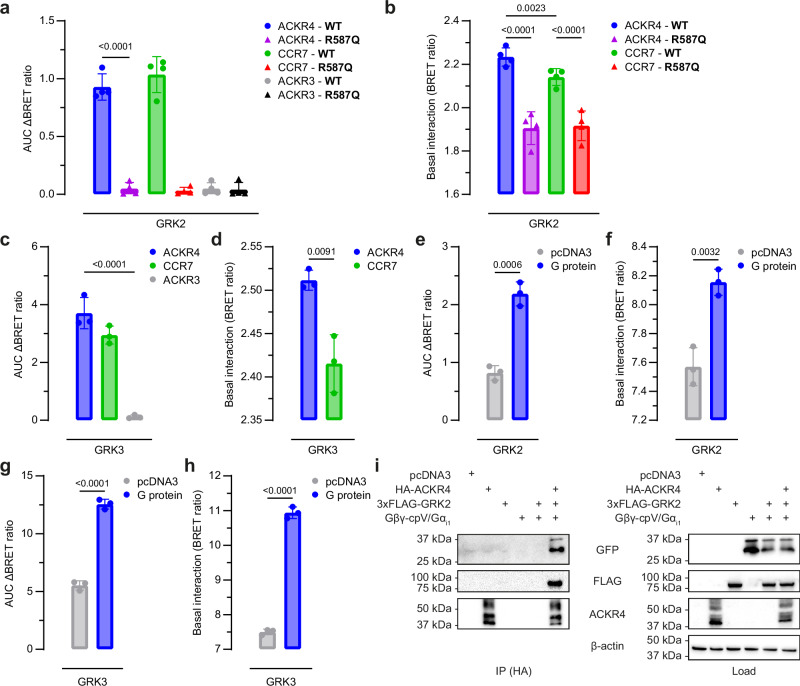
ACKR4 forms a ternary complex with GRK2 and the heterotrimeric G protein. **a** Quantification (AUC) of changes in ΔBRET ratios between GRK2 and Gβγ in response to 1 μM CCL19 stimulation in HEK293 cells co-expressing GRK2 (WT or R587Q)-nLuc, all three subunits of the G protein (Gα_i1_/Gβ/Gγ, the latter is fused to cpVenus) together with either ACKR4, CCR7, or ACKR3 (serving as negative control), and **b** corresponding basal interaction (BRET ratio) before chemokine addition; *n* = 4, mean ± SD. **c** CCL19-induced (1 μM) association of GRK3 and G proteins in HEK293 cells transiently expressing GRK3-nLuc, Gβγ-cpVenus/Gα_i1_, together with either ACKR4, CCR7 or ACKR3, and **d** related basal interaction between GRK3 and G proteins; *n* = 3, mean ± SD. **e** CCL19-induced (1 μM) recruitment of GRK2-rLuc8 or **g** GRK3-rLuc8 to rGFP-CAAX in HEK293 cells expressing ACKR4, and **f**, **h** corresponding basal interaction (BRET ratio) before chemokine addition; *n* = 3, mean ± SD. **i** Co-immunoprecipitation of FLAG-GRK2 and Gγ-cpVenus (of the heterotrimeric G protein) with HA-ACKR4 in transiently transfected HEK293 cells in the absence of chemokine stimulation; *n* = 3, representative replicate. Statistical analysis: **a**, **d**–**h** unpaired two-sided *t* test, **b** ordinary two-way ANOVA, **c** ordinary one-way ANOVA; exact *P* values are shown in the graphs.

As the experiments described above and elsewhere^35^ showing that GRK2 interacts with the heterotrimeric, inactive G protein, challenge the current model, which postulates that G protein activation is a prerequisite for GRK2/3 recruitment^10,41^, we set out a series of additional experiments to substantiate our discovery. We first investigated the association of GRK2 with the G protein by BRET. To this end, we co-expressed nLuc-tagged GRK2, all three subunits of the G protein (Gα_i1_/Gβ/Gγ-fused to cpVenus), together with the native receptor. An enhanced BRET signal was observed between nLuc-GRK2 and Gγ-cpVenus when cells expressing the cognate receptors, ACKR4 or CCR7, were stimulated with CCL19, but not in cells expressing the non-cognate receptor ACKR3 (Fig. 6a, Supplementary Fig. 4a). In contrast, no receptor-triggered association was detected between the GRK2 R587Q mutant and Gγ-cpVenus (Fig. 6a). Notably, the basal interaction between GRK2 and Gγ of the heterotrimeric G protein in the absence of ligand was higher in cells expressing ACKR4 than in cells expressing CCR7 (Fig. 6b). Furthermore, the basal interaction of GRK2 R587Q with Gγ was significantly reduced for both receptors (Fig. 6b). Similar results were obtained for GRK3 (Fig. 6c, d, Supplementary Fig. 4b). Although these experiments do not allow the discrimination between the inactive, heterotrimeric G protein and the active, free Gβγ protein, these data support the concept that GRK2/3 associate with the G protein, and that this interaction is further enhanced by ACKR4 stimulation with CCL19. Next, we tested whether overexpressing the heterotrimeric G protein would enhance the association of GRK2/3 with the plasma membrane. Of note, G protein overexpression augmented both the basal interaction and CCL19-stimulated association of GRK2-rLuc8 and GRK3-rLuc8 with rGFP-CAAX at the plasma membrane of ACKR4-expressing cells (Fig. 6e–h, Supplementary Fig. 4c, d). Finally, to test whether ACKR4 forms a ternary complex with GRK2 and the heterotrimeric G protein, we performed co-immunoprecipitation experiments in cells expressing HA-tagged ACKR4, FLAG-tagged GRK2 and the trimeric G protein (Gα_i1_/Gβ/Gγ-tagged with cpVenus). As shown in Fig. 6i, co-immunoprecipitated FLAG-tagged GRK2 and cpVenus-tagged Gγ were detected in HA-ACKR4 pulldowns. This confirms that, in its ligand-free state, ACKR4, together with GRK2 and the G protein, at least the γ-subunit, form a ternary complex.

### Chemokine scavenging by ACKR4 mainly depends on the recruitment of GRK2/3

**Fig. 7:**
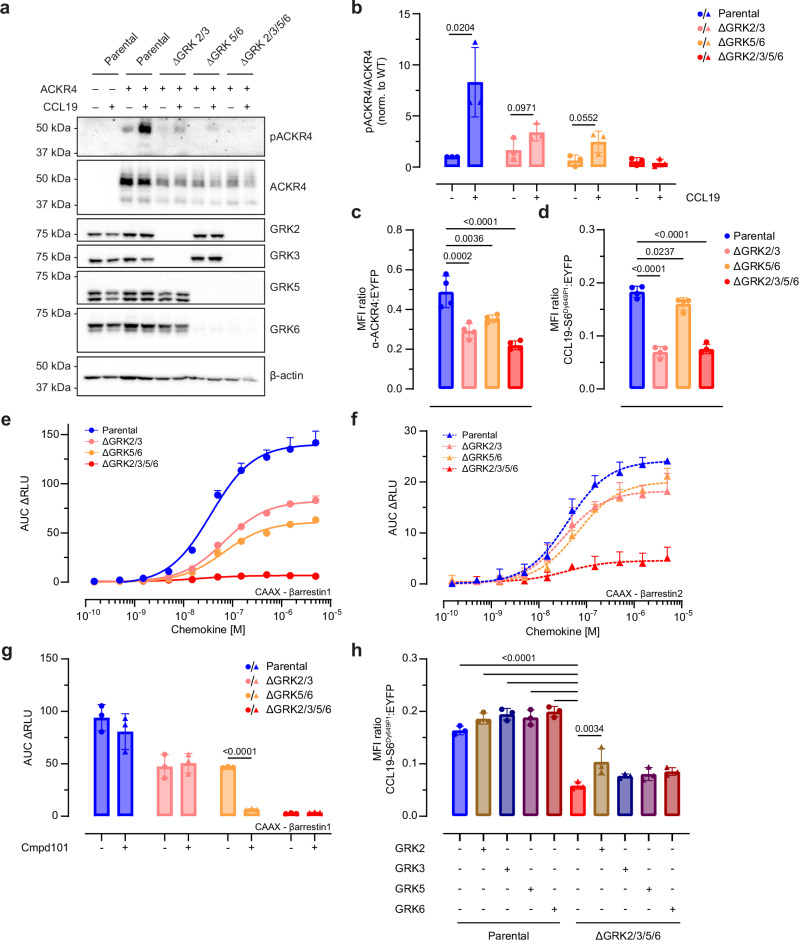
GRKs conjointly coordinate steady-state and chemokine-driven receptor phosphorylation, trafficking and CCL19 internalisation. **a** Representative Western blot of HEK293 parental and GRK knockout (ΔGRK2/3, ΔGRK5/6, ΔGRK2/3/5/6) cells transiently transfected with ACKR4 and stimulated with 25 nM CCL19 or PBS for 10 min. **b** Quantitative Western blot analysis of HEK293 parental and GRK knockout cells of ACKR4 phosphorylation at Ser338/Thr342 in the absence or presence of 25 nM CCL19, normalised to WT steady-state conditions; *n* = 3, mean ± SD. **c** Receptor surface staining normalised to co-expressed EYFP (MFI ratio) of parental and GRK knockout cells; *n* = 4, mean ± SD. **d** Chemokine internalisation divided by EYFP expression (MFI ratio) of parental and GRK knockout cells upon treatment with 5 nM fluorescently labelled CCL19 for 30 min; *n* = 4, mean ± SD. **e** Bystander smBiT-βarrestin1 and **f** smBiT-βarrestin2 recruitment to lgBiT-CAAX in response to ACKR4 stimulation with CCL19 in transiently transfected HEK293 parental and GRK knockout cells; *n* = 3, mean ± SD. **g** Bystander smBiT-βarrestin1 recruitment to lgBiT-CAAX in ACKR4 expressing HEK 293 parental and GRK knockout cells upon addition of 1 μM CCL19 in the presence of 10 μM cmpd101 or DMSO as vehicle control; *n* = 3, mean ± SD. **h** CCL19-S6^Dy649P1^ internalisation (5 nM over 30 min) of parental and ΔGRK2/3/5/6 cells transiently expressing ACKR4 and reconstituted with GRK2, GRK3, GRK5 or GRK6; *n* = 3, mean ± SD. Statistical analysis: **b**, **g** unpaired two-sided *t* test, **c d** ordinary one-way ANOVA, **h** ordinary two-way ANOVA; exact *P* values are shown in the graphs.

To better understand the roles of the different GRKs, and to overcome the limitations of the bystander BRET approach regarding GRK5/6, we next used HEK293 cells lacking either GRK2 and GRK3 (ΔGRK2/3), GRK5 and GRK6 (ΔGRK5/6), or all four GRKs (ΔGRK2/3/5/6)^12^. To gain insights into receptor phosphorylation, we first exploited the commercially available pS338/pT342-ACKR4 antibody specifically detecting phosphorylated Ser338 and Thr342 in the tail of ACKR4. ACKR4 in its apo state showed basal S338/T342-phosphorylation, which was significantly increased upon stimulation with CCL19 (Fig. 7a, b). CCL19-driven ACKR4 phosphorylation was profoundly reduced but still present in ΔGRK2/3 and ΔGRK5/6 cells (Fig. 7a, b). No ACKR4 phosphorylation was detected in ΔGRK2/3/5/6 cells (Fig. 7a, b). ACKR4 was expressed at the surface of all three cell lines lacking different GRKs, but did not reach levels observed in parental, GRK proficient cells (Fig. 7c). Importantly, uptake of fluorescent CCL19-S6^Dy649P1^ was profoundly reduced in ΔGRK2/3 cells and not further impaired in ΔGRK2/3/5/6 cells (Fig. 7d). In marked contrast, CCL19-S6^Dy649P1^ uptake was only slightly, but significantly reduced in ΔGRK5/6 cells as compared to parental cells (Fig. 7d). Interestingly, CCL19-driven recruitment of βarrestin1 to receptor proximal CAAX was reduced by roughly 50%, whereas βarrestin2 recruitment was only marginally decreased in cells lacking either GRK2/3 or GRK5/6 (Fig. 7e, f). βarrestin recruitment was abrogated in cells lacking all four GRKs. This prompted us to exploit the GRK2/3 inhibitor cmpd101. As expected, treating ΔGRK2/3 or ΔGRK2/3/5/6 cells with cmpd101 did not affect CCL19-mediated βarrestin1 recruitment (Fig. 7g). Remarkably, cmpd101 treatment only slightly reduced βarrestin1 recruitment in parental cells, but completely blocked βarrestin1 recruitment in ΔGRK5/6 cells (Fig. 7g). Similar results were obtained for βarrestin2 recruitment (Supplementary Fig. 5a). Assessing ligand-independent receptor internalisation revealed that ACKR4 in its apo state showed a non-significant tendency to internalise more in ΔGRK2/3, and slightly less in ΔGRK2/3/5/6 cells (Supplementary Fig. 5b). Attempts to reconstitute single GRKs in ΔGRK2/3/5/6 cells appeared very challenging, as transfecting parental and ΔGRK2/3/5/6 cells with even small amounts of any GRK plasmid (48-times less GRK than receptor) substantially boosted the uptake of fluorescent CCL19-S6^Dy649P1^ as exemplified for GRK3 (Supplementary Fig. 5c). Notably, under the same experimental condition, expressing any GRK in parental cells did not enhance uptake of fluorescent CXCL12-S6^Dy649P1^ by its receptor ACKR3 (Supplementary Fig. 5 d). At a 1:1000 ratio (GRK3:ACKR4) we accomplished to re-express GRK3 in ΔGRK2/3/5/6 cells a level comparable to parental cells (Supplementary Fig. 5e). Under these conditions, only re-expressing GRK2 in ΔGRK2/3/5/6 cells was able to elevate CCL19-S6^Dy649P1^ uptake (Fig. 7h). Collectively, these data reveal an interplay of different GRKs in ACKR4 phosphorylation and βarrestin recruitment, and an involvement of GRK2/3 in chemokine uptake.

### Identification of Ser349 phosphorylation by GRK5/6 under steady-state conditions and by GRK2/3 upon CCL19 stimulation

**Fig. 8:**
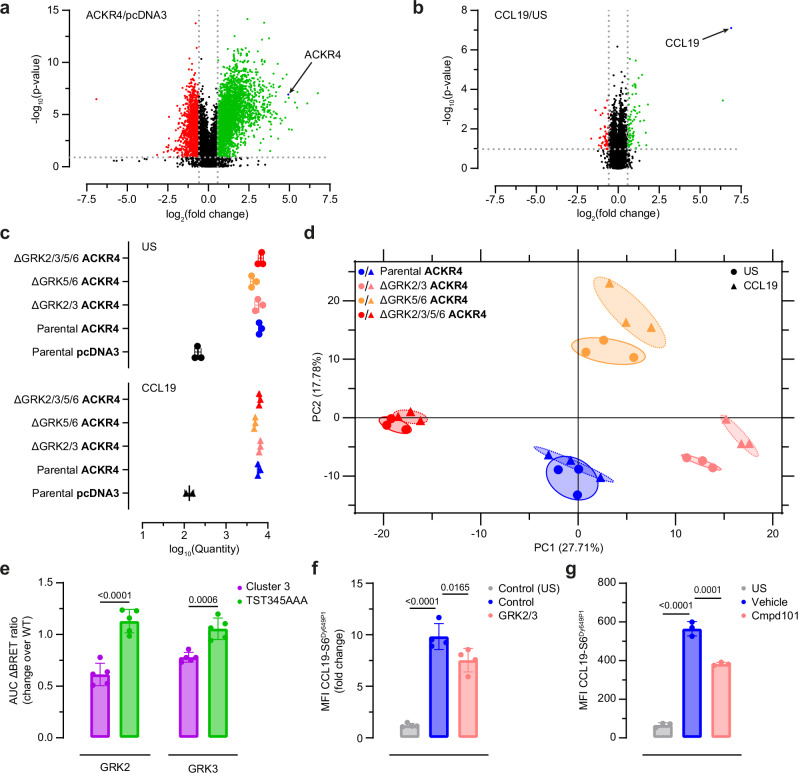
Mass spectrometry analysis reveals different roles of GRKs in protein abundance of the ACKR4 interactome and identifies receptor phosphorylation at Ser349. **a** Comparison of the protein abundance in parental cells expressing pcDNA3 or HA-ACKR4, and **b** parental cells expressing HA-ACKR4 stimulated with 100 nM CCL19 or buffer (unstimulated, US); dotted lines indicate cutoff values. **c** Quantities of ACKR4 in the different genotypes presented as log_10_. **d** Principal component analysis (PCA) of the protein abundance in the four cell lines after CCL19 stimulation or under steady-state conditions. **e** GRK2/3 recruitment to ACKR4 upon ligand stimulation (1 μM CCL19) measured in a bystander BRET assay; *n* = 5, mean ± SD. **f** CCL19-S6^Dy649P1^ uptake (25 nM for 30 min) by endogenous ACKR4 in human BJ hTERT fibroblasts transiently transfected with either control siRNA or siRNA against GRK2 and GRK3; fold change over unstimulated (US) per condition, *n* = 4, mean ± SD. **g** Uptake of fluorescently labelled CCL19 (25 nM for 30 min) by ACKR4 in human BJ hTERT fibroblasts treated with 50 μM of the GRK2/3 inhibitor cmpd101, *n* = 3, mean ± SD. **a**–**d** Data of technical triplicates or duplicates (parental pcDNA3 + CCL19). Statistical analysis: **a**, **b** unpaired two-sided *t* test with unequal variance and without* P* value adjustment, **e** unpaired two-sided *t* test, **f**,** g** ordinary one-way ANOVA; exact *P* values are shown in the graphs.

So far, our understanding of ACKR4 phosphorylation is limited by the availability of solely one antibody recognising the S338/T342 phospho-site. To gain a more comprehensive overview, we undertook a mass spectrometry approach. To establish the system, we first transfected HEK293 cells with HA-tagged ACKR4 to enable receptor pull-downs. Mass spectrometric analysis revealed an enrichment for ACKR4 peptides in receptor pull-downs as compared to control pull-downs (Fig. 8a). Moreover, proteins of pathways involved in cellular localisation, transport and endocytosis, such as caveolin1, caveolin2, dynamin1 and dynamin2 were more frequently found in the ACKR4 pull-down samples (Supplementary Fig. 6a, b). Stimulation of cells with CCL19 led to a significant enhancement of CCL19 in the ACKR4 pull-downs, but chemokine treatment did not markedly change the levels of receptor-associated proteins (Fig. 8b). Next, we used HA-ACKR4-transfected parental, ΔGRK2/3, ΔGRK5/6 and ΔGRK2/3/5/6 HEK293 cells. The abundance of identified ACKR4 was comparable between the different cell lines and the replicates (Fig. 8c). Principal component analysis (PCA) was applied to compare the differences in protein abundance among the four genotypes, and CCL19-stimulated versus unstimulated cells. The PCA revealed that the protein associations from different genotypes cluster mainly according to principal component 1, irrespective of CCL19 stimulation. Notably, in ΔGRK2/3 and ΔGRK5/6 cells, ACKR4-associated proteins segregated between CCL19-stimulated and non-stimulated conditions (Fig. 8d) in principal component 2, less so for parental and ΔGRK2/3/5/6 cells. We detected several tryptic peptides covering parts of the C-terminus of ACKR4 and identified two serine residues (Ser338, Ser349) and one threonine (Thr342) residue to be phosphorylated (Supplementary Fig. 7). Consistent with the antibody-based evidence, we found phosphorylation at either Ser338 or Thr342, but not in combination. Moreover, we identified S349 as an additional phosphorylation site. In-depth PTM analysis of all cell lines revealed that phosphorylation of Ser349 was significantly upregulated under steady-state conditions in parental, GRK proficient cells relative to ΔGRK5/6 and ΔGRK2/3/5/6 cells but not compared to ΔGRK2/3 cells (Supplementary Fig. 6c, d). Moreover, CCL19 stimulation resulted in a significant enhancement of pSer349 in cells lacking GRK5/6, but not in the other cell lines (Supplementary Fig. 7). Therefore, we re-introduced Ser349 in the ACKR4 Cluster 3 mutant and observed a significant increase in the recruitment of GRK2 and GRK3 upon CCL19 stimulation (Fig. 8e), corroborating that Ser349 of ACKR4 is phosphorylated by GRK2/3 in response to CCL19 stimulation.

Finally, we used human BJ hTERT fibroblasts, which endogenously express ACKR4, to confirm the role of GRK2/3 in chemokine internalisation in a physiologically relevant cellular system. Human BJ hTERT fibroblasts readily expressed ACKR4 on the cell surface and internalised fluorescently labelled CCL19-S6^Dy649P1^ (Supplementary Fig. 8a, b). CCL19-S6^Dy649P1^ uptake was significantly reduced in human BJ hTERT fibroblasts upon siRNA-mediated knockdown of GRK2/3 as compared to cells transfected with control siRNA (Fig. 8f, Supplementary Fig. 8c). CCL19-S6^Dy649P1^ uptake was also substantially reduced in human BJ hTERT fibroblasts upon inhibition of GRK2/3 with cmpd101 (Fig. 8g), corroborating the critical role of GRK2/3 in ACKR4 function.

In summary, our study provides evidence that ACKR4 in its apo state constitutively pre-associates with βarrestins and cycles between the plasma membrane and endosomal compartments. While reaching the cell surface, ACKR4 is able to efficiently sense and co-internalise CCL19. We demonstrated that serine/threonine residues in the tail region of ACKR4 are essential for the enhanced recruitment of βarrestins by CCL19, and consequently for efficient chemokine uptake. Moreover, we showed that different serine/threonine clusters in the tail of ACKR4 are involved in steady-state and CCL19-driven association of all four non-visual GRKs. In addition, we identified two serine and one threonine residues to be differentially phosphorylated by GRKs. Furthermore, we provide evidence that the recruitment of GRK2 and GRK3 to ACKR4 occurs independently of G protein activation. Finally, we discovered that ACKR4 in its apo state can form a ternary complex with GRK2 and the heterotrimeric G protein without the need for its activation, and that GRK2, in combination with further GPCR kinases, plays an important role in βarrestin recruitment and CCL19 internalisation.

## Discussion

Chemokine gradient formation by ACKRs is essential for guided cell migration in health and disease. In contrast to classical chemokine receptors, which utilise G_i_ protein-dependent signalling leading to cell migration, ACKRs are completely biased towards βarrestins for chemokine scavenging and do not couple to G proteins. In fact, ACKR2-5 constitutively associate with βarrestins to various degrees and cycle between the plasma membrane and endosomes, even in their apo state, and chemokine binding often enhances the interaction with βarrestins^24–26^. ACKR4 is best known for its scavenging activity of the CCR7 ligands, CCL19 and CCL21, which coordinate the migration and homing of dendritic cells and T cells to launch adaptive immune responses against invading pathogens. The current knowledge on molecular mechanisms of how ACKR4 scavenges its ligands is limited and has mainly been investigated using nanoBiT and BRET sensors that rely on C-terminally tagged receptors^33–35^. Importantly, we recently identified that the integrity of the C-terminal tip of ACKR4 is critical for its function and that C-terminal tagging impaired ligand-mediated βarrestin recruitment and enhanced its ability to scavenge chemokines^39^. Therefore, we established an indirect, bystander split-luciferase assay to demonstrate that βarrestins pre-associate with the plasma membrane of cells expressing untagged ACKR4 and that CCL19 stimulation recruited additional βarrestins, resulting in efficient chemokine internalisation.

In the present study, we also demonstrate that the GPCR kinases GRK2 and GRK3 are actively recruited to untagged ACKR4 upon stimulation with CCL19, which is in line with previous studies using tagged receptor^35^. In addition and against the paradigm^6,10^, we provide evidence that GRK2 and GRK3 interact with ACKR4 without prior G protein coupling and activation. We also show that these two kinases substantially contribute to the chemokine scavenging function of ACKR4. Importantly, cells lacking GRKs failed to recruit βarrestins towards chemokine-stimulated ACKR4 and consequently were inefficient in CCL19 internalisation. Expressing GRK2 in ΔGRK2/3/5/6 cells partially restored the chemokine internalisation capacity of ACKR4. Moreover, we found pre-association of different GRKs with specific regions of ACKR4’s C-terminal tail in the absence of ligands and that proximal serine and threonine residues are involved in steady-state trafficking (Cluster 1), whereas the terminal residues are necessary for βarrestin recruitment and chemokine scavenging (Cluster 3). Furthermore, we identified Ser349 to be phosphorylated by GRK5/6 under steady-state conditions and by GRK2/3 after ligand stimulation. Together with Ser338/Thr342 we identify that three distinct residues in the C-terminus of ACKR4 are phosphorylated by the GRKs.

Another atypical chemokine receptor, namely ACKR3, was also shown to recruit GRKs upon CXCL12 stimulation^36^. In fact, GRK2 and GRK5 were shown to phosphorylate residues in the C-terminus of ACKR3, whereas GRK3 and GRK6 showed no activity^36^. For ACKR3, GRK2 was found to phosphorylate preferentially distal Ser/Thr residues in the C-terminus, facilitating persistent interaction with βarrestins, while GRK5 mainly phosphorylated proximal residues, although some residues were shared targets of both kinases^38^. However, inhibition of GRK2 only delayed chemokine internalisation without affecting the chemokine scavenging activity of ACKR3. Of note, CXCL12-mediated recruitment of GRK2 to ACKR3 was abrogated in the ACKR3 ST/A mutant or a mutant lacking the entire C-terminus, referred to as ACKR3 deltaC^36^. These observations align with the idea that GRKs could potentially have tail and core interactions with receptors analogous to those of βarrestins^4^. Moreover, the kinase-dead GRK2 mutant, GRK2 K220R, was not recruited to ligand-stimulated ACKR3^36^. Here, we have shown that both basal interaction as well as ligand-mediated recruitment of GRK2 and GRK3 were substantially reduced for the ACKR4 ST/A mutant. Similarly, the corresponding kinase-dead mutants showed reduced steady-state interaction and ligand-induced recruitment to ACKR4. Interestingly, genetic targeting of GRK2/3 profoundly impaired chemokine uptake by ACKR4, which contrasts with ACKR2 and ACKR3. The latter two receptors were analysed in the same cellular knockout system and showed normal βarrestin recruitment in cells lacking GRK2/3, but altered recruitment in cells lacking GRK5/6^37,44^. Additionally, the necessity of βarrestins in ACKR3-mediated CXCL12 scavenging was challenged by observations made in interneurons from βarrestin1/2^−/−^ mouse embryos^36^. Although knocking in ACKR3 ST/A rescued the embryonically lethal phenotype of Ackr3^−/−^ mice, ACKR3 ST/A expressing cells could only undergo ligand-independent receptor internalisation, but failed to scavenge CXCL12^36^. In the case of ACKR4, we also observed continuous receptor cycling in the ST/A mutant, which was insufficient for effective CCL19 scavenging. However, while βarrestins were crucial for chemokine uptake by native ACKR4^39^, βarrestins were dispensable for chemokine scavenging by ACKR3^36^. Collectively, these findings may suggest the existence of individual control mechanisms for chemokine scavenging within the ACKR family.

Furthermore, ectopic expression of a single GRK substantially enhanced chemokine internalisation by ACKR4 both, in parental as well as in ΔGRK2/3/5/6 cells, exceeding the ligand uptake capacity of ACKR4, supporting an important role of GRKs for scavenging. Notably, GRKs are essential for chemokine-induced recruitment of βarrestins to ACKR4, as this activity was completely abrogated in ΔGRK2/3/5/6 cells. As βarrestin recruitment and CCL19 uptake were both affected in either ΔGRK2/3 cells or ΔGRK5/6 cells, it is tempting to speculate, that all four GRKs collectively regulate ACKR4 trafficking and that the localisation of the GRKs might be important. GRK5/6 are prone to be present at the plasma membrane facilitating the phosphorylation of receptors in their apo state, as well as in response to ligand binding^40^. GRK2/3 are actively recruited to the receptor upon agonist stimulation favouring the phosphorylation after receptor activation^40^. Along this line, our results show that GRK5/6 indeed phosphorylate Ser349 in the tail of ACKR4 in the absence of ligands, whereas GRK2/3 enhance ACKR4 phosphorylation upon chemokine stimulation. Furthermore, our results indicate that GRK2/3 facilitate ligand-induced receptor phosphorylation and chemokine internalisation, whereas GRK5/6 mainly seem to regulate steady-state phosphorylation and trafficking of ACKR4. The importance of GRK2/3 in CCL19 uptake was corroborated in human fibroblasts expressing endogenous ACKR4, as either their inhibition by cmpd101 or their knockdown by siRNA substantially reduced chemokine internalisation.

GRK2 and GRK3 recruitment and their kinase activity are thought to rely on previous activation of heterotrimeric G proteins, i.e. the liberation of free Gβγ, which serve as interaction sites for the PH domain of GRK2/3 enabling their recruitment to the plasma membrane to phosphorylate the GPCR^40^. Interestingly, a new classification of GPCRs into three groups has recently been proposed based on GRK involvements^45^. One proposed group of GPCRs is regulated solely by GRK2/3 and necessitates preceding G protein activation for membrane association of GRK2/3 via the interaction with Gβγ. Another group of GPCRs includes receptors that are phosphorylated only by membrane-tethered GRK5/6, possess an intrinsic βarrestin bias, and do not activate G proteins. The third group of GPCRs is regulated by all four GRKs and involves G protein dependent and independent signalling^45^. According to this proposed classification, ACKR4 would belong to the third group. However, ACKR4 is not able to bind or signal through G proteins but still recruits GRK2 and GRK3. Interestingly, the recruitment of GRK2 to ACKR3 was explained by the presence of CXCR4 in the same cell and the ability of both receptors to form heterodimers, thereby targeting GRK2 to CXCR4-bound ACKR3, because both receptors are activated by CXCL12^37,46^. While this scenario is plausible for the ACKR3/CXCR4 axis, it cannot be a core mechanism common to the ACKR family, as we are not aware of any cell that would simultaneously express the classical (CCR7) and the atypical (ACKR4) receptor for CCL19. Consequently, activation of G proteins through a classical receptor cannot be the cause of the active GRK2/3 recruitment to ACKR4. Mutation of GRK2, D110A to inhibit Gα_q_ interaction and R587Q to inhibit Gβγ interaction, showed that the recruitment to ACKR4 indeed depends on the interaction of GRK2 with inactive, heterotrimeric G protein. This was further substantiated by our proximity-based BRET experiments, which revealed an association of GRK2/3 with the γ-subunit of the heterotrimeric G protein upon ligand binding to ACKR4, as well as a more pronounced recruitment of GRK2/3 towards ACKR4 by artificially increasing the G protein abundance. Moreover, we demonstrate that an atypical chemokine receptor in its apo state can form a ternary complex with GRK2 and components of the heterotrimeric G protein, notably without its prior activation. Thus, it is tempting to speculate that the inactive heterotrimeric G protein might serve as scaffold for GRKs in the context of βarrestin-biased receptors.

Cryo-EM analysis of certain phosphorylated peptides of GPCRs with βarrestins revealed a conserved motif (“P-X-P-P”) enabling a stabilised binding of βarrestins to the peptides. Notably, such a PXPP-motif is missing in ACKR3, but the receptor is still able to interact with βarrestins^47^. In contrast, ACKR4 owns a cluster of serine and threonine residues (Cluster 3) that are possibly phosphorylated at the C-terminal tip, and this “T-S-T-F-S” motif might fulfil a similar role as the PXPP motif. In fact, mutation of these residues led to a loss of βarrestin recruitment to ACKR4 upon stimulation with CCL19 supporting a stabilising role of this motif in ACKR4 interaction with βarrestins. Indeed, we identified by mass spectrometry Ser349 within this motif of Cluster 3 to be phosphorylated. Together with the proximity-based interaction analysis, our data suggests that pSer349 together with the other Ser/Thr residues of Cluster 3 seems to be involved in stabilising the pre-association of ACKR4 with βarrestins, in recruiting further βarrestins upon ligand binding, and in regulating chemokine scavenging. We also identified Ser338 and Thr342 of ACKR4 to be phosphorylated. Although mutation of these two residues only moderately affected CCL19-driven βarrestin and GRK3 recruitment, it remains possible that these phospho-sites contribute to a stable interaction between receptor and βarrestins in conjunction with the terminal phospho-sites as the Cluster 3 mutant recruits more βarrestins than ACKR4 ST/A. Notably, Ser338 and Thr342 phosphorylation was independently identified by mass spectrometry and by utilising an antibody raised against a synthetic phospho-peptide derived from human ACKR4 around the phosphorylation site of Ser338/Thr342. Although the Western blot analysis may suggests a simultaneous phosphorylation at both sites, our mass spectrometry analysis revealed that both sites can be phosphorylated individually, but not in combination. Collectively, our study reveals a heterogenous phosphorylation pattern of ACKR4 involving at least three different phosphorylation sites that contribute to control receptor functions. Our mass spectrometry analysis of ACKR4 further revealed significant changes only for Ser349 among the different GRK KO cells. In addition, no changes upon stimulation were detected in parental cells. Because only a minor fraction of ACKR4 resides at the plasma membrane and the majority of receptor is found in endosomal compartments^32^, the approach using a pull-down assay to bind phosphorylated and non-phosphorylated receptors might not be ideal to identify multiple phosphorylation sites. The majority of receptors resemble the phosphorylation state of non-stimulated receptors and hence, minor changes due to stimulation might not be detectable. Using purified receptor and GRKs to map the phosphorylation introduced by the different GRKs will help to unravel the barcode of ACKR4 in future studies.

However, the identification of Ser349 and Ser338/Thr342 being target of phosphorylation by GRKs uncovers residues of a phosphorylation barcode in the tail of ACKR4 that broadens the understanding of the molecular processes underlying the scavenging function of ACKR4 and prepares for following future investigations.

## Methods

### Plasmids

Plasmids were generated by PCR using the primer pairs listed in Supplementary Tables 1–3. Amplified DNA was digested with restriction enzymes (ThermoFisher, Waltham, MA, USA) and cloned into pcDNA3 or pIRES vectors using the T4 ligase (ThermoFisher). The rGFP insert from pcDNA3 rGFP-FYVE^48^ was exchanged for lgBiT to generate pcDNA3 lgBiT-FYVE. Furthermore, pcDNA3 CCR7-rluc8^49^ served as the backbone for the insertion of the different GRKs N-terminal of rLuc8. pIRES EYFP_ACKR4^39^ was used to insert the various receptors after the IRES sequence to express EYFP as transfection control for the respective receptors. The different Gβγ (upstream of the IRES sequence) and Gα (downstream of the IRES sequence) pairings were exchanged in the pIRES Gβ1-T2A-cpV-Gγ2_Gαi1^35^ expression vector.

Site-directed mutagenesis served to generate the ACKR4 Cluster 1, Cluster 2, and Cluster 3 mutants. The corresponding primers used for site-directed mutagenesis are listed in Supplementary Table 4. The ACKR4 ST/A mutant was generated by the combination of all three clusters. Single- and double point mutations in GRK expression plasmids were introduced by site-directed mutagenesis.

### Cell culture and transient transfection

HEK293, HEK293A parental (GRK), HEK293A ΔGRK2/3, HEK293A ΔGRK5/6, HEK293A ΔGRK2/3/5/6, HEK293A parental (G protein) and HEK293A ΔGα_q/12/s_ were cultured at 37 °C, 5% CO_2_ and 95% humidity in DMEM (Capricorn Scientific GmbH, Ebsdorfergrund, Germany) supplemented with 10% fetal calf serum (FCS; Lonza, Basel, Switzerland) and 1% penicillin/streptomycin (P/S; Pan Biotech, Aidenbach, Switzerland). Human BJ hTERT fibroblasts were cultured in DMEM supplemented with 20 %FCS and 1 % P/S. The CRISPR KO cells (ΔGRK2/3, ΔGRK5/6, ΔGRK2/3/5/6^12^, and ΔGα_q/12/s_^42^) and respective parental cell lines were kindly provided by Asuka Inoue (Tohoku University, Japan) under an MTA.

All cell lines were transiently transfected with the Neon Transfection System (ThermoFisher) according to the manufacturers’ protocol. Briefly, 5 × 10^5^ HEK293 cells were mixed with 5 μg of DNA and electroporated using 2 pulses with 1100 V and a width of 20 ms. 5 × 10^5^ human BJ hTERT fibroblasts were mixed with 200 nM respective siRNAs and electroporated using 1 pulse with 1650 V and a width of 20 ms. HEK293 cells were cultured for 20–24 h, whereas BJ hTERT fibroblasts were cultured for 44–48 h post transfection in DMEM supplemented with 20 % FCS prior analyses.

For co-transfection, the following plasmid ratios were used to reach a total of 5 μg DNA: in bystander βarrestin recruitment split-luciferase assays ACKR4:lgBiT-CAAX or lgBiT-FYVE:smBiT-βarrestins (5:2:1), in bystander GRK recruitment BRET assays ACKR4:rGFP-CAAX:GRKs-rLuc8 (15:8:1), in G protein activation (BRET) assays ACKR4, CCR7 or pcDNA3 (empty vector):G protein sensors (1:3), in co-immunoprecipitation experiments HA-ACKR4:3xFLAG-GRK2:Gβγ-cpVenus/Gα_i1_ (8.5:1:2), in bystander GRK-G protein interaction BRET assays ACKR4, CCR7 or ACKR3:GRK2 or GRK3:Gβγ-cpVenus/Gα_i1_ (50:1:10) and in bystander GRK recruitment BRET assays (with G protein overexpression) ACKR4:rGFP-CAAX:GRKs-rLuc8:pcDNA3 (empty vector) or Gβγ-cpVenus/Gα_i1_ (15:8:1:4) were transfected. In GRK reconstitution experiments (chemokine uptake or western blot analysis), ratios between GRKs and ACKR4 ranged from 1:48 up to 1:1000; for uptake experiments with ACKR3, the ratio was only 1:48.

For transient knockdown in human BJ hTERT fibroblasts, the following siRNAs were used: control siRNA – Qiagen, #1027281; GRK2 – Qiagen, #1027417 (#SI00287378), GRK3 – Qiagen, #1027417 (#SI02223865).

### Chemokine production

All chemokines used in this study, namely hCCL19, hCCL19-S6 and hCXCL12-S6, were expressed in *E. coli* BL21(DE3), purified and site-specifically labelled with Dy649P1 (Dyomics GmbH, Jena, Germany) following an established protocol^50^. In brief, proteins were extracted from bacterial inclusion bodies, enriched by affinity chromatography utilising the N-terminal His-SUMO-tag that was subsequently removed after chemokine refolding and Ulp1 protease digestion. As further purification steps, a cation exchange chromatography followed by reverse phase HPLC were performed. Finally, S6-tagged proteins were site-specifically labelled using 1 μM SFP synthase (New England Biolabs, Ipswich, MA, USA) and 15 μM CoA-Dy649P1 in 50 mM Hepes pH 7.6, 10 mM MgCl_2_, 100 mM NaCl and 20% glycerol at 37 °C for 2 h, and eventually purified by reverse-phase HPLC.

### Split-luciferase (NanoBiT) assay

Transfected cells were detached, resuspended in PBS complemented with 5 mM glucose (PBS-G) and transferred in technical duplicates into a white 96-well half-well plate (PerkinElmer, Waltham, MA, USA). Next, 5 μM coelenterazine H (Biosynth, Staad, Switzerland) was added to the cells and incubated at 37 °C for 5 min prior to the measurement on a Tecan Spark 10 M multiplate reader (Tecan, Mändorf, Switzerland). Subsequently, bioluminescence was measured every 30 s (385–440 nm, 350 ms integration time) and, after 5 min, cells were stimulated with chemokines or PBS-G as a negative control. The change in bioluminescence was monitored for further 15 min. For data analysis, signals from ligand-stimulated cells were divided by signals from the negative control (ΔRLU), and subsequently, the area under the curve (AUC) was determined for quantification as illustrated in Supplementary Fig. 1b. In experiments including GRK2/3 inhibitor treatment, cells were pre-treated with 10 μM compound 101 (cmpd101; LubioScience GmbH, Zürich, Switzerland) for 30 min and the inhibitor was also present during the measurement. For some experiments, cells were pre-treated with 200 μg/ml pertussis toxin (PTX; Enzo Life Sciences AG, Lausen, Switzerland) for 4 h to inhibit G_i_ protein activation.

### Flow cytometry

Transiently transfected HEK293 cells were stained on ice with a primary antibody against ACKR4 (Biolegend; #362102, dilution 1:750) for 30 min, washed and incubated with a secondary anti-mouse antibody coupled to Alexa Fluor 647 (ThermoFisher; #A-21235, dilution 1:1000) for 20 min. For ligand-independent receptor internalisation studies, the receptor staining protocol was adopted to guarantee staining of all molecules present at the cell surface. Here, incubation times on ice were extended to 60 min for primary antibody staining and to 45 min for secondary antibody staining. Where indicated, some of the samples were incubated at 37 °C for 30 min to allow receptor internalisation in between the staining procedure, whereas the other samples remained on ice for the same period. For quantification, the ratio between the cells with spontaneous receptor internalisation and the cells kept on ice was calculated to obtain the percentage of internalised receptors. Surface-expressed CCR7 was stained with an APC-coupled anti-CCR7 antibody (ThermoFisher; #17-1979-42, dilution 1:50).

For chemokine uptake studies, cells were incubated with 5 nM fluorescent hCCL19-S6^Dy649P1^ for 30 min if not stated differently. During the stimulation, cells were kept in a Hepes-buffered solution containing 10 mM Hepes, 145 mM NaCl, 5 mM KCl, 1 mM MgCl_2_, 1 mM CaCl_2_, 1 mM Na_2_HPO_4_ and 5 mM glucose.

Measurement of cell-associated fluorescence (MFI) was performed on a BD LSRFortessa flow cytometer using the BD FACSDiva^TM^ software v9.0.1 (BD Biosciences, San Jose, CA, USA) and analysed with the FlowJo v10.8.1 software (BD Biosciences). Background fluorescence was subtracted from signals, and the common gating strategy is depicted in Supplementary Fig. 9.

### Bioluminescence resonance energy transfer (BRET) assay

HEK293 cells were resuspended in PBS-G and distributed in technical duplicates into a 96-well half-well plate (PerkinElmer). Then, 5 μM coelenterazine H (Biosynth) was added, and the bioluminescence and associated fluorescence were measured on a Tecan Spark 10 M multiplate reader (Tecan). For bystander GRK recruitment, cells were incubated at 37 °C for 10 min, followed by baseline measurement for 5 min and stimulation with chemokines or PBS-G as a negative control. Changes in rGFP fluorescence (490–560 nm, 350 ms integration time) and bioluminescence (385–440 nm, 350 ms integration time) were measured for further 15 min. Baseline cpVenus fluorescence (505–590 nm, 350 ms integration time) and bioluminescence (385–440 nm, 350 ms integration time) in G protein dissociation experiments were monitored for 3 min after an incubation at 37 °C for 5 min. After chemokine stimulation or addition of PBS-G, changes in signals were recorded for an additional 5 min.

For quantification, BRET signals of chemokine-stimulated cells were divided by signals of the negative control (ΔBRET ratio), followed by determination of the AUC.

### Co-immunoprecipitation and Western blot analysis

For Western blot analysis, transiently transfected cells were lysed in 50 mM Tris pH 8.0, 150 mM NaCl, 1 %Triton-X-100, 0.5 % n-dodecyl-β-D-maltoside, 0.5 mM EDTA and protease inhibitor (Roche, Rotkreuz, Switzerland; #11836170001) at 4 °C for 30 min, followed by lysate clearing (centrifugation at 21,000 × *g*, 20 min, 4 °C), addition of sample buffer and heating of the sample at 37 °C for 10 min. For co-immunoprecipitation, cleared cell lysates were incubated with anti-HA agarose beads (Sigma-Aldrich, #A2095) at 4 °C for 4 h. Immunoprecipitates were washed twice with a high salt buffer (50 mM Tris-HCl pH 8.0, 650 mM NaCl, 5 mM EDTA, 0.5 % Triton-X-100) and twice with a low salt buffer (50 mM Tris-HCl pH 8.0, 150 mM NaCl, 5 mM EDTA, 0.5 % Triton-X-100). Subsequently, the sample buffer was added, and the samples were heated to 37 °C for 10 min. Then, samples were subjected to Western blot analysis and detected using the following antibodies. Primary antibodies: βactin – Abcam, #ab6276 (1:5000); ACKR4 −7TM Antibodies, #7TM0315N (1:1000); pACKR4 − 7TM Antibodies, #7TM0315A (1:1000); GRK2 – Santa Cruz, #sc-13143 (1:500); GRK3 – Cell Signalling, #80362 (1:500); GRK5 – Santa Cruz, #sc-518005 (1:500); GRK6 – Cell Signalling, #5878 (1:1000); GFP – Abcam, #ab32146 (1:10000): FLAG – Sigma-Aldrich, #A8592 (1:3000). Secondary antibodies: goat-anti-mouse – Jackson ImmunoResearch, #115-035-003; goat-anti-rabbit - Jackson ImmunoResearch, #111-035-003 (1:5000).

Images were quantified using the Image Lab software v4.1 (Bio-Rad Laboratories, Hercules, CA, USA).

### Mass spectrometry

7.5 × 10^6^ cells of the four genotypes (HEK293A parental, ΔGRK2/3, ΔGRK5/6 and ΔGRK2/3/5/6) were transiently transfected with HA-ACKR4 (or pcDNA3 empty vector as negative control) in technical triplicates and 24 h later, cells were stimulated with 100 nM CCL19 for 10 min or left untreated in a Hepes-buffered solution (as described above). Cells were lysed and prepared for co-immunoprecipitation as described above. Additionally, after treatment with high and low salt buffers, samples were washed three times with a low salt buffer without detergents (50 mM Tris-HCl pH 8.0, 650 mM NaCl).

For mass spectrometry analysis, 5% 1 M ammonium bicarbonate was added to the samples to reach a basic pH, and the samples were incubated at 60 °C for 10 min. Next, bead digestion was performed with 1 μg trypsin at 37 °C for 1 h (shaking) followed by overnight incubation without shaking. Then, 1% trifluoroacetic acid and iRT peptides (Biognosys) were added. Desalting was performed using UltraMicro Spin Column (The Nest Group) following the manufacturer’s instructions. Subsequently, peptides were dried and resuspended in 1% acetonitrile and 0.1% formic acid in water and stored at −20 °C until mass spectrometric acquisition.

For data-independent acquisition (DIA) LC-MS/MS measurements, 1 μl of the digests per sample was injected into an IonOpticks Aurora series Ultimate CSI 75 µm x 250 mm C18 reversed phase column (AUR3-25075C18-CSI) on a Thermo Scientific™ EASY-nLC™ 1200 nano-liquid chromatography system connected to a Bruker Daltonics timsTOF HT mass spectrometer equipped with a Captive Spray II ion source. LC solvents were A: water with 0.1% FA; B: 80 % acetonitrile, 0.1% FA in water. The nonlinear LC gradient was 1–45 % solvent B for 60 min followed by a column washing step of 90% B for 2.5 min, and a final equilibration step of 1% B for 2.4 min at 60 °C with a flow rate set to a ramp between 600 and 400 nL/min (min 0: 600 nL/min, min 7: 400 nL/min, washing at 600 nL/min). The diaPASEF^®^ method consisted of one full range MS1 scan from 100 to 1700 m/z with an applied ion mobility range from 0.85 to 1.45 1/k0 with ramp and accumulation times set to 100 ms (100 % duty cycle) and 25 PASEF ramps.

DIA mass spectrometric data were analysed using Spectronaut software (version 19.4, Biognosys^51^) with the default settings and the following variable modifications N-terminal acetylation, methionine oxidation and phosphorylation (serine, threonine, tyrosine). Up to 2 missed cleavages were allowed. The initial mass tolerance for the precursor was 4.5 ppm and for the fragment ions was 20 ppm. As a FASTA database, Homo sapiens Uniprot/Swiss-Prot database from 2024-07-01 containing 20435 entries. The minimal peptide length was set to 7 amino acids. The phosphorylation localisation probability is calculated based on a published algorithm^52^. Data sets were filtered before statistical analyses with a site localisation probability for PTMs>75%. Additionally, the data were filtered with a peptide and protein FDR of 1%. Single peptide identification of proteins was not filtered.

Gene ontology (GO) analysis was performed using the online tool ShinyGO 0.81^53^ and the database of biological processes (accessed on 10.01.2025).

### Statistical analysis

Statistical analysis was performed using a two-tailed unpaired t test, ordinary one-way ANOVA or ordinary two-way ANOVA with Dunnett’s multiple comparison test and single-pooled variance. Residuals were analysed for Gaussian distribution using the normality test of D’Agostino and Shapiro-Wilk. The performed statistical analyses are indicated in every figure legend. (GraphPad Prism Software v10.1.2, San Diego, CA. USA).

### Reporting summary

Further information on research design is available in the Nature Portfolio Reporting Summary linked to this article.

## Supplementary information

Supplementary Information

Reporting Summary

Transparent Peer Review file

## Source data

Source Data

## Data Availability

The original data generated in this study have been deposited and are available on Zenodo as entry 18888050 10.5281/zenodo.18888050. The raw MS data, and the quantitative data tables have been deposited to the ProteomeXchange Consortium with the dataset identifier PXD067830 and via the MassIVE partner repository with the dataset identifier MSV000098965. The saved projects from Spectronaut can be viewed with the Spectronaut Viewer (www.biognosys.com/spectronaut-viewer). The following accession code was used in this study: 6U7C 10.2210/pdb6u7c/pdb; structure of human GRK2. Source data, including all uncropped blots, are provided with this paper in a Source Data file. Source data are provided with this paper.
