# Supplementary material for: GPCR kinases shape ACKR4 functions via differential C-terminal phosphorylation
Source: Nat Commun. 2026 May 16;17:6503. doi: 10.1038/s41467-026-73074-4 (PMC13377056; doi:10.1038/s41467-026-73074-4)
Supplement: Supplementary file 1 — Supplementary Information [file 41467_2026_73074_MOESM1_ESM.pdf]

SUPPLEMENTARY INFORMATION

GPCR kinases shape ACKR4 functions via differential C-terminal phosphorylation

Oliver J. Gerken [1,2], Rebecca Warmers [1,2], Clara Hild [1], Niklas Kielkopf [1], Nicola Catone [1], Roland Bruderer [3] & Daniel F. Legler [1,4,5,*]

[1] Institute of Cell Biology and Immunology Thurgau (BITG), University of Konstanz, CH-8280 Kreuzlingen, Switzerland

[2] Graduate School for Cellular and Biomedical Sciences, University of Bern, CH-3012 Bern, Switzerland

[3] Biognosys AG, CH8952 Schlieren, Switzerland

[4] Theodor Kocher Institute, University of Bern, CH-3012 Bern, Switzerland

[5] Faculty of Biology, University of Konstanz, D-78464 Konstanz, Germany

[*] Correspondence: daniel.legler@bitg.ch

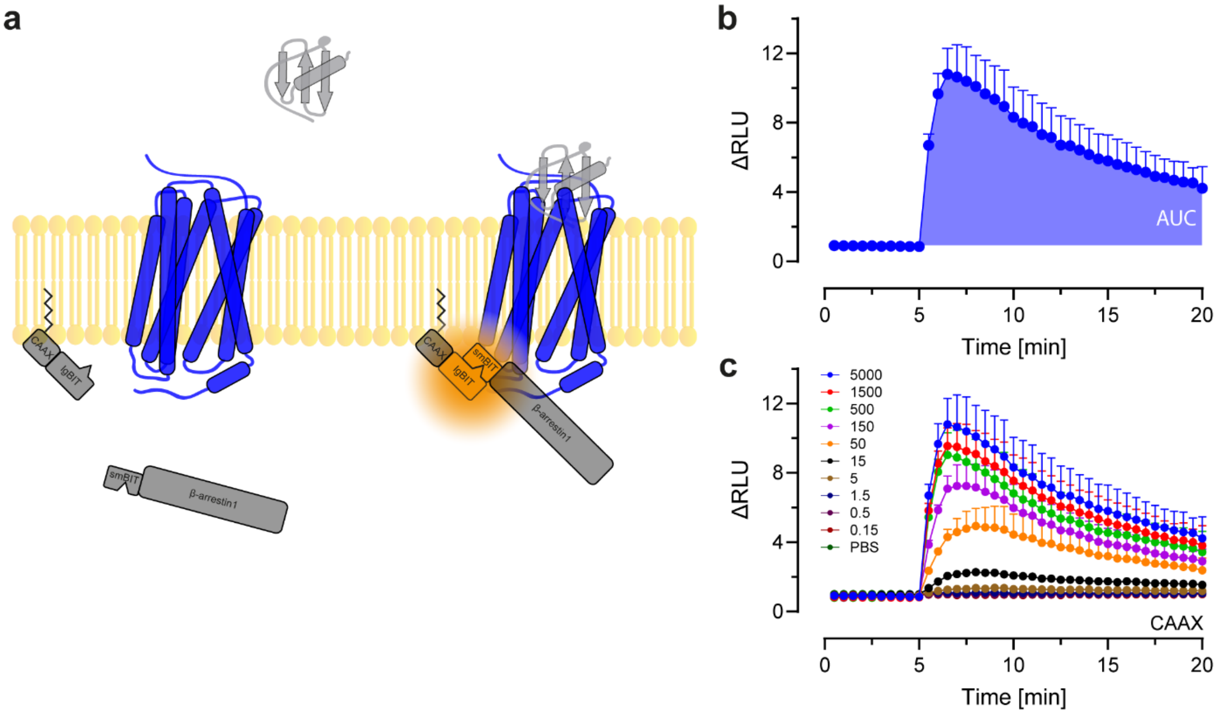

Supplementary Fig. 1: Principle and kinetics of the bystander split-luciferase assay (related to Fig. 1). **a** Exemplified overview of the utilised bystander split-luciferase assay depicting the reconstitution of smBiT and lgBiT to form a functional luciferase. Here, βarrestin1 fused to smBiT was recruited to lgBiT anchored to the plasma membrane through a CAAX domain upon chemokine stimulation of untagged, native ACKR4. **b** Changes in bioluminescence in HEK293 cells co-expressing untagged ACKR4 together with smBiT-tagged βarrestin1 and lgBiT-CAAX were measured before and after CCL19 or PBS addition at time point 5 minutes. The ΔRLU values were obtained by dividing the signals recorded from chemokine-stimulated cells by the signals recorded from cells stimulated with PBS. The mean of the first values (i.e. in the absence of ligand) were used to define the baseline of the assay and to calculate the area under the curve (AUC) between the baseline value and each measured change in bioluminescence over time after ligand stimulation. **c** Time-resolved experiment assessing smBiT-βarrestin1 recruitment to lgBiT-CAAX upon stimulation of ACKR4 expressing cells with graded concentrations (0.15-5000 nM) of CCL19 or PBS using the bystander assay; n = 3, mean ± SD. These data were used to generate the graph for smBiT-βarrestin1 recruitment to lgBiT-CAAX in response to ACKR4 stimulation depicted in Figure 1a.

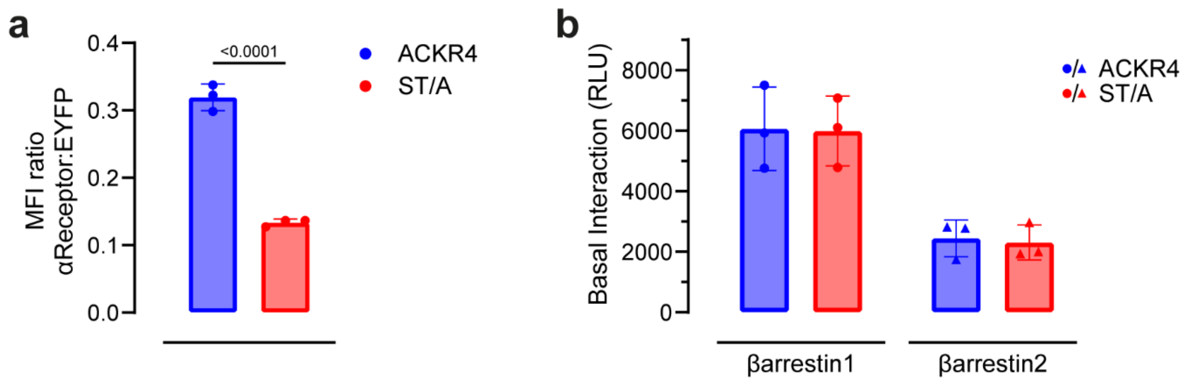

Supplementary Fig. 2: Reduced surface appearance of ACKR4 ST/A without affecting βarrestin association (related to Fig. 2). a MFI ratio of surface stained ACKR4 and co-expressed EYFP in HEK293 cells transiently transfected with ACKR4 or ACKR4 ST/A and EYFP; n = 3, mean ± SD. **b** Baseline interaction of βarrestins with the plasma membrane in the presence of either ACKR4 or ACKR4 ST/A. HEK293 cells transiently expressing ACKR4 or ACKR4 ST/A, together with lgBiT-CAAX and smBiT-βarrestin1 or smBiT-βarrestin2. Bioluminescence derived from the reconstituted nanoLuciferase was recorded and relative light units (RLU) are depicted; n = 3, mean ± SD. Statistical analysis: unpaired two-sided t test; exact P value is shown in the graph.

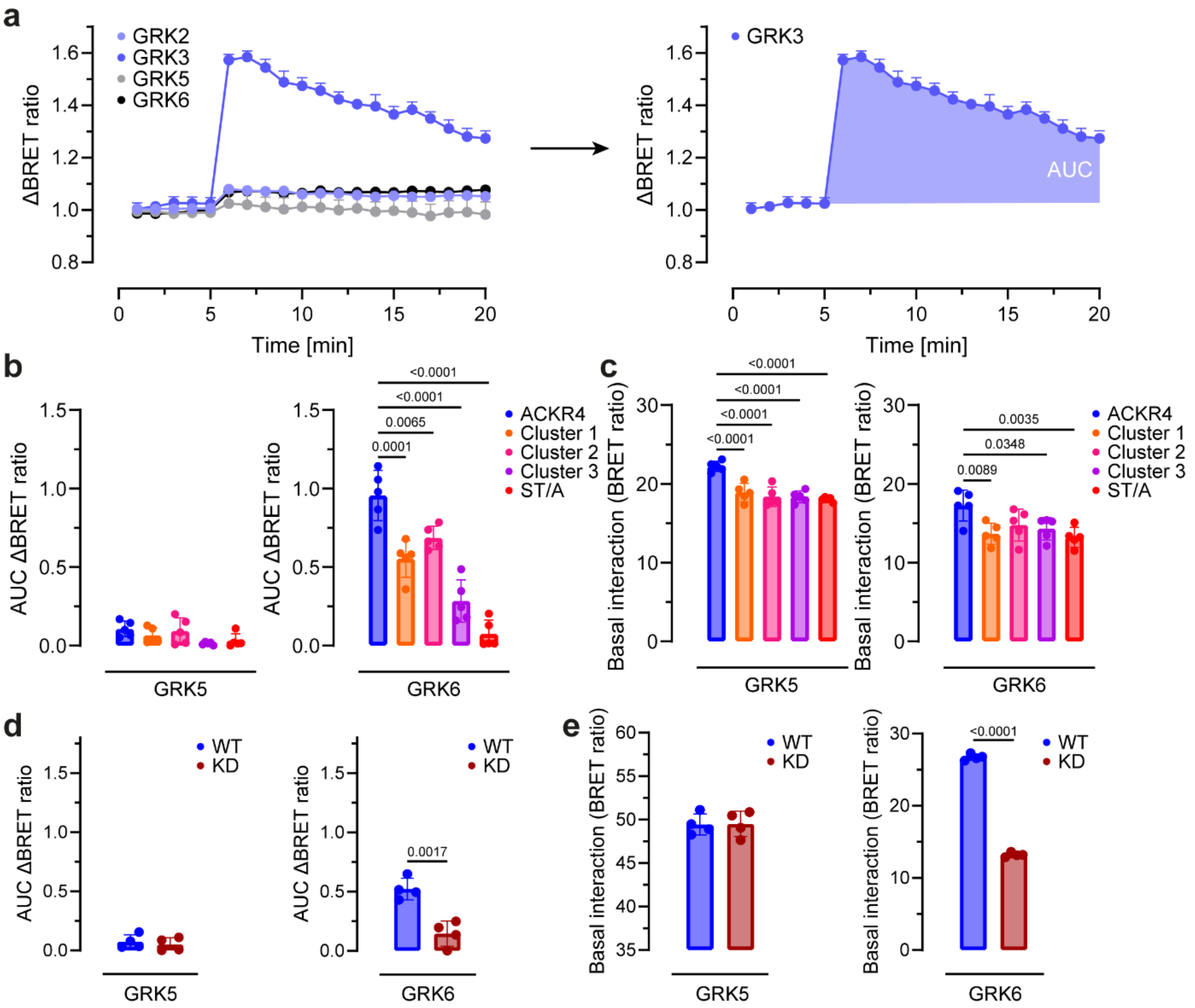

Supplementary Fig. 3: Changes in BRET of GRK5/6-rLuc8 and rGFP-CAAX related to mutations in C-terminal regions of ACKR4 (related to Fig. 4). a Representative changes in ΔBRET ratios over time between GRKs fused to rLuc8 and rGFP-CAAX in HEK293 cells co-expressing ACKR4 and addition of 1 µM of CCL19 after 5 min of recording (left). Area under the curve (AUC) determination exemplary for GRK3 (right). **b** Quantitative analysis (AUC) of ligand-mediated changes in the ΔBRET ratios between GRK5-rLuc8 (left) or GRK6-rLuc8 (right) and rGFP-CAAX in HEK293 cells co-expressing different ACKR4 variants; and **c** basal interaction (BRET ratio) in the absence of ligand; n = 5, mean ± SD. **d** Quantitative analysis (AUC) of ligand-mediated changes in the ΔBRET ratios between wildtype (WT) and kinase-dead (KD) variants of GRK5 (left) or GRK6 (right) fused to rLuc8 and rGFP-CAAX in HEK293 cells co-expressing ACKR4; and **e** basal interaction (BRET ratio) in the absence of ligand; n = 4, mean ± SD. Statistical analysis: **b c** ordinary one-way ANOVA, **d e** unpaired two-sided t-test; exact P values are shown in the graphs.

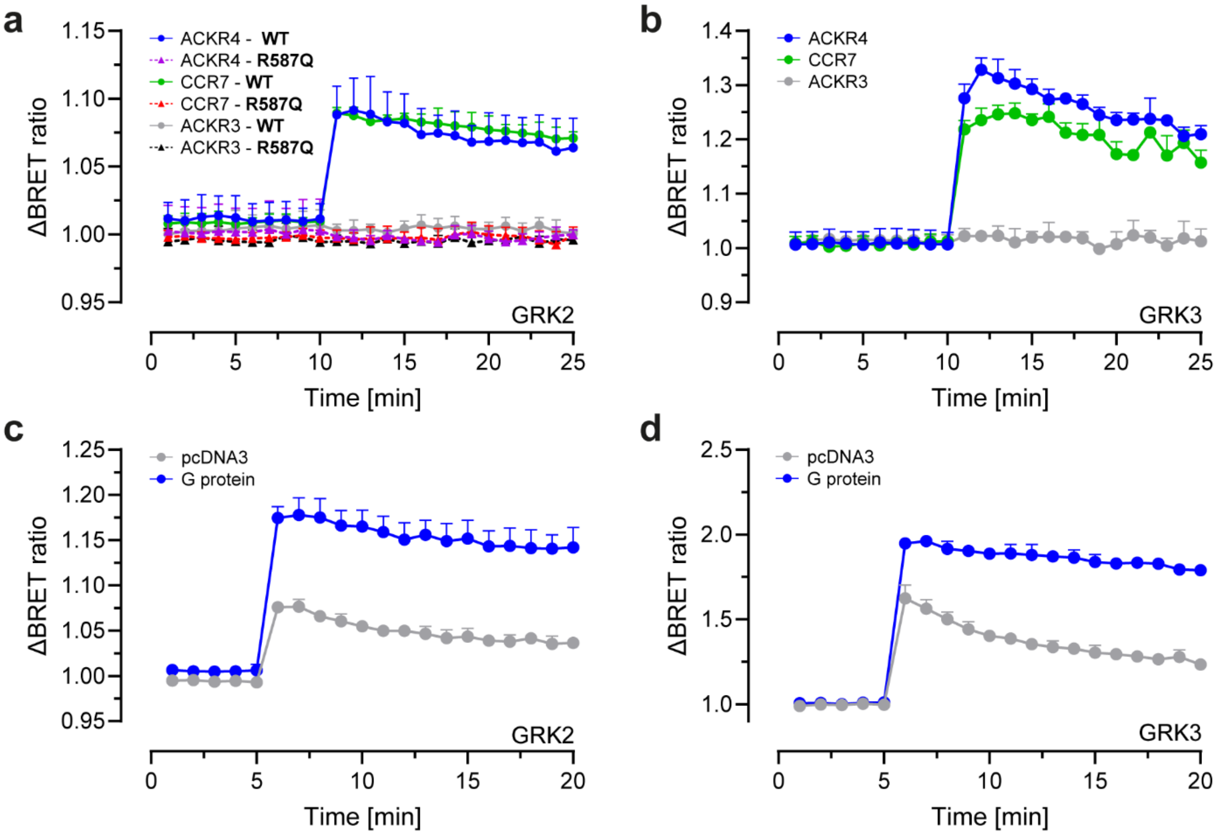

Supplementary Fig. 4: CCL19-induced changes in proximity between GRK2/3 variants and the G protein or the plasma membrane (related to Fig. 6). a Association of GRK2-nLuc (WT or R587Q) or **b** GRK3-nLuc with Gβγ-cpV/Gα_{i1} in HEK293 cells co-expressing either ACKR4, CCR7 or ACKR3 before and upon stimulation with 1 μM CCL19; n = 4 (**a**), n = 3 (**b**), mean ± SD. **c** Recruitment of GRK2-rLuc8 or **d** GRK3-rLuc8 to rGFP-CAAX in response to ACKR4 stimulation with 1 μM CCL19 in HEK293 cells transiently transfected with a vector encoding the heterotrimeric G protein or empty vector; n = 3, mean ± SD.

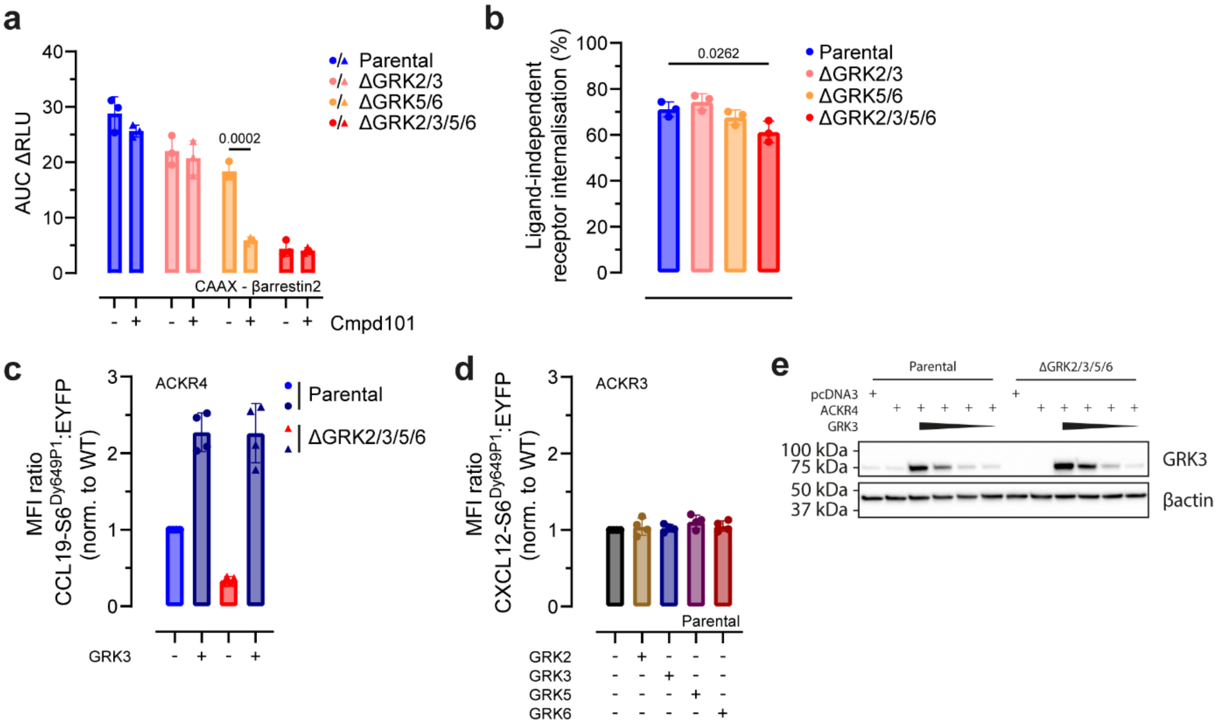

Supplementary Fig. 5: Characterisation of GRK KO cells and its reconstitution (related to Fig. 7). **a** smBit-βarrestin2 recruitment to lgBiT-CAAX at the plasma membrane in parental and GRK knockout cells co-expressing ACKR4 upon stimulation with 1 µM CCL19 in the presence of 10 µM cmpd101 or DMSO as vehicle control; n = 3, mean ± SD. **b** Ligand-independent receptor internalisation in parental, ΔGRK2/3, ΔGRK5/6 and ΔGRK2/3/5/6 cells; n = 3, mean ± SD. **c** Chemokine internalisation (5 nM for 30 min) of parental or GRK knockout cells transiently transfected with ACKR4 and reconstituted with GRK3 (ratio GRK3:ACKR4 - 1:48); n = 3, mean ± SD. **d** MFI ratio of fluorescently labelled CXCL12 taken up by HEK293A parental cells transiently transfected with ACKR3 and reconstituted with GRK2, GRK3, GRK5 or GRK6 (ratio GRKs:ACKR3 - 1:48); n = 3, mean ± SD. **e** Representative Western blot of ratio testing between GRK3 and ACKR4 in transiently transfected parental or ΔGRK2/3/5/6 cells (ratios GRK3:ACKR4 - 1:48, 1:100 , 1:500; 1:1000); n = 3. Statistical analysis: **a** unpaired two-sided t test, **b** ordinary one-way ANOVA, exact P values are shown in the graphs.

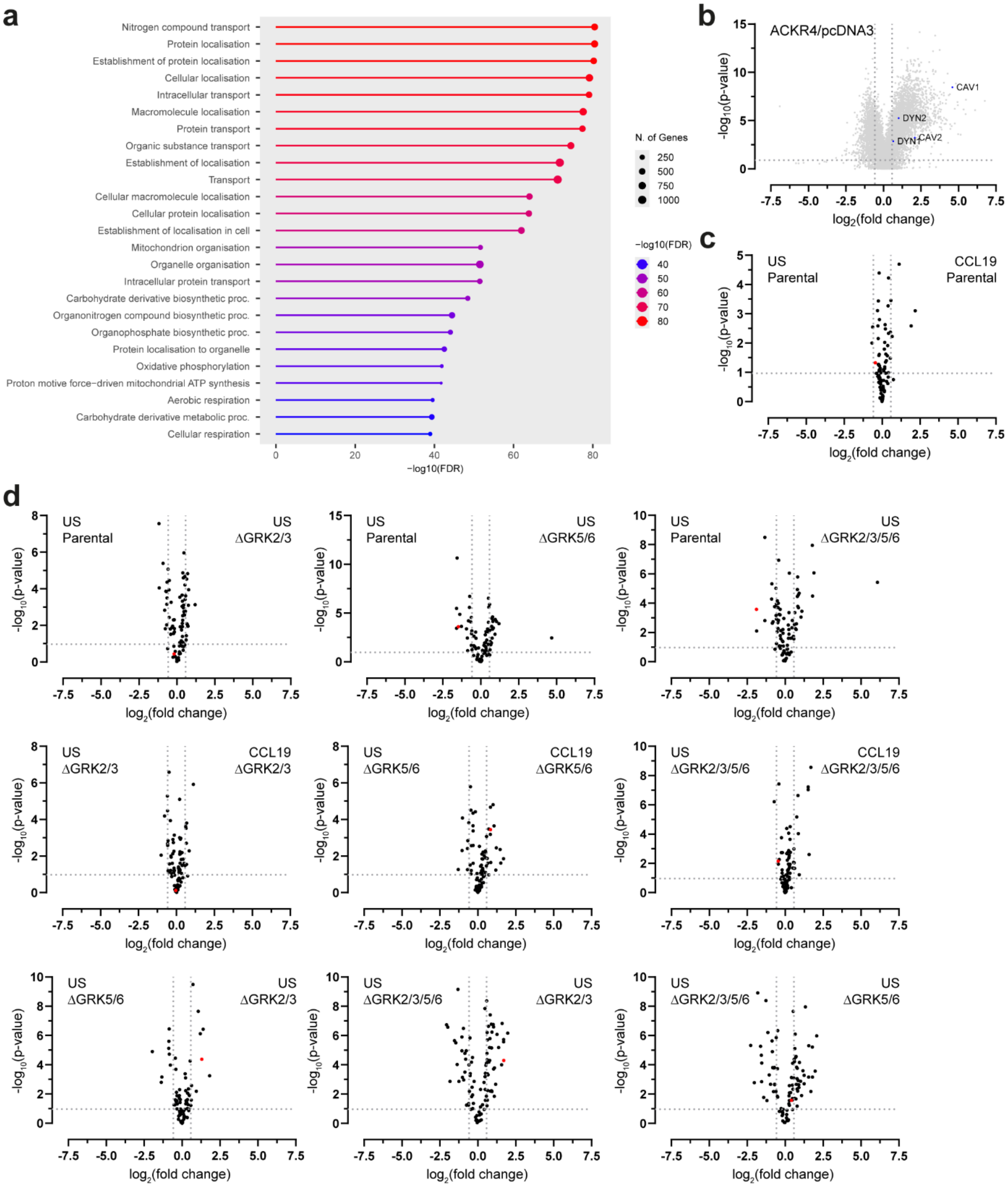

Supplementary Fig. 6: Pathway, interactome and phospho-peptide analysis in ACKR4-expressing cells revealed by mass spectrometry (related to Fig. 8). a Top 25 pathways upregulated in cells expressing ACKR4 using GO pathway database of biological processes. **b** Comparison of proteins found in MS analysis between pcDNA3 and ACKR4 transfected cells highlighting four proteins involved in endocytosis and associated in ACKR4 expressing samples. **c** Change in phosphorylation between unstimulated (US) and CCL19 stimulated HEK293 parental cells transiently transfected with ACKR4. **d** Comparison of HEK293 parental, ΔGRK2/3, ΔGRK5/6 and ΔGRK2/3/5/6 cells treated with buffer or 100 nM CCL19 regarding differences in protein phosphorylation. **c-d** Red dot indicates pSer349 of ACKR4. **a-d** Technical triplicates or duplicates (parental pcDNA3 + CCL19).

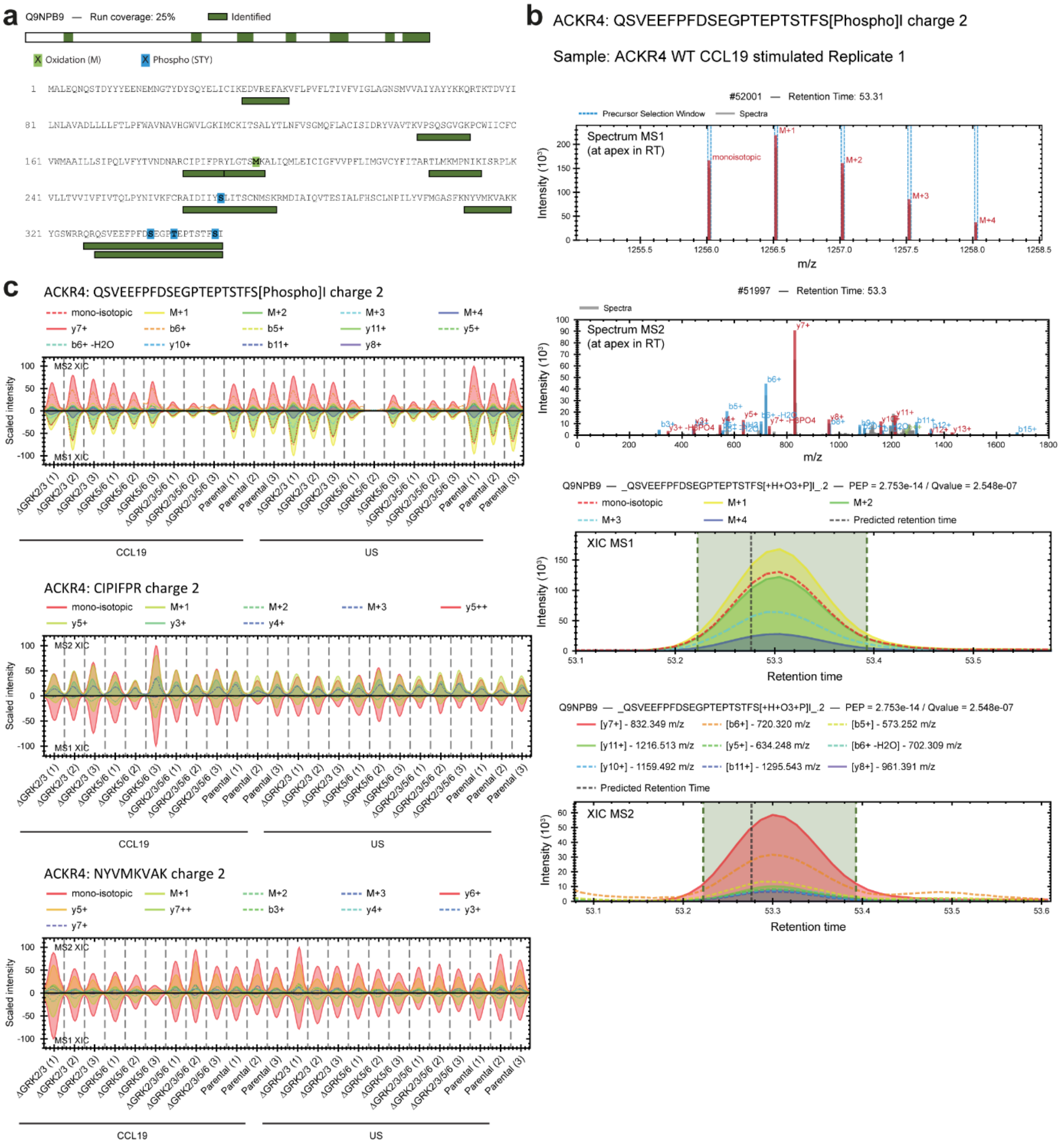

Supplementary Fig. 7: Proteomic identification of ACKR4 peptides (related to Fig. 8). a Identified peptides (dark green) and post translational modifications (light green – oxidation, blue – phosphorylation) in ACKR4 by mass spectrometry. **b** MS1 and MS2 spectra and extracted ion current of the tryptic peptide containing Ser349 phosphorylation (CCL19 stimulated parental HEK293 cells transiently expressing HA-tagged ACKR4). **c** Extracted ion current of the tryptic peptide containing Ser349 phosphorylation and two peptides of ACKR4 without post translational modification.

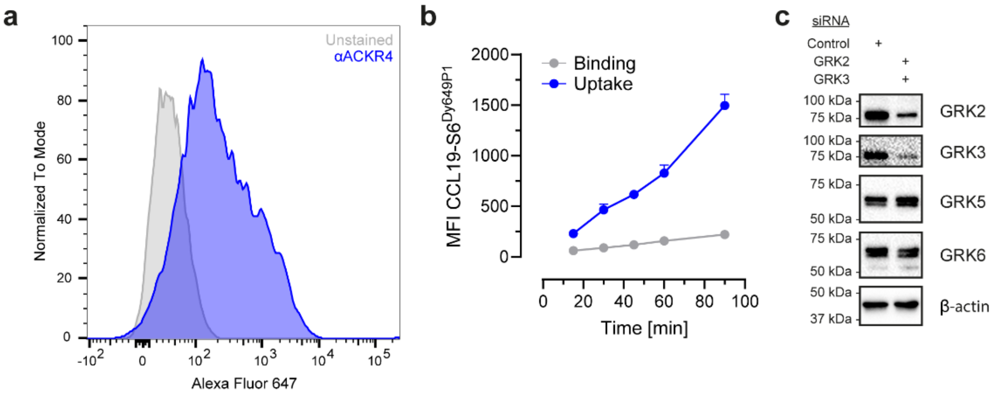

Supplementary Fig. 8: Endogenous expression of ACKR4 and internalisation of fluorescently labelled CCL19 by BJ hTERT human fibroblasts (related to Fig. 8). a ACKR4 surface expression determined by flow cytometry in BJ hTERT human fibroblasts; n = 1. **b** Chemokine binding (4 °C) and uptake (37 °C) over time in BJ hTERT human fibroblasts stimulated with 25 nM CCL19-S6^{Dy649P1}; n = 3, mean ± SD. **c** Western blot analysis of GRK expression after transient transfection of BJ hTERT human fibroblasts with either control siRNA or siRNA against GRK2 and GRK3; n = 4; representative replicate.

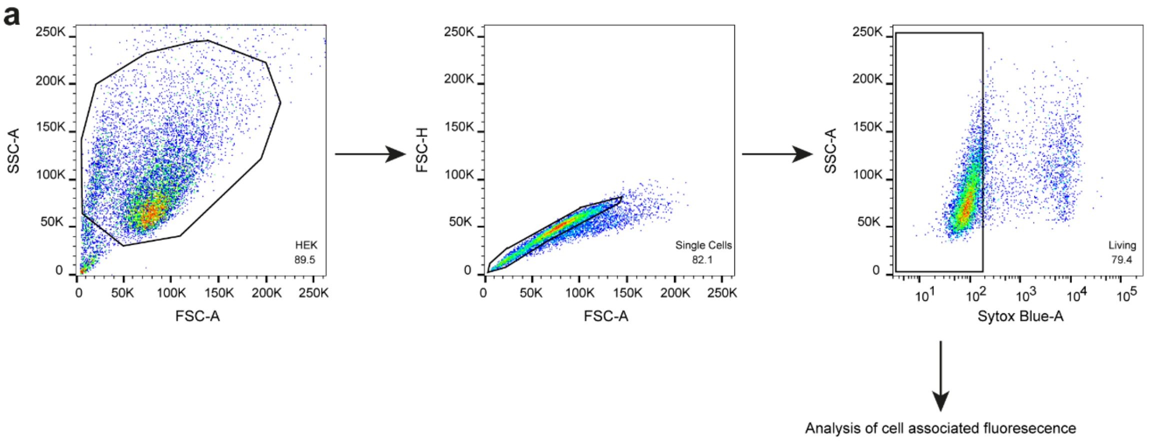

Supplementary Fig. 9: Common gating strategy for flow cytometry. a Exemplary flow cytometry dot plots showing the common gating strategy by means of FSC-A and SSC-A, followed by doublets exclusion and identification of living cells using a live dead discrimination. Afterwards, the cell associated fluorescence was measured which are indicated in the graph axes.

Supplementary Table 1: Plasmids for expression of recombinant chemokines.

Construct	Template (**amplified insert**)	5' forward primer	5' reverse primer	Restr. enzymes
pSUMO hCCL19	Published in [49]	-	-	-
pSUMO hCCL19-S6	Published in [49]	-	-	-
pSUMO hCXCL12-S6	pSUMO **hCXCL12** Published in [39]	CCCTCTAGAAATAATTTTGTTTAACTTTAAGAAGGAGATATACATATGG	GGTGCTCGAGTTATTAGTTCAGCAGGCGCAGCAGCCAGCTCAGGCTATCGCCGCTGCCGCCGCCGCCGCTGTTTAAAGCTTTCTCCAGGTACTCCTGAATCCAC	XhoI, XbaI

Supplementary Table 2: Expression plasmids for single proteins.

Construct	Template (**amplified insert**)	5' forward primer	5' reverse primer	Restr. enzymes
pcDNA3 ACKR4	Published in [39]	-	-	-
pcDNA3 CCR7	pcDNA3 **CCR7**-EGFP Published in [23]	CGAAATTAATACGACTCACTATAGGGAGACCC	TTAACTCGAGTCCACCTGGGGAGAAGGTGGTGGTGGTCTCG	EcoRI, XhoI
pcDNA3 ACKR3	Published in [39]	-	-	-
pcDNA3 lgBiT-CAAX	Published in [39]	-	-	-
pcDNA3 lgBiT-FYVE	pcDNA3 **lgBIT**-CAAX	GGTAAAGCTAGCATGGTCTTCACACTCG	GGAACTGGATCCGTTGATGGTTACTCGGAAC	NheI, BamHI
pcDNA3 smBiT-βarrestin1	Published in [39]	-	-	-
pcDNA3 smBiT-βarrestin2	Published in [39]	-	-	-
pcDNA3 EYFP	Published in [39]	-	-	-
pcDNA3 rGFP-CAAX	Published in [47]	-	-	-
pcDNA3 GRK2-nLuc	Published in [35]	-	-	-
pcDNA3 GRK3-nLuc	Published in [35]	-	-	-
pcDNA3 GRK2-rLuc8	pcDNA3 **GRK2**-nLuc Published in [35]	CGAAATTAATACGACTCACTATAGGGAGACCC	TTAAGGATCCGAGGCCGTTGGCACTGCCGCGCTGGACC	HindIII, BamHI
pcDNA3 GRK3-rLuc8	pcDNA3 **GRK3**-nLuc Published in [35]	CGAAATTAATACGACTCACTATAGGGAGACCC	TTAAGGATCCGAGGCCGTTGCTGTTTCTGTGACAGAGG	HindIII, BamHI
pcDNA3 GRK5-rLuc8	pcDNA3 **GRK5**-nLuc Published in [35]	CGAAATTAATACGACTCACTATAGGGAGACCC	TTAAGGATCCGCTGCTTCCGGTGGAGTTCGAGCTGACATGG	HindIII, BamHI
pcDNA3 GRK6B-rLuc8	pcDNA3 **GRK6B**-nLuc Published in [35]	CGAAATTAATACGACTCACTATAGGGAGACCC	TTAAGGATCCCCGCCAACTGCTGGTGGGGGCCTCG	HindIII, BamHI
pcDNA3 GRK2	pcDNA3 **GRK2**-nLuc	CGAAATTAATACGACTCACTATAGGGAGACCC	TTAATCTAGATTAGAGGCCGTTGGCACTGCCGCGCTGG	HindIII, XbaI
pcDNA3 GRK3	pcDNA3 **GRK3**-nLuc	CGAAATTAATACGACTCACTATAGGGAGACCC	TTAATCTAGATTAGAGGCCGTTGCTGTTTCTGTGACAGAGG	HindIII, XbaI
pcDNA3 GRK5	pcDNA3 **GRK5**-nLuc	CGAAATTAATACGACTCACTATAGGGAGACCC	TTAATCTAGATTAGCTGCTTCCGGTGGAGTTCGAGCTGACATGG	HindIII, XbaI
pcDNA3 GRK6B	pcDNA3 **GRK6B**-nLuc	CGAAATTAATACGACTCACTATAGGGAGACCC	TTAATCTAGATTACCGCCAACTGCTGGTGGGGGCCTCGGGCTGG	HindIII, XbaI
pcDNA3 HA-ACKR4	Published in [39]	-	-	-
pcDNA3 3xFLAG-GRK2	pcDNA3 **GRK2**	AAGCTTGCCACCATGGACTACAAGGACGACGACGACAAGGACTACAAGGACGACGACGACAAGGACTACAAGGACGACGACGACAAGAGCGGCGGTGGAGGATCCGCGGACCTGGAGG	GCGAGCTCTAGCATTTAGGTG	HindIII, XbaI

Supplementary Table 3: Expression plasmids encoding for two proteins.

Construct	Template (**amplified insert**)	5' forward primer	5' reverse primer	Restr. enzymes
pIRES EYFP_ACKR4	Published in [39]	-	-	-
pIRES EYFP_CCR7	pcDNA3 **CCR7**	AATTGCGGCCGCAATGGACCTGGGGAAACCAATGAAAAGC	TTAATCTAGATTATTATGGGGAGAAGGTGGTGGTGG	NotI, XbaI
pIRES EYFP_ACKR4 ST/A	pcDNA3 **ACKR4 ST/A**	ATATGGCGGCCGCAATGGCTTTGGAACAAAATCAATCTACC	GCGAGCTCTAGCATTTAGGTG	NotI, XbaI
pIRES EYFP_ACKR4 Cluster 1	pcDNA3 **ACKR4 Cluster 1**	ATATGGCGGCCGCAATGGCTTTGGAACAAAATCAATCTACC	GCGAGCTCTAGCATTTAGGTG	NotI, XbaI
pIRES EYFP_ACKR4 Cluster 2	pcDNA3 **ACKR4 Cluster 2**	ATATGGCGGCCGCAATGGCTTTGGAACAAAATCAATCTACC	GCGAGCTCTAGCATTTAGGTG	NotI, XbaI
pIRES EYFP_ACKR4 Cluster 3	pcDNA3 **ACKR4 Cluster 3**	ATATGGCGGCCGCAATGGCTTTGGAACAAAATCAATCTACC	GCGAGCTCTAGCATTTAGGTG	NotI, XbaI
pIRES EYFP_ACKR3	pcDNA3 **ACKR3** Published in [39])	AATTGCGGCCGCTACCCGTCATGGATCTGCATCTCTTCGACTACTCAGAGC	TTAATCTAGATCATCATTTGGTGCTCTGCTCCAAGG	NotI, XbaI
pIRES Gβ1-T2A-cpV-Gγ2_Gαi1-nLuc	Published in [35]	-	-	-
pIRES Gβ1-T2A-cpV-Gγ2_Gαi2-nLuc	GNB1-GNG2-**GNAI2** (Addgene #168121)	TTAAAAGCTTCAGCCACCATGGGCTGCACCGTGAGCGC	AAGTAAAACCTCTACAAATGTGGTATGG	HindIII, XbaI
pIRES Gβ1-T2A-cpV-Gγ2_Gαo-nLuc	pcDNA3 **GNAO-nLuc** Published in [35]	CGAAATTAATACGACTCACTATAGGGAGACCC	GCGAGCTCTAGCATTTAGGTG	HindIII, XbaI
pIRES Gβ1-T2A-cpV-Gγ1_Gαs-nLuc	**GNB1-GNG1**-GNAS (Addgene #168124)	GCTAGCGCTACCGGTCGCCACCATGGGGGAGCTTGACCAGTTACG	TTAAGCGGCCGCTTATGAAATCACACACCCTCC	NheI, NotI
	GNB1-GNG1-**GNAS**	TTAAAAGCTTCAGCCACCATGGGCTGCCTCGGCAACAGTAAGACC	AAGTAAAACCTCTACAAATGTGGTATGG	HindIII, XbaI
pIRES Gβ3-T2A-cpV-Gγ9_Gαq-nLuc	**GNB3-GNG9**-GNAQ (Addgene #168125)	TTAAGCTAGCGCTACCGGTCGCCACCATGGGGGAGATGGAGCAACTGC	TTAAGCGGCCGCTTAGCTTATCAGACAGCCACC	NheI, NotI
	pcDNA3 **GNAQ-nLuc** Published in [35]	CGAAATTAATACGACTCACTATAGGGAGACCC	GCGAGCTCTAGCATTTAGGTG	HindIII, XbaI
pIRES Gβ3-T2A-cpV-Gγ9_Gα12-nLuc	**GNB3-GNG9**-GNAQ	TTAAGCTAGCGCTACCGGTCGCCACCATGGGGGAGATGGAGCAACTGC	TTAAGCGGCCGCTTAGCTTATCAGACAGCCACC	NheI, NotI
	GNB3-GNG9-**GNA12** (Addgene #190714)	TTAACTCGAGCAGCCACCATGTCCGGGGTGGTGCGGACC	AAGTAAAACCTCTACAAATGTGGTATGG	XhoI, XbaI
pIRES Gβ3-T2A-cpV-Gγ9_Gα13-nLuc	**GNB3-GNG9**-GNAQ	TTAAGCTAGCGCTACCGGTCGCCACCATGGGGGAGATGGAGCAACTGC	TTAAGCGGCCGCTTAGCTTATCAGACAGCCACC	NheI, NotI
	pcDNA3 **GNA13-nLuc** Published in [35]	CGAAATTAATACGACTCACTATAGGGAGACCC	GCGAGCTCTAGCATTTAGGTG	HindIII, XbaI

pIRES Gβ3-T2A-cpV-Gγ9_Gα15-nLuc	**GNB3-GNG9**-GNAQ	TTAAGCTAGCGCTACCGGTCGCCACCATGGGGGAGATGGAGCAACTGC	TTAAGCGGCCGCTTAGCTTATCAGACAGCCACC	NheI, NotI
	GNB3-GNG9-**GNA15** (Addgene #168126)	TTAAAAGCTTCAGCCACCATGGCCCGCTCGCTGACC	AAGTAAAACCTCTACAAATGTGGTATGG	HindIII, XbaI
pIRES Gβ1-T2A-cpV-Gγ2_Gαi1	pcDNA3 **Gαi1** Published in [35**]**	CGAAATTAATACGACTCACTATAGGGAGACCC	GCGAGCTCTAGCATTTAGGTG	HindIII, XbaI
pIRES Gβ1-T2A-Gγ2_Gαi1	pIRES **Gβ1-T2A**-cpV-Gγ2_Gαi1	TTAAGAATTCGCCACCATGGGGGAGCTTGACCAGTTACGGCAGGAGG	TTAACTCGAGAGGGCCGGGATTCTCCTCCACGTCACCGC	EcoRI, XhoI
	pIRES Gβ1-T2A-cpV-**Gγ2**_Gαi1	TTAACTCGAGATGGCCAGCAACAACACCGCCAGCATAGC	GATTATGATCTAGATCAAAAGAGACCACAATCTTTTAGATTATTTTTTATG	XhoI, HindIII

Supplementary Table 4: Primers used to introduce indicated mutations.

Construct	Template (**amplified insert**)	5' forward primer	5' reverse primer	Restr. enzymes
pcDNA3 ACKR4 ST/A (S309A, S323A, S330A, S338A, T342A, T345A, S346A, T347A, S349A)	pcDNA3 **ACKR4**	GTTTTTATGGGTGCCGCTTTCAAGAACTACG	CGTAGTTCTTGAAAGCGGCACCCATAAAAAC	-
		GCTAAGAAGTACGGTGCTTGGAGAAGACAAAG	CTTTGTCTTCTCCAAGCACCGTACTTCTTAGC	-
		GAGAAGACAAAGACAAGCCGTTGAAGAATTCC	GGAATTCTTCAACGGCTTGTCTTTGTCTTCTC	-
		GAATTCCCATTCGATGCTGAAGGTCCAGCTG	CAGCTGGACCTTCAGCATCGAATGGGAATTC	-
		CGAAATTAATACGACTCACTATAGGGAGACCC	CCTTGGAAGCCATCTCGAGCTAAATAGCGAAGGCAGCAGCAGG	HindIII, XhoI
pcDNA3 ACKR4 Cluster 1 (S309A, S323A, S330A)	pcDNA3 **ACKR4**	GTTTTTATGGGTGCCGCTTTCAAGAACTACG	CGTAGTTCTTGAAAGCGGCACCCATAAAAAC	-
		GCTAAGAAGTACGGTGCTTGGAGAAGACAAAG	CTTTGTCTTCTCCAAGCACCGTACTTCTTAGC	-
		GAGAAGACAAAGACAAGCCGTTGAAGAATTCC	GGAATTCTTCAACGGCTTGTCTTTGTCTTCTC	-
pcDNA3 ACKR4 Cluster 2 (S338A, T342A)	pcDNA3 **ACKR4**	GAATTCCCATTCGATGCTGAAGGTCCAGCTG	CAGCTGGACCTTCAGCATCGAATGGGAATTC	-
pcDNA3 ACKR4 Cluster 3 (T345A, S346A, T347A, S349A)	pcDNA3 **ACKR4**	CGAAATTAATACGACTCACTATAGGGAGACCC	CCTTGGAAGCCATCTCGAGCTAAATAGCGAAGGCAGCAGCAGG	HindIII, XhoI
pcDNA3 GRK2-rLuc8 K220R	pcDNA3 GRK2-rLuc8	CAAGATGTACGCCATGAGGTGCCTGGACAAAAAG	CTTTTTGTCCAGGCACCTCATGGCGTACATCTTG	-
pcDNA3 GRK3-rLuc8 K220R	pcDNA3 GRK3-rLuc8	GGAAAAATGTATGCAATGAGATGCTTAGATAAGAAGAGG	CCTCTTCTTATCTAAGCATCTCATTGCATACATTTTTCC	-
pcDNA3 GRK5-rLuc8 K215R	pcDNA3 GRK5-rLuc8	GTAAAATGTATGCCTGCAGGCGCTTGGAGAAGAAGAG	CTCTTCTTCTCCAAGCGCCTGCAGGCATACATTTTAC	-
pcDNA3 GRK6-rLuc8 K215R/K216R	pcDNA3 GRK6-rLuc8	GTAAGATGTATGCCTGCAGGAGGCTAGAGAAAAAGCGGATC	GATCCGCTTTTTCTCTAGCCTCCTGCAGGCATACATCTTAC	-
pcDNA3 GRK2-rLuc8 D110A	pcDNA3 GRK2-rLuc8	CCGGGAGATCTTCGCCTCATACATCATG	CATGATGTATGAGGCGAAGATCTCCCGG	-
pcDNA3 GRK2-rLuc8 R587Q	pcDNA3 GRK2-rLuc8	CTGTTCCCCAACCAGCTCGAGTGGCGG	CCGCCACTCGAGCTGGTTGGGGAACAG	-
pcDNA3 GRK2-nLuc R587Q	pcDNA3 GRK2-nLuc	CTGTTCCCCAACCAGCTCGAGTGGCGG	CCGCCACTCGAGCTGGTTGGGGAACAG	-
pcDNA3 ACKR4 TST345AAA (T345A, S346A, T347A)	pcDNA3 **ACKR4**	CGAAATTAATACGACTCACTATAGGGAGACCC	AATTCTCGAGTTAAATAGAGAAGGCAGCAGCAGGTTCAGTTGGACCTTCAGAATCG	HindIII, XhoI
