# Supplementary material for: GPCR kinases shape ACKR4 functions via differential C-terminal phosphorylation
Source: Nat Commun. 2026 May 16;17:6503. doi: 10.1038/s41467-026-73074-4 (PMC13377056; doi:10.1038/s41467-026-73074-4)
Supplement: Supplementary file 2 — Reporting Summary [file 41467_2026_73074_MOESM2_ESM.pdf]

nature portfolio

Corresponding author(s): Daniel F. Legler

Last updated by author(s): Apr 8, 2026

Reporting Summary

Nature Portfolio wishes to improve the reproducibility of the work that we publish. This form provides structure for consistency and transparency in reporting. For further information on Nature Portfolio policies, see our Editorial Policies and the Editorial Policy Checklist.

Statistics

For all statistical analyses, confirm that the following items are present in the figure legend, table legend, main text, or Methods section.

n/a	Confirmed	
☐	☒	The exact sample size (*n*) for each experimental group/condition, given as a discrete number and unit of measurement
☐	☒	A statement on whether measurements were taken from distinct samples or whether the same sample was measured repeatedly
☐	☒	The statistical test(s) used AND whether they are one- or two-sided<br>*Only common tests should be described solely by name; describe more complex techniques in the Methods section.*
☒	☐	A description of all covariates tested
☐	☒	A description of any assumptions or corrections, such as tests of normality and adjustment for multiple comparisons
☐	☒	A full description of the statistical parameters including central tendency (e.g. means) or other basic estimates (e.g. regression coefficient) AND variation (e.g. standard deviation) or associated estimates of uncertainty (e.g. confidence intervals)
☐	☒	For null hypothesis testing, the test statistic (e.g. *F*, *t*, *r*) with confidence intervals, effect sizes, degrees of freedom and *P* value noted<br>*Give P values as exact values whenever suitable.*
☒	☐	For Bayesian analysis, information on the choice of priors and Markov chain Monte Carlo settings
☒	☐	For hierarchical and complex designs, identification of the appropriate level for tests and full reporting of outcomes
☒	☐	Estimates of effect sizes (e.g. Cohen's *d*, Pearson's *r*), indicating how they were calculated

Our web collection on statistics for biologists contains articles on many of the points above.

Software and code

Policy information about availability of computer code

Data collection	No code was used in this study.
Data analysis	GraphPad Prism (v10.1.2) was used for statistical analysis. Spectronaut software (v19.4), Image Lab software v4.1, BD FACSDivaTM (v9.0.1) and FlowJO (v10.8.1) were used for analysis.

For manuscripts utilizing custom algorithms or software that are central to the research but not yet described in published literature, software must be made available to editors and reviewers. We strongly encourage code deposition in a community repository (e.g. GitHub). See the Nature Portfolio guidelines for submitting code & software for further information.

Data

Policy information about availability of data

All manuscripts must include a data availability statement. This statement should provide the following information, where applicable:

- Accession codes, unique identifiers, or web links for publicly available datasets
- A description of any restrictions on data availability
- For clinical datasets or third party data, please ensure that the statement adheres to our policy

The original datasets supporting the conclusions of this study are available on Zenodo doi:10.5281/zenodo.18888050. The raw MS data, and the quantitative data tables have been deposited to the ProteomeXchange Consortium via the MassIVE partner repository with the dataset identifier MSV000098965. The saved projects from Spectronaut can be viewed with the Spectronaut Viewer (www.biognosys.com/spectronaut-viewer). The following accession code was used in this study: 6U7C [https://doi.org/10.2210/pdb6u7c/pdb]; structure of human GRK2.

Research involving human participants, their data, or biological material

Policy information about studies with human participants or human data. See also policy information about sex, gender (identity/presentation), and sexual orientation and race, ethnicity and racism.

Reporting on sex and gender	This study does not invovlve human data or participants.
Reporting on race, ethnicity, or other socially relevant groupings	N/A
Population characteristics	N/A
Recruitment	N/A
Ethics oversight	N/A

Note that full information on the approval of the study protocol must also be provided in the manuscript.

Field-specific reporting

Please select the one below that is the best fit for your research. If you are not sure, read the appropriate sections before making your selection.

[x] Life sciences [ ] Behavioural & social sciences [ ] Ecological, evolutionary & environmental sciences

For a reference copy of the document with all sections, see nature.com/documents/nr-reporting-summary-flat.pdf

Life sciences study design

All studies must disclose on these points even when the disclosure is negative.

Sample size	No sample size calculations were performed. Sample size was based on current standards in the field.
Data exclusions	No data was excluded.
Replication	All experiments were performed in sufficient replicates, as indicated in the figure legends, and were succesful in its reproducibility.
Randomization	Randomization is not relevant to the experimental results of the manuscript.
Blinding	Blinding is not relevant to the experimental results of the manuscript.

Reporting for specific materials, systems and methods

We require information from authors about some types of materials, experimental systems and methods used in many studies. Here, indicate whether each material, system or method listed is relevant to your study. If you are not sure if a list item applies to your research, read the appropriate section before selecting a response.

Materials & experimental systems

n/a	Involved in the study	
[ ]	[x]	Antibodies
[ ]	[x]	Eukaryotic cell lines
[x]	[ ]	Palaeontology and archaeology
[x]	[ ]	Animals and other organisms
[x]	[ ]	Clinical data
[x]	[ ]	Dual use research of concern
[x]	[ ]	Plants

Methods

n/a	Involved in the study	
[x]	[ ]	ChIP-seq
[ ]	[x]	Flow cytometry
[x]	[ ]	MRI-based neuroimaging

Antibodies

Antibodies used

Flow cytometry
ACKR4 - Biolegend; #362102; secondary anti-mouse - ThermoFisher, #A-21235; CCR7 - ThermoFisher, #17-1979-42

Western blot analysis
beta-actin – Abcam, #ab6276 (1:5000); ACKR4 -7TM Antibodies, #7TM0315N (1:1000); pACKR4 - 7TM Antibodies, #7TM0315A (1:1000); GRK2 – Santa Cruz, #sc-13143 (1:500); GRK3 – Cell Signaling, #80362 (1:500); GRK5 – Santa Cruz, #sc-518005 (1:500); GRK6 – Cell Signaling, #5878 (1:1000); GFP - Abcam, #32146 (1:10000) FLAG - Sigma-Aldrich, #A8592 (1:3000); goat-anti-mouse – Jackson

	ImmunoResearch, #115-035-003 (1:5000); goat-anti-rabbit - Jackson ImmunoResearch, #111-035-003 (1:5000).
Validation	All antibodies are commercially available and have been validated by the indicated manufacturers.

Eukaryotic cell lines

Policy information about cell lines and Sex and Gender in Research

Cell line source(s)	HEK293 cells (ATCC CRL-1573)<br>HEK293A GRK parental, deltaGRK2/3, deltaGRK5/6, deltaGRK2/3/5/6 cells (Kawakami et al., 2022, Heterotrimeric Gq proteins act as a switch for GRK5/6 selectivity underlying β-arrestin transducer bias, Nature communciations)<br>HEK293A parental G protein, deltaGs/q/12 cells (Grundmann et al., 2018, Lack of beta-arrestin signaling in the absence of active G proteins, Nature communications)<br>BJ hTERT cells (ATCC CRL-3627)
Authentication	GRK KO cell lines were authenticated (see Western blots in results section), otherwise cell lines were not authenticated.
Mycoplasma contamination	Cell lines were tested negative for mycoplasma contamination.
Commonly misidentified lines (See ICLAC register)	None.

Plants

Seed stocks	N/A
Novel plant genotypes	N/A
Authentication	N/A

Flow Cytometry

Plots

Confirm that:

- [x] The axis labels state the marker and fluorochrome used (e.g. CD4-FITC).
- [x] The axis scales are clearly visible. Include numbers along axes only for bottom left plot of group (a 'group' is an analysis of identical markers).
- [x] All plots are contour plots with outliers or pseudocolor plots.
- [ ] A numerical value for number of cells or percentage (with statistics) is provided.

Methodology

Sample preparation	Sample preparation was described in detail in the method section
Instrument	BD LSRFortessa
Software	BD FACSDivaTM software v9.0.1 & FlowJo v10.8.1 software
Cell population abundance	All experiments involved transiently transfected cell lines and the stopping gate was set to 10 000 living cells.
Gating strategy	Gating strategy is provided in supplementary figure 9.

- [x] Tick this box to confirm that a figure exemplifying the gating strategy is provided in the Supplementary Information.
