# Supplementary material for: GPCR kinases shape ACKR4 functions via differential C-terminal phosphorylation
Source: Nat Commun. 2026 May 16;17:6503. doi: 10.1038/s41467-026-73074-4 (PMC13377056; doi:10.1038/s41467-026-73074-4)
Supplement: Supplementary file 3 — Transparent Peer Review file [file 41467_2026_73074_MOESM3_ESM.pdf]

nature portfolio

Peer Review File

GPCR kinases shape ACKR4 functions via differential C-terminal phosphorylation

Corresponding Author: Professor Daniel Legler

Editorial Note: Parts of this Peer Review File have been redacted as indicated to remove third party material where no permission to publish were obtained.

This file contains all reviewer reports in order by version, followed by all author rebuttals in order by version.

Version 0:

Reviewer comments:

Reviewer #1

(Remarks to the Author)
This is a well-designed and clearly written manuscript by Gerken et al. that investigates the complex regulation of the atypical chemokine receptor 4 (ACKR4) by G protein-coupled receptor kinases (GRKs). The authors use a comprehensive and technically sound experimental approach, including β-arrestin recruitment assays, chemokine uptake measurements, GRK knockout and rescue experiments, and mass spectrometry-based phosphorylation analysis. The central advance of this work is the meticulous dissection of how the four non-visual GRKs (2, 3, 5, and 6) differentially regulate ACKR4's chemokine scavenging function. A key strength of the study is the use of native, untagged ACKR4, which circumvents artifacts associated with C-terminal tags that the authors' own previous work identified. Notably, the authors convincingly demonstrate that ACKR4 constitutively cycles and associates with β-arrestins. They identify distinct C-terminal serine/threonine clusters that are critical for steady-state trafficking and ligand-induced internalization. The study provides compelling evidence for a division of labor among GRKs, with GRK5/6 primarily regulating steady-state phosphorylation and GRK2/3 being recruited upon ligand stimulation to enhance chemokine uptake. Most notably, the authors suggest a paradigm-challenging finding that GRK2/3 recruitment to ACKR4 occurs independently of G protein activation.

While the paper presents a thorough and detailed molecular dissection of ACKR4, a critique could argue that several of its core findings confirm mechanisms that were already well-established for its more famous family member, ACKR3. Here are points where the novelty could be considered limited, particularly in the context of existing ACKR3 literature. First, the paper emphasizes that GRK2 and GRK3 are recruited to ACKR4 without any G protein activation, framing this as a challenge to the existing paradigm. However, this G protein-independent, β-arrestin-biased signaling is the defining characteristic of the entire ACKR family, with ACKR3 being the archetypal example. ACKR3 is famously known to recruit GRKs and signal through β-arrestin without coupling to G proteins. Therefore, demonstrating this same core mechanism in ACKR4 is more of a confirmation of a shared family trait rather than a fundamentally new discovery. Second, the paper establishes that ACKR4 constitutively associates with β-arrestins and cycles between the plasma membrane and endosomes, even in its apo (ligand-free) state. Again, ACKR3 is well-known for its high level of constitutive activity and β-arrestin interaction, which drives its continuous trafficking. The authors' use of a new assay to study the untagged receptor is a methodological advance, but the underlying biological concept of constitutive activity was already a known feature of this receptor subfamily.

The authors may overstate the novelty of their findings for ACKR4 by not sufficiently comparing them with the well-established mechanisms of ACKR3. Since ACKRs have been delineated from GPCRs based on shared characteristics—such as their inability to couple to G proteins—it is important that the data is analyzed and discussed within this shared framework. While the experiments are robust and the data generally support the conclusions, several key areas in the interpretation and discussion could be strengthened to further solidify the manuscript's impact.

In this context, the authors may overstate the novelty of their findings for ACKR4 by not sufficiently comparing them with the well-established mechanisms of ACKR3. The discussion should be revised to contextualize the results within the broader ACKR family, which would ultimately strengthen the manuscript's conclusions. Since ACKRs have been delineated from GPCRs based on shared characteristics—such as their inability to couple to G proteins—it is important that the data is analyzed and discussed within this shared framework. Overall, while the experiments are robust and the data generally support the conclusions, several major issues should be addressed to fully solidify the manuscript's claims.

Major issues

1. The conclusion that GRK2 interacts with the inactive heterotrimeric G protein is one of the most provocative and potentially impactful claims in the manuscript. However, this conclusion currently rests on limited and indirect experimental support, primarily the partial reduction in recruitment observed with the GRK2 R587Q mutant. While suggestive, this single piece of data is not sufficient to rule out alternative interpretations, such as non-specific effects of the mutation on protein stability or membrane affinity. To substantiate this paradigm-shifting claim, further and more direct experimental evidence is required. For instance, the authors could perform co-immunoprecipitation experiments, pulling down ACKR4 and probing for the presence of both GRK2 and inactive G protein subunits (e.g., in the presence of GDP) to test for a ternary complex. Furthermore, in vitro binding assays using purified GRK2 and reconstituted inactive G protein heterotrimers could directly demonstrate this interaction in a controlled system, independent of other cellular factors. Alternatively, proximity-based assays such as BRET or FRET between GRK2 and Gβγ in ACKR4-expressing cells, under conditions that prevent G protein activation, could provide in-cell evidence of this novel complex. Incorporating such experiments would transform this fascinating hypothesis into a robustly supported conclusion, significantly increasing the impact of the study.

2. The manuscript's interpretation of the GRK reconstitution experiments presents a critical contradiction that requires clarification. The data in Figure 6h strongly position GRK2 as the primary kinase for chemokine scavenging, as its sole re-expression in GRK-deficient cells significantly restores CCL19 uptake. In contrast, the discussion states that "reconstitution of a single GRK... did not rescue the ability of ACKR4 to recruit β-arrestins" and speculates that "all four GRKs collectively regulate ACKR4 trafficking". This discrepancy raises a crucial question: By what mechanism does GRK2 rescue chemokine uptake if it fails to restore the β-arrestin recruitment pathway seemingly required for this function? The authors must explicitly address whether this implies one of two possibilities. First, that a sub-maximal level of β-arrestin recruitment, driven by GRK2 alone, is sufficient to promote significant chemokine uptake. Alternatively, this could be strong evidence for a distinct, β-arrestin-independent uptake mechanism that is specifically and potently enhanced by GRK2-mediated phosphorylation, a concept supported by data showing that uptake persists in cells lacking β-arrestins.

3. While the identification of Ser349 as a dually-regulated phosphorylation site is a key advance, its specific functional role remains inferred rather than directly demonstrated. The current evidence relies on a rescue experiment within a Cluster 3 mutant, which does not isolate the contribution of Ser349 in the wild-type context. To establish a direct causative link between this residue and its proposed functions, the authors should generate a single point mutant, ACKR4 S349A, and assess its impact on both CCL19 uptake and ligand-independent internalization. This crucial experiment would directly test the proposed dual role of Ser349 in steady-state and signal-induced pathways, substantially strengthening the manuscript's conclusions.

4. While the mechanistic insights from the HEK293 system are valuable, the study's conclusions rely exclusively on a heterologous overexpression model. The physiological relevance of these findings, particularly the central role of GRK2/3 in scavenging, remains unconfirmed in a native context, such as that of lymphatic endothelium where ACKR4 is functionally important. To address this, the authors should validate their primary conclusion in a cell type that endogenously expresses ACKR4. A crucial experiment would be to use siRNA to knockdown GRK2/3 and measure the effect on endogenous CCL19 scavenging. Confirming that this recapitulates the profound uptake impairment seen in the knockout system would substantially strengthen the study's impact by demonstrating that the proposed GRK hierarchy is a feature of ACKR4's native function, not an artifact of the expression system.

Minor Issues
1. The legend for Figure 6 appears to contain errors. The description for panel f, which depicts β-arrestin2 recruitment, is missing.

2. The manuscript could be strengthened by refining the phrasing to better reflect the data. In the description of Figure 1a, the claim that ACKR4 is "more sensitive" should be substantiated by calculating and reporting the EC50 values. Similarly, the description of the Cluster 1 mutant's activity in Figure 3h ("more... was internalised") should be softened to reflect the non-significant p-value ($p=0.0673$), for instance, by stating that it "showed a tendency towards increased internalization."

3. The misspelling "heterotrimieric" should be corrected to "heterotrimeric". In the Figure 4 legend, the concentration "1 M CCL19" is likely a typo and should probably be 1 µM CCL19.

Reviewer #2

(Remarks to the Author)
The study presented by Gerken et. al in this manuscript described the phosphorylation of ACKR4 and its functional role in β-arrestin recruitment, chemokine internalization, and receptor trafficking. Combining with the indirect BRET assay, mutagenesis and genetic knockout approaches, the authors revealed the G protein-independent regulation of ACKR4 and proposed a phosphorylation-dependent mechanism for its activation. The results connect these functional outputs to the actions of particular GRKs, thereby offering insight into how ACKR4 activity is differentially regulated. However, several conclusions or statements made throughout the investigation appear inconsistent with the observed results (see points below). Further investigations are required to establish a more detailed and clearer understanding of the role of GRK-mediated phosphorylation in the function of ACKR4.

Major points
- In page 7, line 185-187, the authors concluded that the cluster 2, cluster3 and ST/A mutants attenuate chemokine uptake. Given the lower surface expression levels of these mutants compared to the wild-type receptor, particularly for the ST/A

mutant, it is necessary to elucidate the impact of surface expression on chemokine internalization. Otherwise it would be difficult to make a link to receptor phosphorylation.
- In page 7, line 202-207, it was concluded that the cluster mutants also affect ligand-independent receptor internalization. However the observed differences were marginal, making it difficult to support such a conclusion.
- In page 8, line 202-209, Cluster 1 residues appear particularly involved in endocytosis to early endosomes. However, it is unclear whether CCL19 stimulation was used to assess ligand-dependent internalization. This experiment would be important to distinguish cluster-specific contributions under basal versus stimulated conditions.
- In page 9, line 226-228, the attenuation effect of all cluster mutants on the basal signal across GRKs was relatively modest (less than 20%), making it difficult to interpret their differential impact on GRK recruitment.
- In page 11, line 268-270, the G proteins were concluded not required for GRK2/3 recruitment to ACKR4, however, the R587Q mutant does affect GRK2 recruitment. What is the role of Gβγ? It seems the G protein KO cells exhibit slightly weaker arrestin 1 recruitment, with a reduction of around 30%. Further discussion is needed to clarify this observation and substantiate the conclusion.
- In Fig. 6c and 6d, knockout of GRKs significantly attenuates both the total expression and surface levels of AKCR4. How does it affect the assessment of protein recruitment using bystander BRET approach?
- In Fig 6e and 6f, CCL19-driven recruitment of β-arrestin to the plasma membrane is clearly reduced in the GRK knockout cells, highlighting a role for GRKs in ligand-induced arrestin engagement. Could the authors also clarify whether basal (pre-associated) β-arrestin interactions are altered in these GRK-deficient cells? Please clarify whether GRKs influence only the ligand-dependent recruitment of arrestin or also contribute to its basal association with ACKR4.
- In Fig. 6h, expression of GRK2 in GRK KO cells appears to improve ligand uptake. However, one of the three data points is markedly higher than the others, disproportionately increasing the average MFI ratio. The confidence in this result should be clarified, and it would be important to know whether additional replicates were performed to validate the observation.
- In Fig. 7, the mass spectrometry analysis revealed the phosphorylation of Ser349. Given the proximity of this phosphosite to others, it is likely that the identified phosphopeptides in this region contain multiple potential phosphorylation sites. How confident is the conclusion that only Ser349 is phosphorylated? I could not find details of the identified peptides of ACKR4, as well as their fragmentation spectra to support Ser349 phosphorylation. Considering the phosphorylation at Ser338/T342 was identified via a site-specific antibody, it is important for the authors to clarify why these sites were not detected in the LC–MS analysis. Moreover, the details of the quantification approach are lacking, which makes data interpretation challenging. The authors should provide more information on their mass spectrometry analysis and statistical methods to make the conclusions more convincing.

Minor points
- An illustration of the experimental design for the bystander BRET assay would be helpful for clarity.
- The description of chemokine production in the Methods section is very brief and should include more detailed information on the protein production procedure.
- In Fig. 7a, ACKR4 was identified in the volcano plot as upregulated. However, it should not appear in the control group. How was the ratio quantified?
- Certain sections lack clarity due to loosely structured context. Providing more concise explanations would enhance overall readability.
- In several figures (e.g., Fig. 1e, Fig. 2d, Fig. 3f), the X-axis labels are not centered under the corresponding bars. For consistency and readability, it would be preferable to align the labels with the bars.

Reviewer #3

(Remarks to the Author)
In this paper, Gerken et al. studied the interaction of ACKR4 with GRKs and arrestins and its functional consequences. By generating different clusters of phosphorylation-site mutants, they examined how individual phosphorylation sites affect arrestin coupling, ligand uptake, and GRK recruitment. The authors also used mass spectrometry to probe the phosphorylation status of ACKR4 and its interaction patterns. The topic is interesting; however, many of the conclusions are not comprehensive. I also have concerns regarding the GRK recruitment assays.
This study focuses on both steady-state and stimulated conditions, but the experimental definition of “steady state” is not provided. For the BRET and NanoBiT assays, only AUC values are shown in bar graphs. It would be important to present the raw time curves as well, since many results rely on different basal levels and changes before and after stimulation.
Among the phosphorylation clusters, cluster 1 is particularly interesting, as it exhibits opposite effects on receptor surface expression and CCL19 uptake compared to the ST/A mutant. Additionally, it shows increased basal interaction with both arrestins and enhanced ligand-independent receptor internalization. The authors, however, did not propose any possible explanations for these findings.
I also have reservations about the GRK recruitment assays. According to the authors, phosphorylation sites are critical for GRK2/3 and GRK6 recruitment, since mutating all of them abolishes most interactions; therefore, the ST/A mutant was used as an internal basal control (Fig. 4a). However, the prevailing model in the field is that GRKs are recruited to the membrane by the activated GPCR core through their N-terminal helix, not by the C-terminal phosphorylation sites. Indeed, a recent study on ACKR3 phosphorylation (PMID: 40399676) showed that blocking the activated core inhibited GRK2/GRK5 phosphorylation. The GPCR C-tail is flexible, accessible to GRKs, and does not undergo major conformational changes upon activation. If phosphorylation sites were the main determinants for ACKR4–GRK interaction, GRKs should be constitutively recruited to the membrane, since the sites are present in the basal state.
At line 223, the authors claim that they did not detect GRK5 recruitment because GRK5 directly interacts with the plasma membrane through a C-terminal amphipathic helix. However, this helix is conserved in the GRK4 subfamily, which includes GRK4, GRK5, and GRK6. GRK6 also contains a lipid modification that further promotes its membrane localization. It is very

surprising that the author could observe GRK6, but not GRK5, recruitment. A more reasonable explanation and more experimental validation is necessary.
GRK recruitment is known to activate kinase activity, but it is not clear that kinase activity in turn regulates GPCR coupling. It is interesting that the authors observe decreased ACKR4 binding with kinase-dead GRKs. Since GRK recruitment assays, especially for GRK5/6, are notoriously challenging, the raw data should be shown, and these observations should be validated with additional approaches.
The authors also claim that "GRK2/3 recruitment occurs independent of G protein activation," but this contradicts their results (Fig. 5f,g). The R587Q GRK2 mutant shows a ~50% reduction in AUC ΔBRET, indicating that GRK2 membrane recruitment is at least partly dependent on Gβγ. This undermines the claim of G protein–independent recruitment. The statement "Steady-state interaction of GRK2 R587Q with CAAX and its recruitment were both reduced, but still intact (Figure 5g)" is unclear. A 50% reduction is not trivial. If the authors propose that free Gβγ arises from sources other than G protein activation, this needs to be explicitly stated.
The phosphorylation-site identification is also unsatisfactory. The antibody-based detection of pS338/pT342-ACKR4 provides very limited information. In fact, mutating these two residues (cluster 2) has little effect on CCL19 uptake or arrestin recruitment. Thus, this assay contributes little to the overall story. As noted in lines 279–281, the only solid conclusion is that S338 and T342 can be phosphorylated; no statements should be made about the full receptor, as other sites may also be phosphorylated.
The mass spectrometry analysis is likewise limited. Despite extensive analysis, only one phosphorylation site was identified. It is well established that GRKs typically phosphorylate GPCRs at multiple sites. Indeed, the authors themselves show in Fig. 6a,b that both S338 and T342 can be phosphorylated. The significance of identifying just one site is questionable. The conclusion that a single phosphorylation site can account for increased GRK2/3 coupling is not convincing, particularly in light of the concerns noted above.

Reviewer #4

(Remarks to the Author)
I co-reviewed this manuscript with one of the reviewers who provided the listed reports. This is part of the Nature Communications initiative to facilitate training in peer review and to provide appropriate recognition for Early Career Researchers who co-review manuscripts.

Version 1:

Reviewer comments:

Reviewer #1

(Remarks to the Author)
I thank the authors for their careful assessment and diligent performance of the additional experiments. The revised manuscript has been significantly improved by the inclusion of key data, such as the BRET/Co-IP assays and the validation in native fibroblasts. These additions have successfully addressed all my previous concerns. I am satisfied with the current version and believe it now meets the high standards for publication.

Reviewer #2

(Remarks to the Author)
The authors have appropriately responded to the majority of our previous comments, and I appreciate the additional experiments conducted to address the key points raised. The inclusion of data from human fibroblasts with endogenously expressed ACKR4 provides valuable support for the role of GRK2/3 in chemokine uptake and significantly strengthens the physiological relevance of the study. However, despite these improvements, I remain concerned regarding specific data interpretations, particularly in Figure 7h. While the authors attempted to address the potential outlier using various statistical tests (Two-way ANOVA, Spearman's test, and Shapiro-Wilk), these methods are statistically inappropriate for identifying outliers in a small sample size (n=3). To clarify that Two-way ANOVA compares group means rather than individual data points; Spearman's test assesses correlation between variables; and the Shapiro-Wilk test lacks sufficient power to determine normality in such a limited dataset. Consequently, the statistical justification provided is invalid. Instead of emphasizing a distinct effect of GRK2 in enhancing CCL19 uptake based on this data, I would suggest the authors modify their statement to reflect a more neutral interpretation. Additionally, while ACKR4 phosphorylation and interaction patterns are emphasized throughout a significant portion of the manuscript, the specific functional relevance of the three phosphorylation sites (Ser338, Thr342, and Ser349) remains somewhat ambiguous. I suggest the authors explicitly discuss this aspect in the Discussion section. It would be beneficial to acknowledge the limitations of antibody-based detection and contextualize how these findings fit within the broader, and often heterogeneous, phosphorylation landscape of GPCRs.

Reviewer #3

(Remarks to the Author)
I have strong reservations regarding the bystander BRET assay, particularly after examining the original data plots provided by the authors in their response. For GRK5 and GRK6, the bystander BRET approach is not appropriate. GRK5/6 are predominantly localized at the plasma membrane under basal conditions; therefore, there is no cytosol-to-membrane translocation to be monitored by this assay. Consistent with this, the authors' original data show a decrease in BRET signal

rather than the typical increase observed for GRK2/3 and arrestins. This decrease in BRET signal could result from a combination of GRK5/6 trafficking dynamics and competition with endogenous GRK5/6, and thus does not directly reflect a specific interaction between ACKR4 and GRK5/6. Consequently, I do not believe that conclusions drawn from this assay for GRK5/6 are reliable.

Along the same lines, the GRK2 response appears very subtle. The most convincing interaction observed is between GRK3 and ACKR4. I strongly recommend that the authors present the full time-course data alongside the AUC analysis and clearly indicate how the AUC was calculated. For example, based on P2P Fig. 4h, I do not observe a significant difference between GRK6 WT and the kinase-dead variant.

Reviewer #4

(Remarks to the Author)
I co-reviewed this manuscript with one of the reviewers who provided the listed reports. This is part of the Nature Communications initiative to facilitate training in peer review and to provide appropriate recognition for Early Career Researchers who co-review manuscripts.

Version 2:

Reviewer comments:

Reviewer #2

(Remarks to the Author)
The authors have addressed the statistical questions and concerns regarding the characterization of the ACKR4 phosphorylation. By incorporating both the mass spectrometry and antibody-based data in the Discussion, they provide the necessary context to highlight the potential heterogeneity of phosphorylation observed across different techniques. We have no further points to raise on the current version of the manuscript.

Reviewer #3

(Remarks to the Author)
The authors have addressed my concerns.

Reviewer #4

(Remarks to the Author)
I co-reviewed this manuscript with one of the reviewers who provided the listed reports. This is part of the Nature Communications initiative to facilitate training in peer review and to provide appropriate recognition for Early Career Researchers who co-review manuscripts.

Open Access This Peer Review File is licensed under a Creative Commons Attribution 4.0 International License, which permits use, sharing, adaptation, distribution and reproduction in any medium or format, as long as you give appropriate credit to the original author(s) and the source, provide a link to the Creative Commons license, and indicate if changes were made.
In cases where reviewers are anonymous, credit should be given to 'Anonymous Referee' and the source.
The images or other third party material in this Peer Review File are included in the article's Creative Commons license, unless indicated otherwise in a credit line to the material. If material is not included in the article's Creative Commons license and your intended use is not permitted by statutory regulation or exceeds the permitted use, you will need to obtain permission directly from the copyright holder.
To view a copy of this license, visit https://creativecommons.org/licenses/by/4.0/

REVIEWER COMMENTS

Reviewer #1 *(Remarks to the Author):*

This is a well-designed and clearly written manuscript by Gerken et al. that investigates the complex regulation of the atypical chemokine receptor 4 (ACKR4) by G protein-coupled receptor kinases (GRKs). The authors use a comprehensive and technically sound experimental approach, including β-arrestin recruitment assays, chemokine uptake measurements, GRK knockout and rescue experiments, and mass spectrometry-based phosphorylation analysis. The central advance of this work is the meticulous dissection of how the four non-visual GRKs (2, 3, 5, and 6) differentially regulate ACKR4's chemokine scavenging function. A key strength of the study is the use of native, untagged ACKR4, which circumvents artefacts associated with C-terminal tags that the authors' own previous work identified. Notably, the authors convincingly demonstrate that ACKR4 constitutively cycles and associates with β-arrestins. They identify distinct C-terminal serine/threonine clusters that are critical for steady-state trafficking and ligand-induced internalization. The study provides compelling evidence for a division of labor among GRKs, with GRK5/6 primarily regulating steady-state phosphorylation and GRK2/3 being recruited upon ligand stimulation to enhance chemokine uptake. Most notably, the authors suggest a paradigm-challenging finding that GRK2/3 recruitment to ACKR4 occurs independently of G protein activation.

We thank the Reviewer for the overall very positive feedback. We are pleased to read that the referee considers our study as well-designed, clearly written, comprehensive, compelling, and technically sound. We thank the Reviewer for emphasizing the central advances of our work and for highlighting the use of native, untagged ACKR4 as key strength of our study.

While the paper presents a thorough and detailed molecular dissection of ACKR4, a critique could argue that several of its core findings confirm mechanisms that were already well-established for its more famous family member, ACKR3. Here are points where the novelty could be considered limited, particularly in the context of existing ACKR3 literature.
First, the paper emphasizes that GRK2 and GRK3 are recruited to ACKR4 without any G protein activation, framing this as a challenge to the existing paradigm. However, this G protein-independent, β-arrestin-biased signaling is the defining characteristic of the entire ACKR family, with ACKR3 being the archetypal example. ACKR3 is famously known to recruit GRKs and signal through β-arrestin without coupling to G proteins. Therefore, demonstrating this same core mechanism in ACKR4 is more of a confirmation of a shared family trait rather than a fundamentally new discovery.

We thank the Reviewer for pointing out insights on ACKR3, the atypical chemokine receptor that has been studied the most so far. We agree that the G protein-independent, βarrestin-biased signalling is indeed the defining characteristic of the ACKR family (except for ACKR1, which does not interact with βarrestins). ACKR3 has indeed been shown to be phosphorylated by GRK5 and GRK2 [refs. 36, 37 of our manuscript]. While it is agreed that ACKR3 recruits GRKs and βarrestins without directly coupling to G proteins, the mechanism by which GRK2 can phosphorylate ACKR3 upon CXCL12 stimulation remains unclear. Canonical GPCR signalling has revealed that GRK2 recruitment to activated receptors is linked to heterotrimeric G protein activation as liberated Gβγ facilitates the translocation of GRK2 to the membrane through interaction with the C-terminal PH domain of the kinase [refs. 6, 10]. As ACKR3 does not activate G proteins, there is no free Gβγ released by heterotrimeric G protein activation. Therefore, it remains to be determined how CXCL12 recruits GRK2 to ACKR3 to facilitate its phosphorylation. Ref. 36 provides clear evidence that GRK2 indeed phosphorylates ACKR3 in a G protein-independent manner but does not provide information on the underlying mechanism. Notably, two recent studies [refs. 37, 38]

have proposed an indirect cooperative mechanism, whereby CXCL12 co-activates its canonical, ubiquitously expressed receptor CXCR4. This is proposed to provide sufficient free Gβγ to promote GRK2-mediated ACKR3 phosphorylation. While this scenario is plausible for the ACKR3/CXCR4 axis, it cannot be a core mechanism common to the ACKR family, as we are not aware of any cell that would simultaneously express the classical (CCR7) and the atypical (ACKR4) receptor for CCL19. In the present study, we provide experimental evidence that (apo) ACKR4 can form a complex with the G protein and GRK2 (**new Figure 6**), and that ligand stimulation induces receptor phosphorylation by GRKs independently of G protein activation. To the best of our knowledge, our study is the first to propose a mechanism by which GRK2/3 enable direct G protein independent receptor phosphorylation. Our mechanistic insight may be transferrable to the entire family of scavenging atypical chemokine receptors.

We have substantially revised the discussion to provide a clearer description of the unifying characteristics of the ACKR family and the atypical features of its members.

Second, the paper establishes that ACKR4 constitutively associates with β-arrestins and cycles between the plasma membrane and endosomes, even in its apo (ligand-free) state. Again, ACKR3 is well-known for its high level of constitutive activity and β-arrestin interaction, which drives its continuous trafficking. The authors' use of a new assay to study the untagged receptor is a methodological advance, but the underlying biological concept of constitutive activity was already a known feature of this receptor subfamily.
The authors may overstate the novelty of their findings for ACKR4 by not sufficiently comparing them with the well-established mechanisms of ACKR3. Since ACKRs have been delineated from GPCRs based on shared characteristics—such as their inability to couple to G proteins—it is important that the data is analyzed and discussed within this shared framework. While the experiments are robust and the data generally support the conclusions, several key areas in the interpretation and discussion could be strengthened to further solidify the manuscript's impact.
In this context, the authors may overstate the novelty of their findings for ACKR4 by not sufficiently comparing them with the well-established mechanisms of ACKR3. The discussion should be revised to contextualize the results within the broader ACKR family, which would ultimately strengthen the manuscript's conclusions. Since ACKRs have been delineated from GPCRs based on shared characteristics—such as their inability to couple to G proteins—it is important that the data is analyzed and discussed within this shared framework.

We agree with the Reviewer. We have added shared characteristics in the introduction (lanes 99-100) and have substantially revised the entire discussion to contextualize our results within the broader ACKR family, including ACKR3.

Overall, while the experiments are robust and the data generally support the conclusions, several major issues should be addressed to fully solidify the manuscript's claims.
Major issues
1. The conclusion that GRK2 interacts with the inactive heterotrimeric G protein is one of the most provocative and potentially impactful claims in the manuscript. However, this conclusion currently rests on limited and indirect experimental support, primarily the partial reduction in recruitment observed with the GRK2 R587Q mutant. While suggestive, this single piece of data is not sufficient to rule out alternative interpretations, such as non-specific effects of the mutation on protein stability or membrane affinity. To substantiate this paradigm-shifting claim, further and more direct experimental evidence is required. For instance, the authors could perform co-immunoprecipitation experiments, pulling down ACKR4 and probing for the presence of both GRK2 and inactive G protein subunits (e.g., in the presence of GDP) to test for a ternary complex. Furthermore, in vitro binding assays using purified GRK2 and reconstituted inactive G protein heterotrimers could directly demonstrate this interaction in a controlled system, independent of other cellular factors. Alternatively, proximity-based assays such as BRET or FRET between GRK2 and Gβγ in ACKR4-expressing cells, under

conditions that prevent G protein activation, could provide in-cell evidence of this novel complex. Incorporating such experiments would transform this fascinating hypothesis into a robustly supported conclusion, significantly increasing the impact of the study.

We thank the Reviewer for highlighting the potential impact and for proposing additional experiments to solidify our conclusions. As recommended, we performed co-immunoprecipitation experiments to test for ternary complexes formed between ACKR4, GRK2 and the G protein. To this end, we transfected HEK293 cells with constructs coding for HA-ACKR4, FLAG-GRK2, and the trimeric Gβγ-cpVenus/$G\alpha_{i1}$ complex, followed by immunoprecipitation of HA-ACKR4 using an anti-HA antibody. As shown in a **new Figure 6i**, we detected co-immunoprecipitated FLAG-tagged GRK2 and cpVenus-tagged Gγ in HA-ACKR4 pulldown experiments, indicating that ACKR4, GRK2 and Gβγ indeed form a ternary complex.

As we do not have purified GRK2 and reconstituted inactive G protein heterotrimers at hand, we opted for the proposed alternative approach and used a series of proximity-based BRET assays to substantiate our findings. First, we measured changes in ΔBRET ratios between GRK2 and Gβγ in response to CCL19 stimulation in HEK293 cells co-expressing GRK2-nLuc, Gβγ-cpVenus/$G\alpha_{i1}$ together with either ACKR4, CCR7, or ACKR3 (serving as negative control). As shown in a **new Figure 6a and supplementary Figure S3a**, we found enhanced ΔBRET signals between GRK2 and Gβγ upon CCL19 stimulation in cells expressing either ACKR4 or CCR7. As expected, no changes in ΔBRET were observed in cells expressing ACKR3. Notably, no increase in ΔBRET was detected if GRK2 R587Q was used instead of wild-type GRK2. Moreover, in the absence of CCL19 stimulation, the basal BRET signal was higher between GRK2 and Gβγ as compared to GRK2 R587Q and Gβγ in cells expressing ACKR4 (or CCR7) (**new Figure 6b**). Similar results were obtained for GRK3 and Gβγ (**new Figure 6c-d and supplementary Figure S3b**). As there is no equivalent GRK2 R587Q mutant for GRK3, we could not perform the corresponding negative control experiment. Finally, we tested whether overexpression of the heterotrimeric G protein could enhance the association of GRK2 and GRK3 with CAAX upon CCL19 stimulation of ACKR4. As shown in a **new Figure 6e-h and supplementary Figure S3c-d**, overexpression of the G protein increased both the steady-state association with and the CCL19-mediated recruitment of GRK2/3 to the plasma membrane. The finding that CCL19-mediated GRK2/3 recruitment to ACKR4 proximal CAAX is independent of G protein activation is depicted in **Figure 5d-e**, where it is shown to occur in HEK293 cells lacking functional G proteins (i.e. in PTX treated $\Delta G\alpha_{q/12/s}$ cells).

Collectively, our co-immunoprecipitation and proximity-based experiments corroborate our conclusion that GRK2 interacts with the G protein and form a ternary complex with ACKR4 in its ligand-free state.

2. The manuscript's interpretation of the GRK reconstitution experiments presents a critical contradiction that requires clarification. The data in Figure 6h strongly position GRK2 as the primary kinase for chemokine scavenging, as its sole re-expression in GRK-deficient cells significantly restores CCL19 uptake. In contrast, the discussion states that "reconstitution of a single GRK... did not rescue the ability of ACKR4 to recruit β-arrestins" and speculates that "all four GRKs collectively regulate ACKR4 trafficking". This discrepancy raises a crucial question: By what mechanism does GRK2 rescue chemokine uptake if it fails to restore the β-arrestin recruitment pathway seemingly required for this function? The authors must explicitly address whether this implies one of two possibilities. First, that a sub-maximal level of β-arrestin recruitment, driven by GRK2 alone, is sufficient to promote significant chemokine uptake. Alternatively, this could be strong evidence for a distinct,

β-arrestin-independent uptake mechanism that is specifically and potently enhanced by GRK2-mediated phosphorylation, a concept supported by data showing that uptake persists in cells lacking β-arrestins.

We thank the Reviewer for raising this question. In fact, ACKR4 and ACKR3 have been shown to take up chemokines in cells lacking βarrestins (refs. 32, 35, 36). Notably, chemokine scavenging by ACKRs is reduced but not fully abrogated in cells lacking βarrestin1/2, demonstrating that βarrestins are involved but dispensable for chemokine scavenging. Our data on GRK2 are also in line with a previous report showing that ACKR3 functions require GRK-mediated phosphorylation but not βarrestins (ref.36). This is also supported by the finding that the ST/A mutants of ACKR3 (ref 36) and ACKR4 (Figure 2) do not recruit βarrestins but take up chemokines albeit less efficient as wild-type receptors. We have substantially revised the discussion to clarify the roles of GRKs and βarrestins in chemokine uptake by ACKRs.

3. While the identification of Ser349 as a dually-regulated phosphorylation site is a key advance, its specific functional role remains inferred rather than directly demonstrated. The current evidence relies on a rescue experiment within a Cluster 3 mutant, which does not isolate the contribution of Ser349 in the wild-type context. To establish a direct causative link between this residue and its proposed functions, the authors should generate a single point mutant, ACKR4 S349A, and assess its impact on both CCL19 uptake and ligand-independent internalization. This crucial experiment would directly test the proposed dual role of Ser349 in steady-state and signal-induced pathways, substantially strengthening the manuscript's conclusions.

As requested, we generated the single point mutant ACKR4 S349A and assessed its impact on both CCL19 uptake and its ligand-independent internalization. Unexpectedly, ACKR4 S349A was slightly better in taking up CCL19-S6^{Dy649P1} and in ligand-independent internalization than wild-type ACKR4 (**P2P Figure 1**). To further analyse this single point mutation, we assessed the steady-state association with as well as the chemokine-mediated recruitment of βarrestins to the plasma membrane using the bystander split-luciferase assay. We observed a substantially higher pre-association of βarrestin1 and βarrestin2 with the plasma membrane under steady-state conditions in cells expressing ACKR4 S349A as compared to cells expressing wild-type ACKR4, whereas the CCL19-driven recruitment of both βarrestins to ACKR4 S349A and wild-type ACKR4 was comparable. Notably, Ser349 is one of the four amino acids located at the tip of ACKR4 forming the consensus sequence (-T-F-S_{349}-I) of a putative class II PDZ-binding domain (-X-Φ-X-Φ). Interestingly, ACKR4 S349A behaves similarly to ACKR4 I350A and ACKR4 F348A with regards to enhanced chemokine uptake and βarrestin pre-association with the plasma membrane (see Gerken, et al. doi: 10.1186/s12964-024-01961-8). Thus, it seems that mutating single residues at the tip of ACKR4, either S349 or its direct neighbours, similarly affect chemokine uptake and receptor internalization. However, because the PDZ protein involved in ACKR4 trafficking has not yet been identified, any conclusions would be speculative. We cannot draw a definitive conclusion about receptor phosphorylation based on this particular mutation. However, re-introducing S349 in the Cluster 3 mutant enhanced GRK2 and GRK3 recruitment in response to CCL19 stimulation as shown in **Figure 8e** of the revised manuscript.

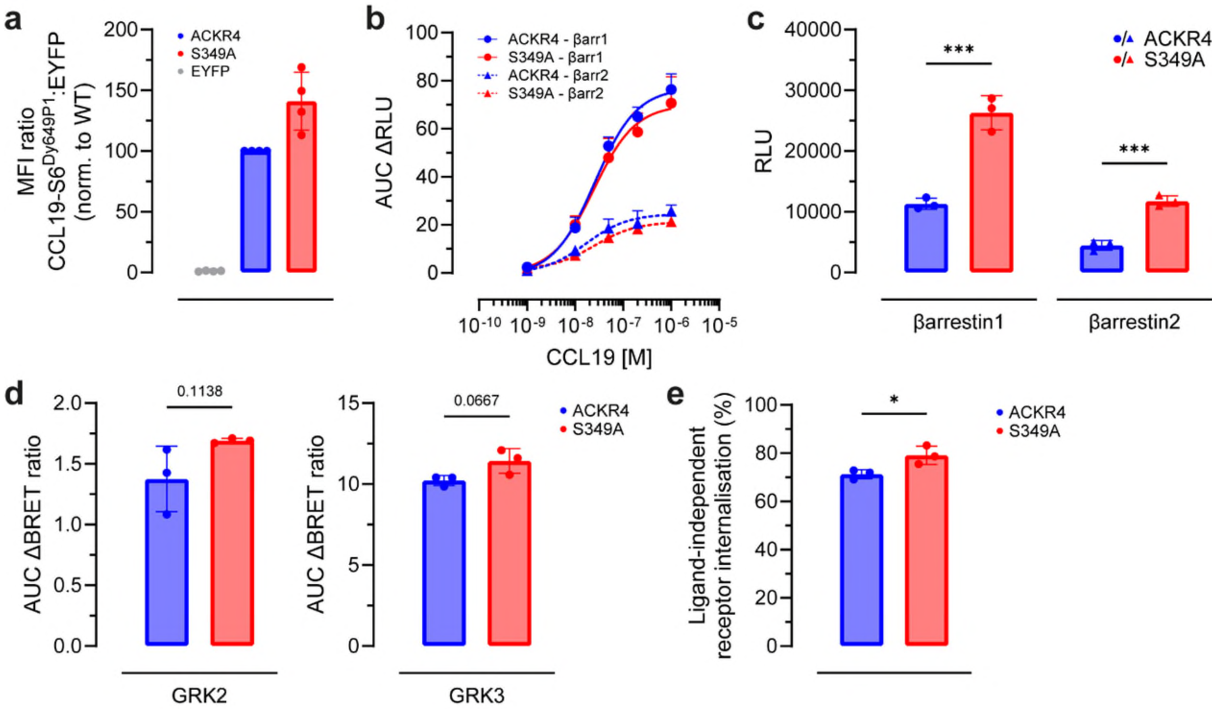

P2P Figure 1: Single mutation of Ser349 enhances chemokine uptake and receptor cycling. a Chemokine uptake (5 nM CCL19 for 30 min) in HEK293 cells transiently expressing either EYFP only, EYFP/ACKR4 or EYFP/ACKR4 S349A; n = 4, mean ± SD. **b** Bystander βarrestin1/2 recruitment to ACKR4 or ACKR4 S349A upon stimulation with graded concentrations of CCL19, and **c** corresponding basal interaction of βarrestin1/2 with the plasma membrane in the absence of ligand; n = 3; mean ± SD. **d** Indirect GRK2 and GRK3 recruitment to ACKR4 or ACKR4 S349A upon CCL19 stimulation (1 µM); n = 3; mean ± SD. **e** Ligand-independent receptor internalisation in HEK293 cells transiently transfected with either EYFP/ACKR4 or EYFP/ACKR4 S349A; n = 3, mean ± SD. Statistical analysis: **c d e** unpaired t test; * $p < 0.05$; *** $p < 0.001$.

4. While the mechanistic insights from the HEK293 system are valuable, the study's conclusions rely exclusively on a heterologous overexpression model. The physiological relevance of these findings, particularly the central role of GRK2/3 in scavenging, remains unconfirmed in a native context, such as that of lymphatic endothelium where ACKR4 is functionally important. To address this, the authors should validate their primary conclusion in a cell type that endogenously expresses ACKR4. A crucial experiment would be to use siRNA to knockdown GRK2/3 and measure the effect on endogenous CCL19 scavenging. Confirming that this recapitulates the profound uptake impairment seen in the knockout system would substantially strengthen the study's impact by demonstrating that the proposed GRK hierarchy is a feature of ACKR4's native function, not an artifact of the expression system.

We fully agree with the Reviewer. We have chosen human BJ hTERT fibroblasts, which endogenously express ACKR4 and efficiently take up CCL19-S6^{Dy649P1} (**new supplementary Figure S7**). Knocking down of GRK2 and GRK3 by siRNA in BJ hTERT fibroblasts significantly reduced CCL19-S6^{Dy649P1} uptake which was in line with our findings in HEK293 ΔGRK2/3 cells. Moreover, treating BJ hTERT fibroblasts with cmpd101, the small molecule inhibitor of GRK2/3, similarly reduced the uptake of CCL19-S6^{Dy649P1} by endogenous ACKR4. These additional data are depicted in a **new Figure 8f-g** and confirm the role of GRK2/3 in chemokine uptake in human fibroblasts that endogenously express ACKR4.

Minor Issues

1. The legend for Figure 6 appears to contain errors. The description for panel f, which depicts β-arrestin2 recruitment, is missing.

We thank the Reviewer for spotting the errors. We have corrected the errors and added the description for panel f. Note that the former Figure 6 is now Figure 7.

2. The manuscript could be strengthened by refining the phrasing to better reflect the data. In the description of Figure 1a, the claim that ACKR4 is "more sensitive" should be substantiated by calculating and reporting the EC50 values. Similarly, the description of the Cluster 1 mutant's activity in Figure 3h ("more... was internalised") should be softened to reflect the non-significant p-value (p=0.0673), for instance, by stating that it "showed a tendency towards increased internalization."

We thank the Reviewer and have added the corresponding EC50 values in the description of Figure 1a. The indicated non-significant p-value indicated by the Reviewer refers to the ACKR4 ST/A mutant and not to the Cluster 1 mutant. The difference in internalisation between ACKR4 and its Cluster 1 mutant is statistically significant (p=0.042; *), thus there is no need to amend the corresponding description.

3. The misspelling "heterotrimieric" should be corrected to "heterotrimeric". In the Figure 4 legend, the concentration "1 M CCL19" is likely a typo and should probably be 1 µM CCL19.

We thank the reviewer for pointing out the misspelling, which has now been corrected. We indeed used 1 µM CCL19 for the experiments shown in Figure 4 as indicated in the legend.

Reviewer #2 (Remarks to the Author):

The study presented by Gerken et. al in this manuscript described the phosphorylation of ACKR4 and its functional role in β-arrestin recruitment, chemokine internalization, and receptor trafficking. Combining with the indirect BRET assay, mutagenesis and genetic knockout approaches, the authors revealed the G protein-independent regulation of ACKR4 and proposed a phosphorylation-dependent mechanism for its activation. The results connect these functional outputs to the actions of particular GRKs, thereby offering insight into how ACKR4 activity is differentially regulated. However, several conclusions or statements made throughout the investigation appear inconsistent with the observed results (see points below). Further investigations are required to establish a more detailed and clearer understanding of the role of GRK-mediated phosphorylation in the function of ACKR4.

We thank the reviewer for the careful assessment of our study and for the constructive feedback, which has helped us to improve our manuscript. We have performed a series of additional experiments to establish a more detailed and clearer understanding of the role of GRK-mediated phosphorylation in the function of ACKR4.

Major points

- In page 7, line 185-187, the authors concluded that the cluster 2, cluster3 and ST/A mutants attenuate chemokine uptake. Given the lower surface expression levels of these mutants compared to the wild-type receptor, particularly for the ST/A mutant, it is necessary to elucidate the impact of surface expression on chemokine internalization. Otherwise it would be difficult to make a link to receptor phosphorylation.

We thank the reviewer for emphasising the importance to elucidate the impact of receptor surface expression on CCL19 uptake. As shown in **Figure 3b**, ACKR4 surface expression of wild-type, Cluster 2 and Cluster 3 mutants are comparable and do not differ significantly. Likewise, CCL19 uptake by wild-type ACKR4 and Cluster 2 mutant is equal (**Figure 3c**). In contrast, Cluster 1 mutant is superior in CCL19 internalisation as compared to wild-type receptor, despite lower surface expression, which is explained by the higher βarrestin pre-association (**Figure 3f**). Notably, receptor surface expression and CCL19 uptake shown in Figure 3b,c have been normalized to the total expression level of the receptor. Thus, only the ST/A mutant shows lower surface expression and reduced CCL19 internalisation, and we did not assess receptor phosphorylation for the ST/A mutant. The description of the mutants in terms of surface expression and chemokine uptake is correct.

- In page 7, line 202-207, it was concluded that the cluster mutants also affect ligand-independent receptor internalization. However the observed differences were marginal, making it difficult to support such a conclusion.

We agree that the differences are marginal. In fact, only Cluster 1 shows statistically different ligand-independent internalisation compared to wild-type ACKR4. The revised sentence now reads “In support of our notion, slightly more Cluster 1 mutant was internalised in a ligand-independent manner as compared to wild-type ACKR4 and the other mutants (**Figure 3h**)”.

- In page 8, line 202-209, Cluster 1 residues appear particularly involved in endocytosis to early endosomes. However, it is unclear whether CCL19 stimulation was used to assess ligand-dependent internalization. This experiment would be important to distinguish cluster-specific contributions under basal versus stimulated conditions.

The corresponding paragraph refers to **Figure 3h** and assesses ligand-independent receptor internalisation, thus without CCL19 stimulation, as correctly described in the text. **Figure 3g** illustrates how ligand-independent internalisation of the receptor by antibody staining was determined.

As requested, we have performed the experiment in which we stimulated cells with CCL19 as shown in **P2P Figure 2**. However, we noted that the antibody recognition site, at least partially, overlapped with the chemokine binding site. We therefor do not include these data in the revised manuscript. However, we have used fluorescently labelled CCL19 to determine chemokine-mediated receptor internalisation as shown in **Figure 3c**.

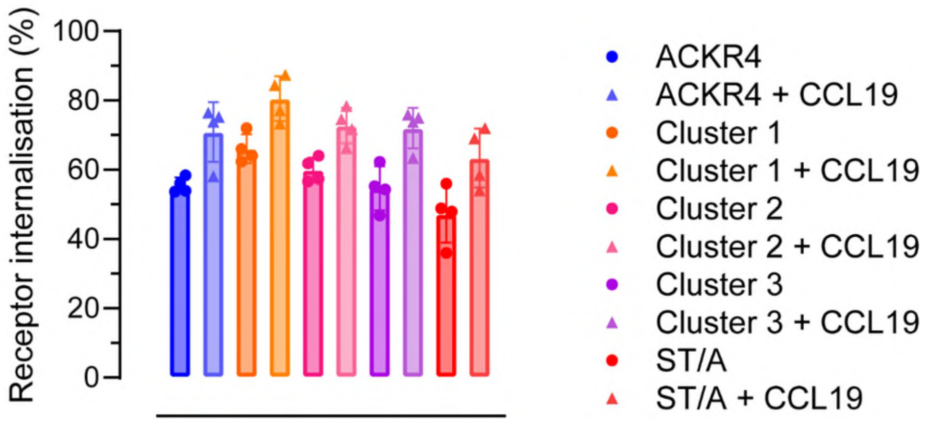

P2P Figure 2: Ligand-dependent and -independent receptor determined by antibody-staining. Cells expressing ACKR4 variants were incubated with the anti-ACKR4 antibody #362102, subsequently incubated in the presence (triangles) or absence (circles) of 100 nM CCL19, prior to staining using fluorescently labelled secondary antibody as illustrated in Figure 3g; n = 4, mean ± SD.

- In page 9, line 226-228, the attenuation effect of all cluster mutants on the basal signal across GRKs was relatively modest (less than 20%), making it difficult to interpret their differential impact on GRK recruitment.

We agree with the Reviewer that the basal, steady-state association of GRKs with the receptors at the plasma membrane is relatively modest, as expected. Nevertheless, the obtained information is important and serve as control for the chemokine-mediated recruitment of the GRKs to the membrane (**Figure 4a**) and the kinase-dead mutants (**Figure 4c**).

- In page 11, line 268-270, the G proteins were concluded not required for GRK2/3 recruitment to ACKR4, however, the R587Q mutant does affect GRK2 recruitment. What is the role of Gβγ? It seems the G protein KO cells exhibit slightly weaker arrestin 1 recruitment, with a reduction of around 30%. Further discussion is needed to clarify this observation and substantiate the conclusion.

We thank the Reviewer for this comment and take the occasion to emphasis the <u>G protein activation</u> is not required for GRK2/3 recruitment to ACKR4. In line with the additional experiments described in the point-by-point reply to the comments of **Reviewer 1** and depicted in a **new Figure 6**, we have conducted a series of additional experiments to corroborate the finding that ACKR4, GRK2 and the G protein (i.e. the γ subunit of the heterotrimeric protein) form a ternary complex in the absence of ligand stimulation and

without G protein activation. Hence, the Gβγ subunit of the G protein (not the free Gβγ released upon activation) facilitates GRK recruitment to ACKR4.

Indeed, βarrestin1 recruitment to ACKR4 was slightly weaker in $\Delta G\alpha_{q/12/s}$ cells, whereas in the same cells βarrestin2 recruitment to ACKR4 was slightly stronger (**Figure 5c**), and seem to compensate each.

- In Fig. 6c and 6d, knockout of GRKs significantly attenuates both the total expression and surface levels of AKCR4. How does it affect the assessment of protein recruitment using bystander BRET approach?

Protein recruitment using the bystander BRET approach are depicted as relative changes, i.e. as the ratio between CCL19 and PBS stimulated cells of the same origin. These relative changes obtained from one cell line allow the comparison with relative changes obtained from other samples. Although there may be differences in surface expression, the relative change in BRET ratio remains the same and does not affect the assessment.

- In Fig 6e and 6f, CCL19-driven recruitment of β-arrestin to the plasma membrane is clearly reduced in the GRK knockout cells, highlighting a role for GRKs in ligand-induced arrestin engagement. Could the authors also clarify whether basal (pre-associated) β-arrestin interactions are altered in these GRK-deficient cells? Please clarify whether GRKs influence only the ligand-dependent recruitment of arrestin or also contribute to its basal association with ACKR4.

The data on CCL19-driven recruitment of βarrestins to the plasma membrane are now shown in **Figure 7e,f** or the revised manuscript. The basal, pre-associated βarrestin is not significantly altered in GRK-deficient cells, which is in line with comparable βarrestin pre-association with CAAX in cells expressing ACKR4 and its ST/A mutant.

- In Fig. 6h, expression of GRK2 in GRK KO cells appears to improve ligand uptake. However, one of the three data points is markedly higher than the others, disproportionately increasing the average MFI ratio. The confidence in this result should be clarified, and it would be important to know whether additional replicates were performed to validate the observation.

This comment concerns now **Figure 7h** of the revised manuscript. We tested whether one of the data points is a significant outliner. We used 2-way ANOVA for statistical analysis. The data passed the Spearman's test for heteroscedasticity, which means that standard deviations are not significantly different. The data also passed the Shapiro-Wilk test assessing for normal distribution of data. These statistical tests reveal that there is no outlier present in our data. Hence, we have included all data points from all experiments.

- In Fig. 7, the mass spectrometry analysis revealed the phosphorylation of Ser349. Given the proximity of this phosphosite to others, it is likely that the identified phosphopeptides in this region contain multiple potential phosphorylation sites. How confident is the conclusion that only Ser349 is phosphorylated? I could not find details of the identified peptides of ACKR4, as well as their fragmentation spectra to support Ser349 phosphorylation. Considering the phosphorylation at Ser338/T342 was identified via a site-specific antibody, it is important for the authors to clarify why these sites were not detected in the LC–MS analysis. Moreover, the details of the quantification approach are lacking, which makes data interpretation challenging. The authors should provide more information on their mass spectrometry analysis and statistical methods to make the conclusions more convincing.

We thank the Reviewer for this comment. We apologise for not having provided sufficiently clear information of the mass spectrometry analysis. We are happy to provide additional information and clarification.

We now provide a **new supplementary Figure S6.** Panel **a** depicts the ACKR4 peptides identified by MS, including its post-translational modifications (oxidation and phosphorylation). Panel **b** provides the spectra and extracted ion current of the tryptic peptide containing pSer338. Panel **c** provides extracted ion current of the tryptic peptide containing pSer349, as well as two peptides of ACKR4 without post translational modifications.

The phosphorylation localization probability is calculated based on a published algorithm (Bekker-Jensen, et al., doi: 10.1038/s41467-020-14609-1). The whole data set was filtered before statistical analyses with a site localization probability for PTMs >75 % as now indicated in the method section. Multiple phosphorylation sites for ACKR4 were detected with high site localization probability. For Ser349, site localization probability was on average 98 %. Notably, we also detected phosphorylation on either Ser338 or Thr342 in the MS data, but not in combination. Moreover, there was no significant change between the conditions if the phosphorylation sites could be detected.

Minor points
- An illustration of the experimental design for the bystander BRET assay would be helpful for clarity.

We agree and have added an illustration in a **new supplementary Figure S1a**.

- The description of chemokine production in the Methods section is very brief and should include more detailed information on the protein production procedure.

As suggested, we have now included more detailed information in the 'Methods' section of the revised manuscript.

- In Fig. 7a, ACKR4 was identified in the volcano plot as upregulated. However, it should not appear in the control group. How was the ratio quantified?

To calculate a ratio for ACKR4, the corresponding background signals were quantified in the mock control samples. This is a conservative approach always enabling the calculation of a ratio also in the complete absence of the protein in the mock condition. Extracted ion currents of three exemplary peptides of ACKR4 identified by MS of transiently transfected HEK293 cells expressing wild-type ACKR4 (labelled wt) or mock (pcDNA3, labelled PC); not stimulated by CCL19 (CTRL), are given below.

ACKR4 peptide sequence: “CIPIFPR” (charge2):

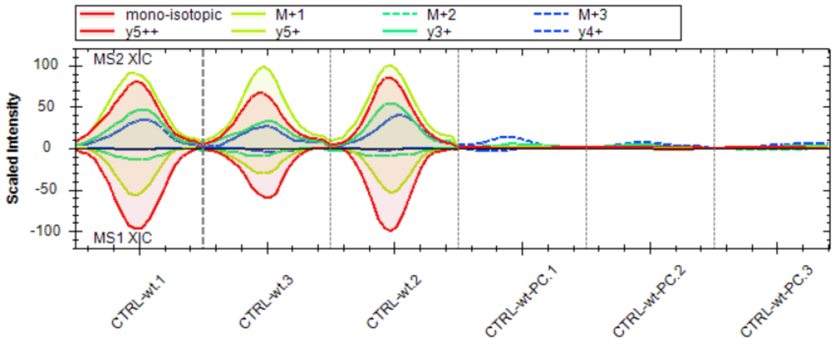

ACKR4 peptide sequence: “NYVMKVAK” (charge2):

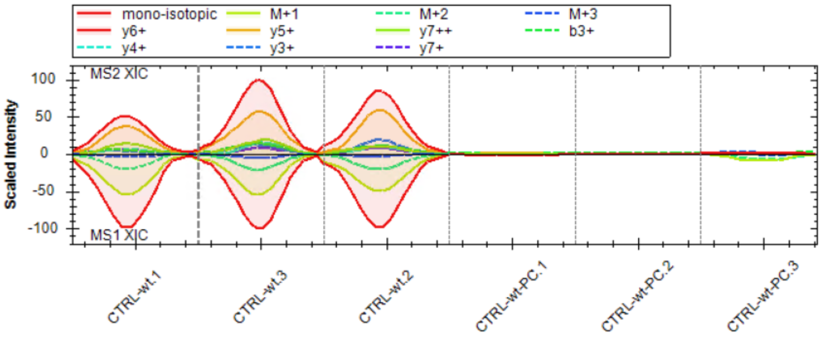

ACKR4 peptide sequence: “AIDIIYSLITSCNMSK” (charge2):

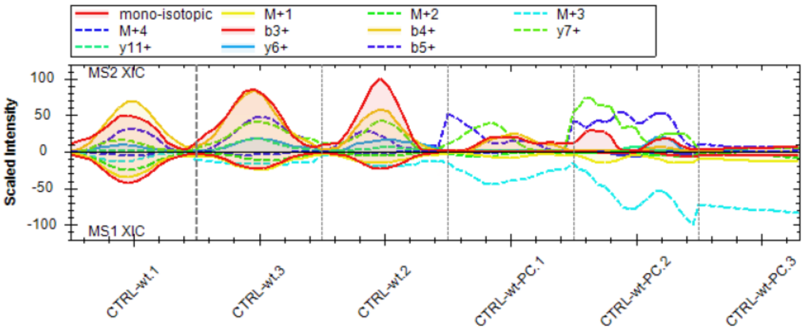

- *Certain sections lack clarity due to loosely structured context. Providing more concise explanations would enhance overall readability.*

We apologise for the loosely structured context. We did our best to provide more concise explanations throughout the manuscript.

- *In several figures (e.g., Fig. 1e, Fig. 2d, Fig. 3f), the X-axis labels are not centered under the corresponding bars. For consistency and readability, it would be preferable to align the labels with the bars.*

We agree and have aligned the labels with the bars in the corresponding Figures.

Reviewer #3 (Remarks to the Author):

In this paper, Gerken et al. studied the interaction of ACKR4 with GRKs and arrestins and its functional consequences. By generating different clusters of phosphorylation-site mutants, they examined how individual phosphorylation sites affect arrestin coupling, ligand uptake, and GRK recruitment. The authors also used mass spectrometry to probe the phosphorylation status of ACKR4 and its interaction patterns. The topic is interesting; however, many of the conclusions are not comprehensive. I also have concerns regarding the GRK recruitment assays.

We thank the Reviewer for the careful assessment and appreciate that the Reviewer considers our study interesting. We carefully addressed all concerns as outlined below in the point-by-point reply.

This study focuses on both steady-state and stimulated conditions, but the experimental definition of "steady state" is not provided. For the BRET and NanoBiT assays, only AUC values are shown in bar graphs. It would be important to present the raw time curves as well, since many results rely on different basal levels and changes before and after stimulation.

We thank the Reviewer for giving us the opportunity to clarify and specify these important aspects. We agree with the Reviewer and now provide the experimental definition of "steady-state", which is "in the absence of a ligand", in lane 139 of the revised manuscript.

P2P Figure 3a-d depict the time-resolved RLU values derived from one exemplary (out of three) experiment with technical duplicates using graded concentrations of CCL19. Data derived from three independent experiments were used to prepare panel **a** of Figure 1. For data analysis, signals from CCL19-stimulated cells were divided by signals from the negative control (PBS; ΔRLU) and subsequently, the area under the curve (AUC) was determined for quantification and hence, AUC values represent ligand-induced, integrated signals over time. In case differences in RLU values before addition of or in the absence of chemokine were observed, those differences were quantified and depicted separately as basal or steady-state activities. We now also provide a **new supplementary Figure S1c** in which we present time-resolved curves for graded concentrations of CCL19 from an exemplary experiment and a **new supplementary Figure S1b** illustrating how AUC values were calculated. By reducing the complexity of the data derived from several independent experiments and presenting AUC and, if pertinent, basal activities, we think that presenting bar graphs representing the mean values and deviations is the optimal option for a better understanding of the main message of the data. The data for all experiments are included in the "Source Data" file and the raw data will be made available on the open research repository Zenodo once the paper is accepted for publication.

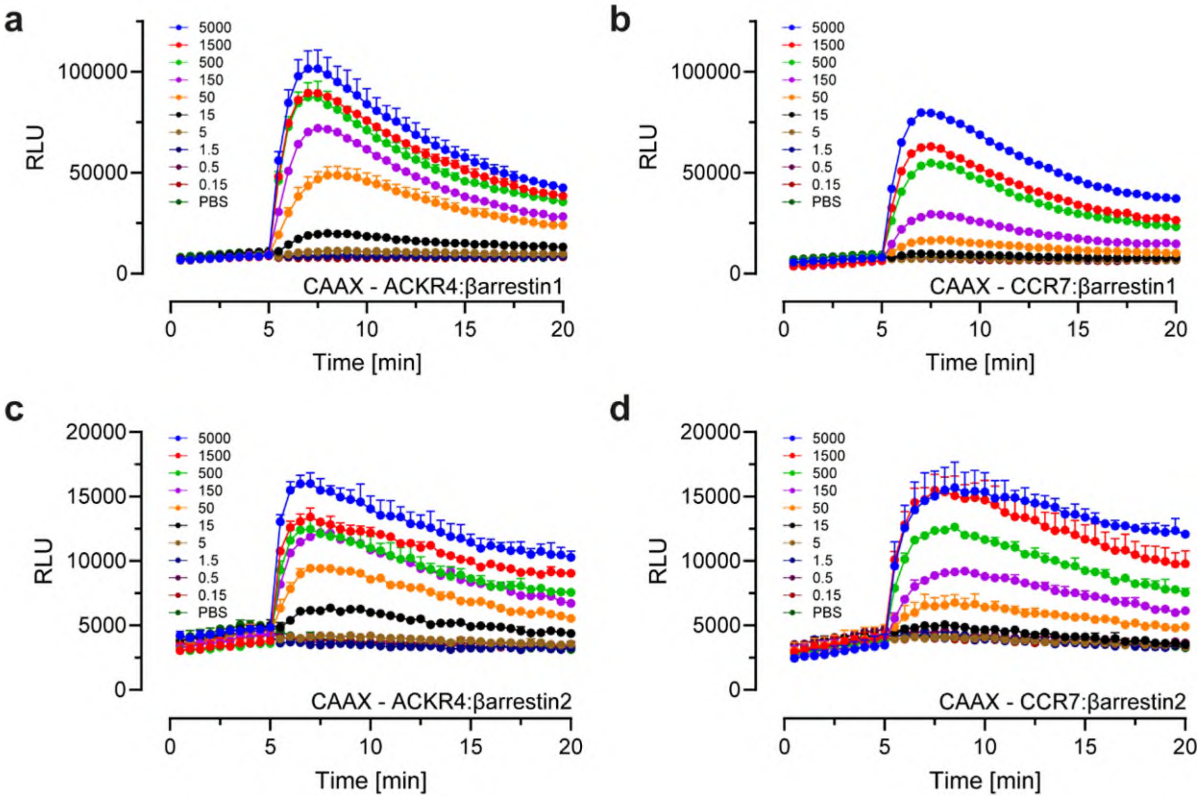

P2P Figure 3: RLU changes over time before and after CCL19 stimulation as assessed by the bystander split-luciferase assays (related to Figure 1a). a-d Increase in bioluminescence upon addition of graded CCL19 concentrations (written in nM) in HEK293 cells expressing ACKR4 or CCR7 together with βarrestin1 or βarrestin2; exemplary replicate depicting raw data before quantification, mean ± SD of technical duplicates.

Among the phosphorylation clusters, cluster 1 is particularly interesting, as it exhibits opposite effects on receptor surface expression and CCL19 uptake compared to the ST/A mutant. Additionally, it shows increased basal interaction with both arrestins and enhanced ligand-independent receptor internalization. The authors, however, did not propose any possible explanations for these findings.

We agree that the Cluster 1 mutant is an interesting one. Indeed, the ST/A mutant is less expressed at the cell surface and less efficient in CCL19 uptake as compared to wild-type ACKR4. By contrast, CCL19 uptake by the Cluster 1 mutant is enhanced, whereas surface expression is reduced (**Figure 3b,c**). Notably, the pre-association of both βarrestins with CAAX at the plasma membrane was significantly higher in cells expressing the Cluster 1 mutant as compared to wild-type ACKR4 under steady-state, ligand free conditions (**Figure 3f**). This suggests that, in the absence of ligand, Cluster 1 mutant cycles faster due to enhanced pre-coupling with βarrestins, i.e. as soon as the Cluster 1 mutant reaches the plasma membrane it interacts with βarrestins resulting in fast receptor internalisation. We have provided the possible explanation in lanes 197-198 by stating "*This observation hints towards an elevated steady-state trafficking activity of the Cluster 1 mutant*". This notion is supported by the enhanced ligand-independent internalisation of the Cluster 1 mutant shown in **Figure 3h**.

I also have reservations about the GRK recruitment assays. According to the authors, phosphorylation sites are critical for GRK2/3 and GRK6 recruitment, since mutating all of them abolishes most interactions; therefore, the ST/A mutant was used as an internal basal control (Fig. 4a). However, the prevailing model in the field is that

GRKs are recruited to the membrane by the activated GPCR core through their N-terminal helix, not by the C-terminal phosphorylation sites.

We agree with the Reviewer that the prevailing model for canonical GPCRs is that GRKs are recruited to the membrane by the activated receptor core to subsequently phosphorylate the receptor's tail. Moreover, the recruitment of GRK2/3 to the activated canonical GPCR is linked to G protein activation as the liberated Gβγ subunit of the heterotrimeric G protein facilitates GRK2/3 translocation to the membrane as described in the introduction of our manuscript (lane 64ff). As atypical chemokine receptors do not activate G proteins, the prevailing model established from canonical GPCR signalling might not directly apply to the ACKR family. We are not aware of a study showing how GRKs precisely interact with ACKRs. However, there is clear evidence that GRKs are recruited to ACKRs to phosphorylate the receptor prior to βarrestin binding. At present, we do not know with which domains ACKR4 interacts with GRKs but observed that GRKs were barely recruited to the plasma membrane in cells expressing the ACKR4 ST/A mutant. Notably, impaired recruitment of GRK2/3 to a C-terminal truncated variant of ACKR4 (ACKR4t) have been published using the standard BRET assay, where ACKR4t is fused to EYFP and GRK2/3 are fused to nLuc (Matti, et al., doi: 10.3389/fimmu.2020.00720). In line with this, Saaber, et al. (doi: 10.1016/j.celrep.2019.01.049) showed that ACKR3 ST/A and ACKR3 deltaC also failed to recruit GRK2 and GRK3 in response to CXCL12 stimulation using a βgalactosidase complementation assay. Moreover, similar results were also described for the canonical GPCR μ opioid receptor (MOR) and its ST/A mutant (Saaber, et al., doi: 10.1016/j.celrep.2019.01.049) (see below, Figure 1B taken from Saaber, et al.). Remarkably, Chen & Tesmer have speculated that "GRKs could potentially also have tail and core interactions with receptors analogous to those of βarrestins" (doi: 10.1016/j.jbc.2022.102279) based on Cryo-EM data obtained from rhodopsin in complex with GRK1 (doi: 10.1038/s41586-021-03721-x). Nevertheless, we have removed the subordinate clause that stated the ST/A mutant was used as an internal basal control, and included the information that our results align with a previous observation regarding ACKR3 (lane 219ff).

Figure 1B from Saaber, et al., doi: 10.1016/j.celrep.2019.01.049 [REDACTED]

Indeed, a recent study on ACKR3 phosphorylation (PMID: 40399676) showed that blocking the activated core inhibited GRK2/GRK5 phosphorylation. The GPCR C-tail is flexible, accessible to GRKs, and does not undergo major conformational changes upon activation. If phosphorylation sites were the main determinants for ACKR4–GRK interaction, GRKs should be constitutively recruited to the membrane, since the sites are present in the basal state.

We are thankful to the Reviewer for this comment as it fully supports our finding showing basal interaction of GRKs with the plasma membrane of ACKR4 expressing cells as assessed in the bystander assay (**Figure 4b**), and ligand stimulation recruit more GRKs to the plasma membrane.

At line 223, the authors claim that they did not detect GRK5 recruitment because GRK5 directly interacts with the plasma membrane through a C-terminal amphipathic helix. However, this helix is conserved in the GRK4 subfamily, which includes GRK4, GRK5, and GRK6. GRK6 also contains a lipid modification that further promotes its membrane localization. It is very surprising that the author could observe GRK6, but not GRK5, recruitment. A more reasonable explanation and more experimental validation is necessary.

Indeed, GRK5 and GRK6 both own the conserved C-terminal amphipathic helix. Notably, GRK6 consists of four splice variants in mice, three of which differ at the C-terminus (Moepps, et al., doi: 10.1006/geno.1999.5901). Isoforms A and B comprise the C-terminal amphipathic helix. GRK6 isoform A possesses three additional C-terminal palmitoylation sites, which enhances the propensity to be associated with the plasma membrane, as its deletion has been shown to render the kinase more cytosolic (Jiang, et al., doi: 10.1091/mbc.E07-01-0013). Isoform B lacks the C-terminal palmitoylation sites and isoform C lacks both the palmitoylation site and the amphiphilic helix (Gurevich, et.al., doi: 10.1016/j.pharmthera.2011.08.001). Isoforms A,B,C in mice have orthologues in human. Mouse isoform D likely lacks catalytic activity and might represent a naturally occurring inhibitor of other GRKs (Moepps, et al., doi: 10.1006/geno.1999.5901). In the present study, we have used human GRK6 isoform B as now specified in the corresponding plasmid table.

In our experimental condition, we observed that the kinase can be recruited to the plasma membrane upon ACKR4 stimulation.

GRK recruitment is known to activate kinase activity, but it is not clear that kinase activity in turn regulates GPCR coupling. It is interesting that the authors observe decreased ACKR4 binding with kinase-dead GRKs. Since GRK recruitment assays, especially for GRK5/6, are notoriously challenging, the raw data should be shown, and these observations should be validated with additional approaches.

We share the opinion of the Reviewer that the decreased ACKR4 interaction with kinase-dead GRKs is interesting. Similar results have also been shown for ACKR3 regarding GRK2 (Saaber, et al., doi: 10.1016/j.celrep.2019.01.049) as discussed above and stated in lanes 241-242 of the revised manuscript. **P2P Figure 4** depicts the raw data from an exemplary experiment. The data for all experiments are included in the “Source Data” file and the raw data will be made available on the open research repository Zenodo once the paper is accepted for publication.

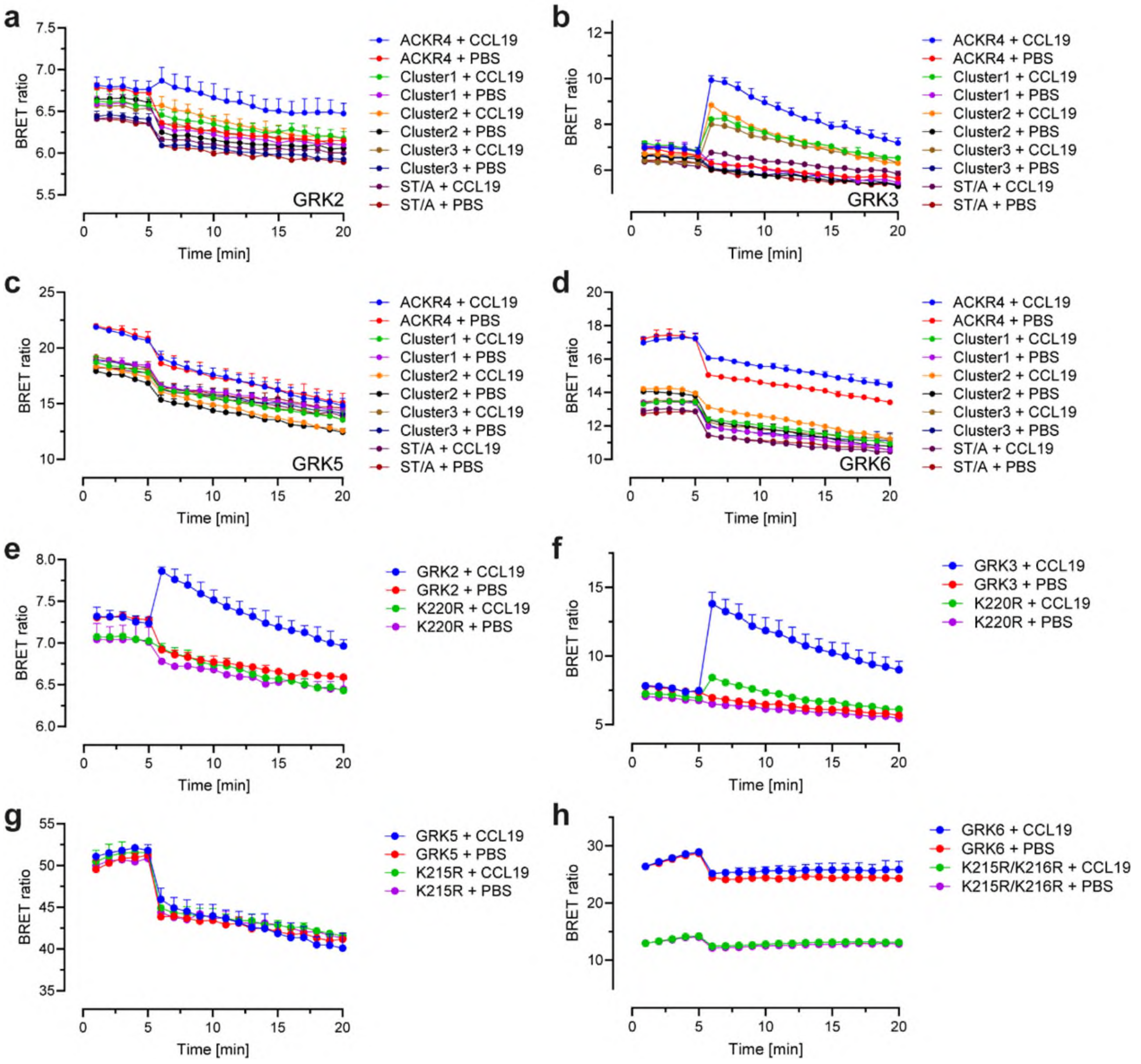

P2P Figure 4: GRK association with the plasma membrane determined in the bystander BRET assays (related to Figure 4). a-d Changes in the BRET ratio before and after stimulation with 1 µM CCL19 or PBS in

HEK293 cells transiently transfected with ACKR4, rGFP-CAAX and GRKs-rLuc8; exemplary replicates illustrating raw data before quantification, mean ± SD of technical duplicates. **e-h** Differences in BRET ratios before and after addition of 1 µM CCL19 or PBS in HEK293 cells expressing ACKR4, rGFP-CAAX and GRKs-rLuc8 WT or kinase-dead mutants; exemplary replicates illustrating raw data before quantification, mean ± SD of technical duplicates.

The authors also claim that "GRK2/3 recruitment occurs independent of G protein activation," but this contradicts their results (Fig. 5f,g). The R587Q GRK2 mutant shows a ~50% reduction in AUC ΔBRET, indicating that GRK2 membrane recruitment is at least partly dependent on Gβγ. This undermines the claim of G protein–independent recruitment. The statement "Steady-state interaction of GRK2 R587Q with CAAX and its recruitment were both reduced, but still intact (Figure 5g)" is unclear. A 50% reduction is not trivial. If the authors propose that free Gβγ arises from sources other than G protein activation, this needs to be explicitly stated.

We thank the Reviewer for pointing this out. We have amended the statement to "Notably, steady-state interaction of GRK2 R587Q with CAAX and its recruitment were significantly reduced (Figure 5g), suggesting that the βγ-subunit of the G protein is somehow involved in membrane association of GRK2."

Indeed, our data using HEK293 cells lacking functional G ptoteins (PTX treated ΔGαq/12/s cells) depicted in **Figure 5d-e** provide clear evidence that GRK2/3 recruitment occurs independent of G protein activation. As described above in the point-by-point reply to the comments from **Reviewer 1**, we now performed a series of additional experiments showing that in ACKR4 pull-downs of unstimulated cells, we can detect co-immunoprecipitated GRK2 and the γ subunit of the G protein (**new Figure 6i**). Moreover, in cells co-expressing GRK2-nLuc, Gβγ-cpVenus/$G\alpha_{i1}$ together with ACKR4, we found enhanced ΔBRET signals between GRK2 and Gβγ, but not between GRK2 R587Q and Gβγ, upon CCL19 stimulation (**new Figure 6a and supplementary Figure S3a**). Importantly, this experiment does not allow discriminating between free Gβγ and inactive Gαβγ. In fact, data shown in Figure 5a-b clearly indicate that ACKR4 stimulation does not activate G proteins. Collectively, our co-immunoprecipitation and proximity-based experiments corroborate our conclusion that GRK2 interacts with the G protein - without activating it - and form a ternary complex with ACKR4 in its ligand-free state.

The phosphorylation-site identification is also unsatisfactory. The antibody-based detection of pS338/pT342-ACKR4 provides very limited information. In fact, mutating these two residues (cluster 2) has little effect on CCL19 uptake or arrestin recruitment. Thus, this assay contributes little to the overall story. As noted in lines 279–281, the only solid conclusion is that S338 and T342 can be phosphorylated; no statements should be made about the full receptor, as other sites may also be phosphorylated.

To the best of our knowledge, the 7TM0315A antibody recognising pS338/pT342 is the sole antibody detecting ACKR4 phosphorylation, hence we used it for our study. We are the first to show that ACKR4 is phosphorylated under steady-state conditions and that pS338/pT342 phosphorylation is enhanced upon CCL19 stimulation. We are aware that this information is limited, which motivated us to take an additional mass-spec approach to identify other GRK phosphorylation sites.

The mass spectrometry analysis is likewise limited. Despite extensive analysis, only one phosphorylation site was identified. It is well established that GRKs typically phosphorylate GPCRs at multiple sites. Indeed, the authors

themselves show in Fig. 6a,b that both S338 and T342 can be phosphorylated. The significance of identifying just one site is questionable. The conclusion that a single phosphorylation site can account for increased GRK2/3 coupling is not convincing, particularly in light of the concerns noted above.

We agree that GPCR phosphorylation involves multiple sites. Our mass spec analysis applied stringent filtering (>75 % localization probability), and Ser349 was identified with high confidence (average 98 %, as discussed above in the point-by-point reply to the comments from **Reviewer 2**). While Ser349 was emphasized due to robust detection and functional validation (**Figure 8e**), phosphorylation at Ser338 and Thr342 was also detected, but not combined. This is consistent with our antibody-based evidence (**Figure 7a,b**), but without significant changes in mass spec data. We do not claim Ser349 alone explains GRK2/3 coupling; rather, it contributes within a broader phosphorylation pattern, which is typical for GPCRs.

Reviewer #4 (Remarks to the Author):

I co-reviewed this manuscript with one of the reviewers who provided the listed reports. This is part of the Nature Communications initiative to facilitate training in peer review and to provide appropriate recognition for Early Career Researchers who co-review manuscripts.

We thank the Reviewer for contributing to the constructive feedback and suggestions, which have helped to improve our manuscript.

Thank you very much for the time and effort in handling our manuscript and its revision. We are delighted that Reviewer 1 has recommended its publication. We are thankful to Reviewers 2-4 for their careful evaluation of our revised manuscript and pleasedto see that we adequately addressed the majority of comments in the first round of revision and do not request additional experiments for the second round of revision.

We have thoroughly revised our manuscript by following the Reviewers' remaining recommendations as outlined in a detailed point-by-point reply to their comments. *The Reviewers comments are italicized*, and our responses are highlighted in blue text. Changes in the revised manuscript are highlighted in yellow.

We thank you and the Reviewers and hope that this revised version of our manuscript will now be accepted for publication in *Nature communications*.

REVIEWER COMMENTS

Reviewer #1 (Remarks to the Author):

I thank the authors for their careful assessment and diligent performance of the additional experiments. The revised manuscript has been significantly improved by the inclusion of key data, such as the BRET/Co-IP assays and the validation in native fibroblasts. These additions have successfully addressed all my previous concerns. I am satisfied with the current version and believe it now meets the high standards for publication.

We are pleased to read that we have successfully addressed all comments and concerns. We thank the Reviewer for the constructive and positive feedback on our work.

Reviewer #2 (Remarks to the Author):

The authors have appropriately responded to the majority of our previous comments, and I appreciate the additional experiments conducted to address the key points raised. The inclusion of data from human fibroblasts with endogenously expressed ACKR4 provides valuable support for the role of GRK2/3 in chemokine uptake and significantly strengthens the physiological relevance of the study.

We thank the Reviewer for appraising our revised manuscript and for recognizing the strength of our study's physiological relevance.

However, despite these improvements, I remain concerned regarding specific data interpretations, particularly in Figure 7h. While the authors attempted to address the potential outlier using various statistical tests (Two-way ANOVA, Spearman's test, and Shapiro-Wilk), these methods are statistically inappropriate for identifying outliers in a small sample size (n=3). To clarify that Two-way ANOVA compares group means rather than individual data points; Spearman's test assesses correlation between variables; and the Shapiro-Wilk test lacks sufficient power to determine normality in such a limited dataset. Consequently, the statistical justification provided is invalid. Instead of emphasizing a distinct effect of GRK2 in enhancing CCL19 uptake based on this data, I would suggest the authors modify their statement to reflect a more neutral interpretation.

We thank the Reviewer for the additional explanation on the concern regarding specific data interpretation. We agree and followed the suggestion of the Reviewer and have modified the description of the data shown in Figure 7h and now provide a more neutral interpretation of the data.

Additionally, while ACKR4 phosphorylation and interaction patterns are emphasized throughout a significant portion of the manuscript, the specific functional relevance of the three phosphorylation sites (Ser338, Thr342, and Ser349) remains somewhat ambiguous. I suggest the authors explicitly discuss this aspect in the Discussion section. It would be beneficial to acknowledge the limitations of antibody-based detection and contextualize how these findings fit within the broader, and often heterogeneous, phosphorylation landscape of GPCRs.

We understand the point on the specific functional relevance of the three phosphorylation sites. Following the suggestion of the Reviewer, we now discuss this aspect in lanes 523-538 of the discussion. We now also discuss the results obtained based on antibody-mediated detection in relation to the results obtained by mass spectrometry and contextualized our findings within the broader phosphorylation landscape of GPCRs.

Reviewer #3 (Remarks to the Author):

I have strong reservations regarding the bystander BRET assay, particularly after examining the original data plots provided by the authors in their response. For GRK5 and GRK6, the bystander BRET approach is not appropriate. GRK5/6 are predominantly localized at the plasma membrane under basal conditions; therefore, there is no cytosol-to-membrane translocation to be monitored by this assay. Consistent with this, the authors' original data show a decrease in BRET signal rather than the typical increase observed for GRK2/3 and arrestins. This decrease in BRET signal could result from a combination of GRK5/6 trafficking dynamics and competition with endogenous GRK5/6, and thus does not directly reflect a specific interaction between ACKR4 and GRK5/6. Consequently, I do not believe that conclusions drawn from this assay for GRK5/6 are reliable.

We fully agree with the Reviewer that GRK5 and GRK6 are predominantly expressed at the plasma membrane and that therefore the bystander BRET assay is inappropriate to monitor translocation of GRK5/6 from the cytoplasm to the plasma membrane. We primarily opted for the bystander BRET assay to monitor the recruitment of cytosolic β-arrestins and GRK2/3 to the plasma membrane as the conventional direct BRET assay relies on tagged receptors, which we have previously found to jeopardize ACKR4 functions (PMID: 39623381).

We apologize for having misinterpreted the original request and for not having fully addressed this concern of the Reviewer. We originally understood that the Reviewer had requested the "raw data", meaning, for us, data that had not been subjected to any baseline correction, normalization or interpretation. The decrease in the BRET ratio observed in the raw data is due to the experimental setup, where PBS, or chemokine (in PBS), is added to the well, resulting in a 1:1 dilution of the sample and thus reducing the amount of cells present in the z-level that is acquired using the Tecan Spark plate reader (**P2P Figure 1&2, left panels**). In fact, for GRK3 as an example, the decrease of the BRET ratio for ACKR4 upon PBS addition reflects the dilution of the signal as a consequence of the solvent (PBS) addition. Upon addition of CCL19 to the ACKR4 sample, the BRET ratio exceeds the dilution effect and the signal resulting in an overall robust increase in the BRET ratio. The ΔBRET ratio reflecting the ligand-mediated signal normalized to PBS, is calculated as the fold change of the BRET ratio of the ligand-stimulated sample over time divided by the corresponding BRET ratio of the PBS-stimulated sample (**P2P Figure 1&2, middle panels**). CCL19-mediated changes in the ΔBRET ratio between rGFP-CAAX and GRK2-rLuc8 or GRK3-rLuc8 reflect the recruitment of GRK2/3 to the plasma membrane of ACKR4 expressing cells in response to ligand stimulation. Using the same assay, we observed slight, but reproducible changes in the ΔBRET ratio between GRK6-rLuc8 and rGFP-CAAX, but not between GRK5-rLuc8 and rGFP-CAAX, in response to CCL19 stimulated ACKR4. The bystander BRET approach thus seems to provide some hints for changes in the proximity between GRK6-rLuc8 and rGFP-CAAX and/or the orientation of these two molecules to each other upon ACKR4 engagement at the plasma membrane. Notably, we observed that the basal, steady-state, prior to stimulation BRET ratio (without normalization) between rGFP-CAAX and GRK6-rLuc8 or rGFP-CAAX and GRK5-rLuc8 were lower for cluster mutants and the ST/A mutant as compared to wild-type ACKR4 (**P2P Figure 1, left panels**). It remains speculative, but these observations open the possibility that the proximity of GRK5/6-rLuc8 with rGFP-CAAX and/or their orientation differ depending on whether ACKR4 or Ser/Thr mutants thereof are expressed in cells.

The **right panels** of **P2P Figure 1&2**, illustrates the area under the curve (AUC) of the corresponding ΔBRET ratio values over time.

P2P Figure 1&2 provided below depicts the CCL19 (or PBS)-mediated changes as BRET ratio (raw data), as ΔBRET ratio (normalized to PBS), and as AUC of ΔBRET ratio between GRKs-rLuc8 and rGFP-CAAX for all four kinases.

We respect the Reviewer's reservations regarding the use of the bystander BRET assay for GRK5/6 and, consequently, have now removed all data on GRK5 and GRK6 from the main Figure 4 and only present data on GRK2 and GRK3. We have now also refrained from drawing conclusions on GRK5/6 using the bystander BRET assay and only describe notable observations for GRK5/6 in the revised manuscript and depict the corresponding data as supplementary Figure S3.

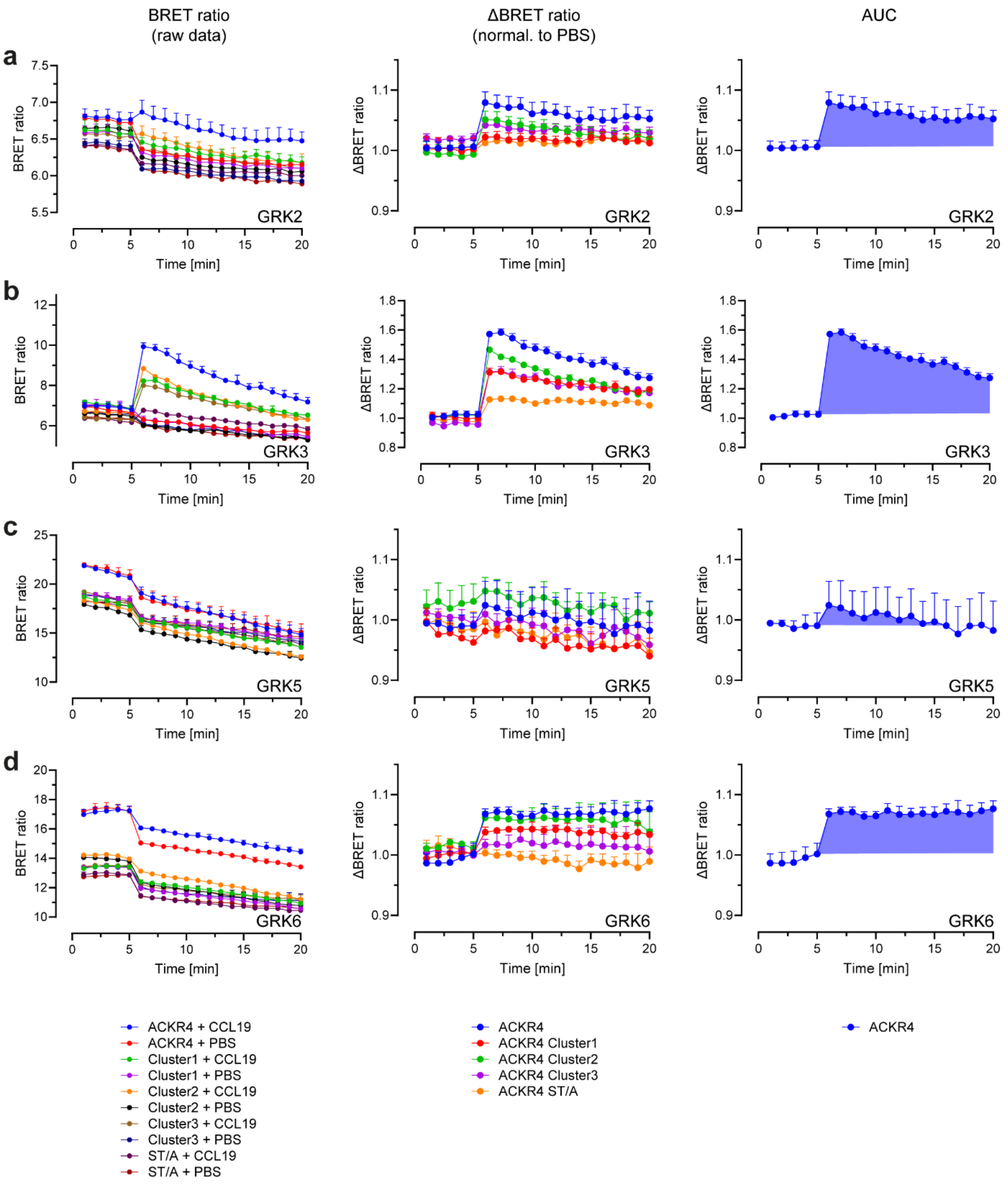

P2P Figure 1: CCL19-mediated changes in (Δ)BRET ratios in HEK293 cells expressing ACKR4 and its mutants. HEK293 cells expressing ACKR4 or receptor variants were co-transfected with rGFP-CAAX and different GRKs fused to rLuc8. Subsequently, CCL19 in PBS or PBS alone were added to the well (thereby diluting the sample) and changes over time in the BRET ratio (raw data; left panels), changes in the ΔBRET ratio (normalized to PBS; middle panels) and exemplary area under the curve (AUC) illustration considered for quantification (right panels) are shown for GRK2 (**a**), GRK3 (**b**), GRK5 (**c**) and GRK6 (**d**). Exemplary replicates, mean ± SEM of technical duplicates are shown.

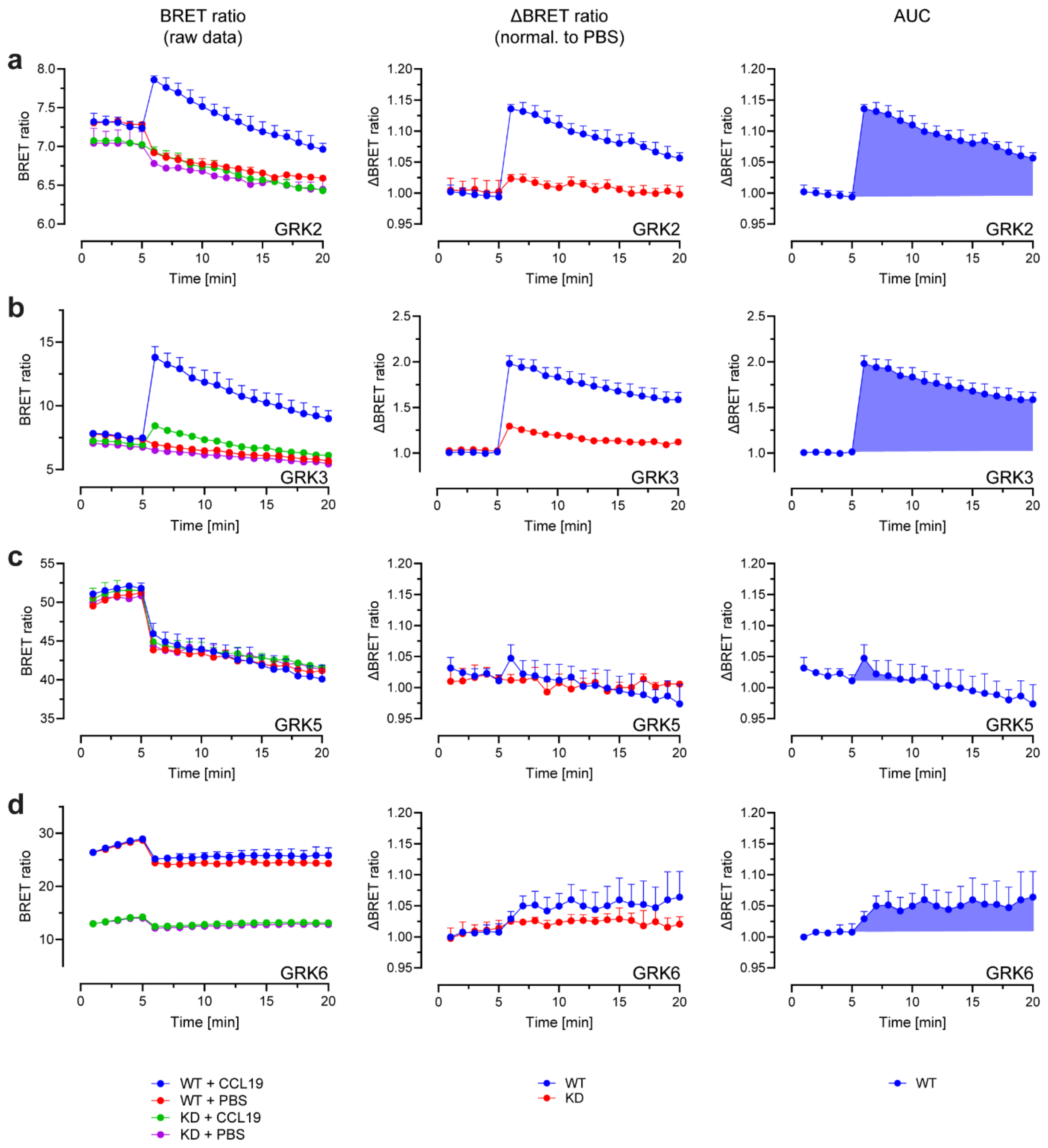

P2P Figure 2: CCL19-mediated changes in (Δ)BRET ratios in HEK293 cells expressing ACKR4 together with wild-type or kinase-dead variants of GRKs. HEK293 cells were co-transfected with ACKR4, rGFP-CAAX and different wild-type or kinase dead (KD) variants of GRKs fused to rLuc8. Subsequently, CCL19 in PBS or PBS alone were added (thereby diluting the sample) and changes over time in the BRET ratio (raw data; left panels), changes in the ΔBRET ratio (normalized to PBS; middle panels) and exemplary area under the curve (AUC) illustration considered for quantification are shown for GRK2 (**a**), GRK3 (**b**), GRK5 (**c**) and GRK6 (**d**). Exemplary replicates, mean ± SEM of technical duplicates are shown.

Along the same lines, the GRK2 response appears very subtle. The most convincing interaction observed is between GRK3 and ACKR4. I strongly recommend that the authors present the full time-course data alongside the AUC analysis and clearly indicate how the AUC was calculated. For example, based on P2P Fig. 4h, I do not observe a significant difference between GRK6 WT and the kinase-dead variant.

We agree with the Reviewer that the changes in the ΔBRET ratio were most profound for GRK3, followed by GRK2. As recommended, we now present the full time-course in CCL19-induced changes in the ΔBRET ratio for GRK2 and GRK3 in the **revised Figure 4a and 4b**. Full time-courses for all four GRKs for comparison are now shown as **new supplementary Figure S3a**, alongside with the AUC analysis for GRK3 depicted as **new supplementary Figure S3b**. The difference between GRK6 WT and KD of the ΔBRET ratio (normalized to PBS) can be appreciated in P2P Figure 2d provided above. Notably, the most striking difference between GRK6 WT and KD is in the BRET ratio, which is profoundly lower under basal conditions, i.e. before addition of PBS and/or CCL19. As recommended and described above, we no longer show data on GRK5 and GRK6 obtained with the bystander BRET assay in the main figure, and have moved them to supplementary Figure S3.

Reviewer #4 (Remarks to the Author):

I co-reviewed this manuscript with one of the reviewers who provided the listed reports. This is part of the Nature Communications initiative to facilitate training in peer review and to provide appropriate recognition for Early Career Researchers who co-review manuscripts.

We thank the Reviewer and highly appreciated the constructive and positive feedbacks and suggestions.
